# Functional Recovery of Adults Following Acute COVID-19: A Systematic Review and Meta-Analysis

**DOI:** 10.1093/ptj/pzae023

**Published:** 2024-02-22

**Authors:** Sophie Middleton, Christos V Chalitsios, Tanvi Mungale, Zeinab M Hassanein, Alex R Jenkins, Charlotte E Bolton, Tricia M McKeever

**Affiliations:** Nottingham Biomedical Research Centre, Clinical Sciences Building, University of Nottingham, City Hospital Campus, Hucknall Road, Nottingham, UK; Centre for Respiratory Research, Translational Medical Sciences, School of Medicine, Clinical Sciences Building, University of Nottingham, City Hospital Campus, Hucknall Road, Nottingham, UK; Department of Respiratory Medicine, Nottingham University Hospital NHS Trust, City Hospital, Nottingham, UK; Nottingham Centre for Epidemiology and Public Health, Lifespan and Population Health, School of Medicine, University of Nottingham, Nottingham, UK; Nottingham Biomedical Research Centre, Clinical Sciences Building, University of Nottingham, City Hospital Campus, Hucknall Road, Nottingham, UK; Nottingham Centre for Epidemiology and Public Health, Lifespan and Population Health, School of Medicine, University of Nottingham, Nottingham, UK; Nottingham Biomedical Research Centre, Clinical Sciences Building, University of Nottingham, City Hospital Campus, Hucknall Road, Nottingham, UK; Nottingham Biomedical Research Centre, Clinical Sciences Building, University of Nottingham, City Hospital Campus, Hucknall Road, Nottingham, UK; Centre for Respiratory Research, Translational Medical Sciences, School of Medicine, Clinical Sciences Building, University of Nottingham, City Hospital Campus, Hucknall Road, Nottingham, UK; Department of Respiratory Medicine, Nottingham University Hospital NHS Trust, City Hospital, Nottingham, UK; Nottingham Biomedical Research Centre, Clinical Sciences Building, University of Nottingham, City Hospital Campus, Hucknall Road, Nottingham, UK; Nottingham Centre for Epidemiology and Public Health, Lifespan and Population Health, School of Medicine, University of Nottingham, Nottingham, UK

**Keywords:** COVID-19, Functional, Muscle Strength, Physical, Recovery, Walking

## Abstract

**Objective:**

This systematic review and meta-analysis aimed to investigate the objective, functional recovery of patients more than 3 months after acute coronavirus disease 2019 (COVID-19) infection.

**Methods:**

Comprehensive database searches of EMBASE, PubMed/MEDLINE, Cochrane COVID-19 Study Register, CINAHL, and Google Scholar in accordance with the Preferred Reporting Items for Systematic Reviews and Meta-Analyses statement were carried out until October 19, 2022. Data were extracted and agreed in duplicate. Data were narratively synthesized, and a series of meta-analyses were performed using the random-effects inverse variance method.

**Results:**

One-hundred six papers covering 20,063 patients, who were either hospitalized or not hospitalized with acute COVID-19 and were followed-up between 3 and 24 months, were included. Percentage predicted 6-minute walk distance at 3 months to <5 months was 84.3% (95% CI = 79.2–89.3; *n* = 21; I^2^ = 98.3%) and 92.5% (95% CI = 89.8–95.3; *n* = 9; I^2^ = 94.5%) at ≥11 months. Cardiopulmonary exercise testing revealed the percentage predicted peak oxygen consumption rate ($peak\dot{\mathsf{V}}{\mathsf{o}}_{\mathsf{2}}$) at 3 months to <5 months was 77.3% (95% CI = 71.0–83.7; *n* = 6; I^2^ = 92.3%) and 95.4% (95% CI = 87.1–103.6; *n* = 2; I^2^ = 77.3%) at ≥11 months. Mean handgrip strength was greatest at ≥11 months at 31.16 kg (95% CI = 19.89–42.43; *n* = 2; I^2^ = 98.3%) of all time points. All analyses showed marked heterogeneity.

**Conclusion:**

Patients have reduced physical function more than 3 months after COVID-19 infection. Better physical function in multiple physical domains is found after a longer recovery time.

**Impact:**

Physical function as measured by the 6-minute walk test, hand grip strength, and cardiopulmonary exercise testing is reduced at 3 months after COVID-19 infection and can remain over 11 months of follow-up. This protracted recovery following acute COVID-19 infection supports the need to assess physical function at any clinical follow-up, and further research into rehabilitation programs and intervention for patients who have not recovered.

## Introduction

To date there have been more than 760 million reported cases of COVID-19 worldwide. The outlook for acute illness has drastically changed compared to 2020 and the vast majority now survive. ^1^ However, up to a year after acute, hospitalized infection, only 28.9% of adults in the UK feel completely recovered,^2^ and an estimated 2 million people in the United Kingdom alone have self-reported “long COVID.”^3^ Studies have shown that both patients who are hospitalized and those who are not hospitalized for the acute period continue to experience multisystemic symptoms such as fatigue, dyspnea, myalgia, and cognitive disturbances at follow-up.^4^ This is disproportionate with the smaller number of participants with abnormal lung function, thoracic imaging, and systemic inflammation.^5^^,^^6^ The terms long COVID or post-COVID syndrome have been used to describe ongoing symptoms 3 months after initial COVID-19 infection that cannot be explained by another diagnosis.^7^ This is still loosely defined, encompassing a large number of symptoms.

Examination of a person’s physical function is useful to evaluate the impact of an acute illness as well as set appropriate goals for physical therapy. It is known that hospitalization can lead to reduced physical and cognitive function, especially after prolonged admission or critical illness.^8^ It is yet unclear what physical function is during COVID-19 recovery. Early studies have shown reduced physical function after COVID-19 on self-reported questionnaires such as the EQ-5D and Medical Research Council dyspnea scale breathlessness score and objective physical testing such as the composite Short Physical Performance Battery (SPPB).^9^ Patients were unable to return to their previous level of work and activities and had a poorer quality of life.^6^ A better understanding of functional status after COVID-19 could aid the design and implementation of interventions focused on the recovery of disability to improve the quality of life of this population. This systematic review and meta-analysis aimed to investigate the objective, functional recovery of patients who were not hospitalized, and those who were hospitalized more than 3 months after acute COVID-19 infection.

## Methods

### Data Sources and Searches

This systematic review was registered with PROSPERO (CRD42022367436)^10^ and followed Preferred Reporting Items for Systematic Reviews and Meta-Analyses guidelines for reporting systematic reviews.^11^ The following databases were searched on October 19, 2022 for relevant studies: EMBASE, PubMed/MEDLINE, Cochrane COVID-19 Study Register, CINAHL, and Google Scholar. The search strategy included synonyms for COVID-19, recovery, and functional outcome measures. The full search strategy can be found in Supplementary Text 1.

### Study Selection

Following collation of search results and removal of duplicate citations, title and abstract screening was undertaken by 1 reviewer to eliminate irrelevant papers. Full papers were reviewed in duplicate for selection by 2 reviewers (S.M., T.M.M.). Studies were included if they contained participants over 18 years old, had a sample size of at least 30 patients, incorporated patients that had a clinical or polymerase chain reaction diagnosis of COVID-19, and had a mean follow-up time of at least 3 months after hospital discharge or acute infection in patients who were not hospitalized. Studies were excluded if their primary outcome measures were focused solely on nonfunctional outcomes of COVID-19 or only used functional questionnaires and not physical testing.

The main outcomes collected were walking tests such as the 6-Minute Walk Test (6MWT) and incremental shuttle walk test. The percentage predicted distance walked in the 6MWT was, where stated, calculated with regard to age, sex, weight, and height and the observed values were compared to the predicted values. Physical strength outcomes included handgrip strength (HGS) and quadriceps strength using a dynamometer. Performance tests collected include the 1-minute sit-to-stand test and the composite SPPB. Exercise capacity measured using cardiopulmonary exercise testing was collected with peak $\dot{\mathsf{V}}{\mathsf{o}}_{\mathsf{2}}$ measured as the highest amount of oxygen consumed at peak exercise.

### Data Extraction and Quality Assessment

Data extraction was undertaken using a proforma approach by 5 reviewers (S.M., C.V.C., T.M.M., T.M., A.R.J.) and data agreed for each paper by 2 reviewers. Where disagreement remained, a third reviewer was included (C.E.B. or T.M.M.). The proforma included patient selection, sample size, demographics, and study design. The quality of the included studies was determined using an assessment tool based on the Newcastle-Ottawa Scale score and the Downs and Blacks checklist.^12^^,^^13^

**Figure:**
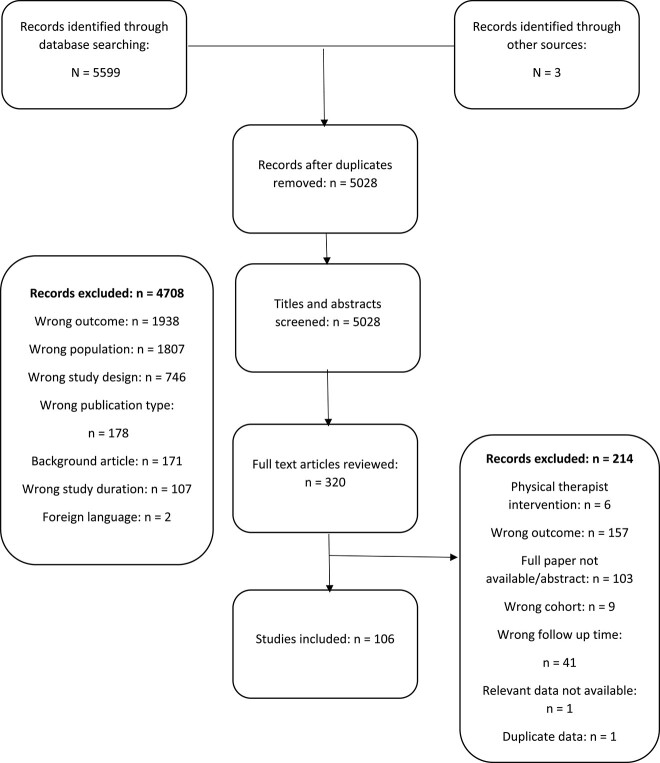
Paper selection flow chart.

### Data Synthesis and Analysis

Narrative synthesis of evidence was conducted for all included studies. Studies were split into groups according to how their data were presented in their study and where it was not possible to combine them. All follow-up times converted to months in narrative review and supplementary tables by dividing those reported in days by 28 and those reported in weeks by 4 to standardize results. When looking at the chronology of follow-up, there were 3 peaks around 3, 6, and 12 months. To capture this, the studies were grouped considering the time to follow-up after discharge or acute infection (3 months to <5 months, 5 months to <11 months, and ≥ 11 months), and meta-analyses were performed for each group. Summary statistics (means or percentages) were used in meta-analyses where individual patient data on age, sex, and body mass index were not available. Meta-analysis using random effects models was performed to allow for apparent heterogeneity among studies. The generic inverse variance method was used for pooling, and pooled estimates were calculated using proportions with 95% confidence intervals. The percentage of variability in the effect sizes was tested by using the Higgins I^2^ test, with estimates of 25%, 50%, and 75% indicating low, moderate, and high levels of heterogeneity, respectively. If a study reported follow-up at multiple time points with the same cohort, only the first follow-up time point in each follow-up time point group (3 months to <5 months, 5 months to <11 months, and ≥ 11 months) was used in the meta-analysis. If a study contained a rehabilitation intervention, only baseline measurements were used for each cohort and control group. Patients who were hospitalized and those who were not hospitalized were analyzed together unless stated otherwise. All meta-analyses were conducted in R v4.2.2 (R Foundation; Vienna, Austria) using the “meta” and “metafor” packages, and all statistical tests were 2-sided and used a significance level of *P* < .05.

Subgroup analysis was undertaken for the 6MWT to identify causes of heterogeneity. Histograms were made for mean age, percentage of patients hospitalized, and admitted to the intensive care unit. The histograms were then used to divide patients into groups for age, hospitalization, and intensive care admission for further meta-analysis. Meta-analyses were performed for each group using the first follow-up time point for each study.

## Results

The search strategy identified 5599 papers, 106 of which were included in the review^2^^,^^6^^,^^14–117^ (Figure) Ninety-eight papers were used in the meta-analysis.

### Study Characteristics

The majority of studies were observational, and study follow-up time ranged from 3 months to 24 months. Seventy-two papers were from Europe, 19 were from Asia (mainly China [*n* = 14]), 9 were from North America, and 6 were from South America. Participant numbers per publication ranged from 30 to 1733, with 20,063 participants in total across the outcome measures (Suppl. Tab. 1). On average, participants were 58.0% (study range = 5%–88%) men, the mean of the mean ages was 56 (study range = 35–78) years, and for body mass index was 27.9 kg/m^2^ (study range = 22.7–32.9). Most studies reported on only patients who were hospitalized (*n* = 76), but 24 reported both patients who were hospitalized and those who were not hospitalized and 3 reported patients who were not hospitalized only. A full list of outcome measures is shown in Supplementary Table 2. Most studies were of moderate quality with an average quality score of 6.5 (SD = 1.2) (Suppl. Tab. 3). Most cohorts were single-site, hospitalized cohorts without a control group.

### Walking Tests

The most common walking test performed was the 6MWT.^6^^,^^15–20^^,^^23–27^^,^^30^^,^^31^^,^^34^^,^^40^^,^^42–47^^,^^49–55^^,^^59–62^^,^^66^^,^^67^^,^^69^^,^^71^^,^^72^^,^^74^^,^^77^^,^^79^^,^^81^^,^^82^^,^^84^^,^^86^^,^^88^^,^^90^^,^^91^^,^^93^^,^^95–99^^,^^101–105^^,^^107^^,^^108^^,^^110^^,^^113–117^ Meta-analysis demonstrated mean distances walked of 494.57 m (95% CI = 476.84–512.30; *n* = 47; I^2^ = 99.2%) at between 3 months and 5 months and 508.94 m (95% CI = 484.28–533.59; *n* = 19; I^2^ = 98.8%) at ≥11 months (Tab. 1; Suppl. Figs. 1–6). The percentage predicted distance walked was 84.3% (95% CI = 79.2–89.3; *n* = 21; I^2^ = 98.3%) and 92.5% (95% CI = 89.7–95.3; *n* = 9; I^2^ = 94.5%), respectively. There was marked heterogeneity across all meta-analyses (I^2^ > 94%). In a further subgroup meta-analysis, there was no difference in 6MWT distance (6MWD) and percentage predicted distance between patients who were over or under 60 years old, or between those who were hospitalized, those who were not hospitalized, and those who were admitted to the intensive care unit (Suppl. Figs. 21–34).

In studies that could not be included in the meta-analysis, Lam et al reported that 36.0% had a 6MWD of less than the lower limit of normal at a mean of 4.3 months after discharge^65^ and De Lorenzo et al reported that 37.3% of patients had a percentage predicted 6MWD of ≤70% and increased breathlessness after the 6MWT at follow-up at 6 months.^36^ Three studies report walking distance using the incremental shuttle walk test at different time points, with results: 300 m (SD = 198) at a mean of 4.5 months,^35^ 436 m (SD = 260) at 5 months,^85^ and 468.2 m (SD = 267.8) at 13 months.^2^ Bellan et al reported that 31.5% and 7.1% walked a distance shorter than expected for age and sex in the 2-minute walk test at 3.5 months^22^ and 12 months,^21^ respectively.

**Table 1:** Meta-Analysis of Functional Tests at 3 Months to <5 Months, 5 Months to <11 Months, and > 11 Months*^a^*

**Functional Test**	**3 Mo to < 5 Mo**			**5 Mo to < 11 Mo**			$\boldsymbol{\ge}$ **11 Mo**		
	**No. of Studies**	**Effect Size (95% CI)**	**I** ^**2**^ **, %**	**No. of Studies**	**Effect Size (95% CI)**	**I** ^**2**^ **, %**	**No. of Studies**	**Effect Size (95% CI)**	**I** ^**2**^ **, %**
6MWT, m	47	494.57 (476.84–512.30)	99.2	34	470.26 (452.48–488.03)	98.2	19	508.94 (484.28–533.59)	98.8
6MWT, % predicted	21	84.25 (79.21–89.30)	98.3	17	87.04 (81.71–92.37)	98.0	9	92.52 (89.76–95.28)	94.5
Hand grip strength, kg	12	28.45(25.46–31.45)	96.2	8	28.52 (24.74–32.29)	95.4	2	31.16 (19.89–42.43)	98.3
1minSTS, N	3	23.08 (7.43–38.72)	100	2	28.32 (15.87–40.76)	98.8	1		
SPPB, N	3	11.89 (11.63–12.16)	71.3	3	10.97 (9.35–12.58)	99.6	0		

^a^ 1minSTS = 1-min sit-to-stand test; 6MWT = 6-Min Walk Test; SPPB = Short Physical Performance Battery.

### Strength Tests

Physical strength was most commonly measured with a dynamometer as HGS across 18 publications.^33^^,^^37–39^^,^^48^^,^^53^^,^^56^^,^^57^^,^^67^^,^^68^^,^^71^^,^^76^^,^^79^^,^^80^^,^^86^^,^^89^^,^^105^^,^^112^ Fifteen studies were incorporated in the meta-analysis with mean handgrip of 28.45 kg (95% CI = 25.46–31.45; *n* = 12; I^2^ = 96.2%), 28.52 kg (95% CI = 24.74–32.29; *n* = 8; I^2^ = 95.4%), and 31.16 kg (95% CI = 19.89–42.43; *n* = 2; I^2^ = 98%) at 3 months to <5 months, 5 months to <11 months, and ≥ 11 months, respectively (Tab. 1; Suppl. Figs. 7–9). This is unadjusted for age or sex.

Among studies not able to be included in the meta-analysis, Johnsen et al reported that in a mixed sample of patients who were hospitalized and those who were not hospitalized, 28.1% performed below the 25th percentile compared to population-based reference values at 3 months after acute COVID-19 infection.^57^ Lorent et al demonstrated that at 3 months 22.0% of participants had a predicted HGS of <80%, which decreased to 6.6% at 12 months. The significant difference reported in HGS between patients with moderate COVID-19 and those with severe COVID-19 at 3 months was not present at 12 months.^71^ At an average of 3.1 months after hospital discharge, Nanwani et al reported that patients who had been mechanically ventilated in the intensive care unit were more likely to have reduced HGS than those who were not (66% vs 34%; *P* = .0001).^80^

At 6 months after discharge, Wu et al reported a significant difference in muscle strength of the upper and lower limbs between those who had moderate acute COVID-19 and those who had severe acute COVID-19, with 0% and 8.7% having reduced Manual Muscle Test scores (<5), respectively (*P* = .006).^109^ Quadriceps strength measured using a dynamometer was reported to be ≤70% predicted at 3 months in 60.2% of people, decreasing to 32% at 12 months.^71^

### Performance Tests

The 1-minute sit-to-stand test was reported in 6 papers.^31^^,^^32^^,^^39^^,^^53^^,^^57^^,^^92^^,^^111^ In meta-analysis, the average numbers of repetitions were 23.08 (95% CI = 7.43–38.72; *n* = 3; I^2^ = 100%) at between 3 months and < 5 months and 28.32 (95% CI = 15.87–40.76; *n* = 2; I^2^ = 98.8%) at between 5 months and < 11 months (Tab. 1; Suppl. Figs. 10 and 11). Not included in meta-analysis, Combret et al reported an average of 23 (SD = 16.3) repetitions in 94 participants at follow-up at 11 months.^32^ Johnsen et al presented 44% of participants performed below the 25th percentile compared to the general population at follow-up at 3 months.^57^

The SPPB was performed in 6 studies.^2^^,^^21^^,^^22^^,^^33^^,^^79^^,^^85^ Meta-analysis of SPPB at between 5 and < 11 months showed a normal score of 10.97 (95% CI = 9.35–12.58; *n* = 3; I^2^ = 99.6%) across 3 cohorts (Tab. 1; Suppl. Figs. 12 and 13).^33^^,^^85^ Bellan et al reported SPPB altered in 22.3% at 3.5 months^22^ and 18.7% after 12 months.^21^ Evans et al reported SPPB values of <10 in 46.4% at 5 months and 45.1% at 13 months.^2^ Xiong et al found improved functional physical fitness including 30-second chair stand, flexibility, and agility over 12.4 months,^111^ but 29.1% had not yet fully recovered their functional physical fitness by 13.8 months.^112^

One study measured physical activity using an accelerometer worn on the participant’s wrist. At 5 months follow-up, both women and men had low levels of moderate- to vigorous-intensity physical activity. More severe acute illness was associated with a lower volume of physical activity. Those recovering from COVID-19 had lower physical activity than a matched cohort of office workers and a matched group with type 2 diabetes.^83^

### Cardiopulmonary Exercise Test

Cardiopulmonary exercise testing was performed using either a stationary cycle or a treadmill in 18 studies.^14^^,^^17^^,^^29^^,^^31^^,^^40^^,^^41^^,^^56–58^^,^^63^^,^^64^^,^^70^^,^^73^^,^^78^^,^^86^^,^^87^^,^^100^^,^^106^ In incremental cycle cardiopulmonary exercise testing, peak $\dot{\mathsf{V}}{\mathsf{o}}_{\mathsf{2}}$ was 21.38 mL/min/kg (95% CI = 19.58–23.18; *n* = 7; I^2^ = 92.9%) at 3 months to <5 months, and 30.84 mL/min/kg (95% CI = 24.83–36.85; *n* = 6; I^2^ = 97.5%) at 5 months to <11 months. Percentages predicted were 77.3% (95% CI = 71.0–83.7; *n* = 6; I^2^ = 92.3%) at between 3 months and < 5 months and 95.4% (95% CI = 87.1–103.6; *n* = 2; I^2^ = 77.3%) at ≥11 months (Tab. 2; Suppl. Figs. 14–20).

**Table 2:** Meta-Analysis of Cardiopulmonary Exercise Testing (CPET) at 3 Months to <5 Months, 5 Months to <11 Months, and > 11 Months*^a^*

**Functional Test**	**3 Mo to < 5 Mo**			**5 Mo to < 11 Mo**			**>11 Mo**		
	**No. of Studies**	**Effect Size (95% CI)**	**I** ^**2**^ **, %**	**No. of Studies**	**Effect Size (95% CI)**	**I** ^**2**^ **, %**	**No. of Studies**	**Effect Size (95% CI)**	**I** ^**2**^ **, %**
CPET on bike, peak $\dot{\mathsf{V}}{\mathsf{o}}_{\mathsf{2}}$, mL/min/kg	7	21.38 (19.58–23.18)	92.9	6	30.84 (24.83–36.85)	97.5	1		
CPET on bike, % predicted $\dot{\mathsf{V}}{\mathsf{o}}_{\mathsf{2}}$	6	77.34 (70.98–83.71)	92.3	2	95.93 (66.53–125.33)	98.1	2	95.37 (87.12–103.63)	77.3
CPET on treadmill, peak $\dot{\mathsf{V}}{\mathsf{o}}_{\mathsf{2}}$, mL/min/kg	0			4	24.82(19.41–30.22)	97.4	0		
CPET on treadmill, % predicted $\dot{\mathsf{V}}{\mathsf{o}}_{\mathsf{2}}$	0			4	89.08 (78.46–99.70)	94.9	0		

^a^ $\dot{\mathsf{V}}{\mathsf{o}}_{\mathsf{2}}=$ oxygen consumption rate.

## Discussion

This comprehensive systematic review and meta-analysis investigating the functional recovery of adult patients with COVID-19 demonstrates suboptimal physical function across multiple domains including walking, HGS, and cardiopulmonary exercise. Decreased performance in outcome measures such as walking distance can remain over 11 months after acute infection. Better physical function is found after a longer recovery time.

Our study showed that at ≥11 months after COVID-19 infection, 6MWD remained below healthy population average.^118^ 6MWD has been used to measure physical recovery in other respiratory illnesses. In a systematic review of people infected with severe acute respiratory syndrome coronavirus (SARS-CoV) during the 2003 outbreak, 6MWD was 74% to 83% of that recorded for healthy controls at 12 months after hospital discharge. These figures were based on 2 small studies, one of which only included those admitted to intensive care unit, which could explain the poorer outcomes compared to our study.^119^ In another study, patients hospitalized with community-acquired pneumonia demonstrated a quicker recovery time than our study with an average 6MWD of 506 m (SD = 156.1) at 42 days.^120^ In a population with comorbid chronic obstructive pulmonary disease, 6MWD declined by 20.4% during exacerbation and persisted in the 2 years following compared to patients without exacerbation.^121^ The deconditioning among patients with already compromised peripheral muscles could account for the non-improvement in 6MWD in patients with chronic obstructive pulmonary disease compared to the improvement seen in patients after acute infection with SARS-CoV, community-acquired pneumonia, and COVID-19 in our study.

Our meta-analysis showed impaired muscle strength, as measured by HGS, at 3 months to <5 months compared to predicted ranges in a healthy population of men of the same age.^122^ In a study by Gonzáles-Islas et al, 47.4% of patients with COVID-19 had low muscle strength, as measured by HGS, and 24.5% had low muscle mass, as measured by bioelectrical impedance, 3 months after hospital discharge.^48^ In addition, other studies have demonstrated that survivors of acute COVID have malnutrition,^33^^,^^123^ muscle wasting,^33^ and weight loss^124^ during acute admission, all risk factors for sarcopenia. Ribero-Baptista et al found sarcopenia to be the main contributor to reduced exercise capacity in cardiopulmonary exercise testing.^86^ Low muscular strength or low muscle mass, as well as sarcopenia, are predictors of poor outcomes such as reduced physical function, reduced ability to carry out activities of daily living, and reduced health–related quality of life. It is also associated with an increased likelihood of adverse outcomes including physical disability and mortality.^125^ Measurement of muscle strength and physical function should be considered in clinical follow-up to identify and treat those at risk of poorer outcomes.

The underlying mechanisms leading to impaired physical function following COVID-19 infection are likely multifactorial. Studies included in this review have reported more severe functional impairment in patients who had more severe acute disease^49^^,^^59^^,^^71^^,^^83^^,^^100^ such as acute respiratory distress syndrome (ARDS),^16^ and those requiring invasive ventilation.^16^^,^^48^^,^^78^^,^^85^ This supports previous knowledge that in general, prolonged admission or treatment in intensive care can lead to impairment of physical and cognitive function.^8^ In an additional subanalysis, we did not find any difference between the 6MWT in patients who were hospitalized and those who were not hospitalized, although this could be due to the small number of studies reporting patients who were not hospitalized and who were often referred or monitored for persisting impairment. People more at risk of reduced physical function were those with comorbidities such as a higher body mass index^48^^,^^71^ and frailty^33^ and the elderly,^48^ a group already at risk of reduced physical activity and a lower functional reserve. Additional research into recovered and nonrecovered phenotypes has found persistent systemic inflammation and its metabolic effects^2^^,^^58^^,^^126^ to be linked to impaired physical functioning. Identifying at-risk phenotypes and the underlying biomechanical mechanisms for this will be essential in prevention and treatment of long COVID.

Rehabilitation after chronic obstructive pulmonary disease exacerbation is common practice and has been shown to improve 6MWD and health-related quality of life and reduce hospital readmission.^127^ A 6-week exercise intervention for survivors of SARS-Cov improved patients’ 6MWD, HGS, and max$\dot{\mathsf{V}}{\mathsf{o}}_{\mathsf{2}}$ compared to controls.^128^ Early rehabilitation studies in COVID-19 have shown mixed results^129^ but more recent studies using home exercise and respiratory muscle training have been more promising^130^^,^^131^ In a meta-analysis of 3 rehabilitation studies, the pooled estimate of the effect of pulmonary rehabilitation on the 6MWD (absolute mean difference = 50.41; 95% CI = 34.34–66.48; *P* < .0001) favored the intervention group.^130^ In a single study, improvements were also seen in sit-to-stand performance and physical activity compared to controls.^132^ Given that fatigue, myalgia, and dyspnea are frequent persistent symptoms after COVID-19, they should be considered when designing a rehabilitation program for this population. Nambi et al showed that low-intensity aerobic exercise was more effective than high-intensity exercise at improving muscle strength (*P* < .001) in patients after COVID-19.^133^ In a study by Liu et al, stretching and respiratory muscle training alone resulted in improvement in 6MWD and quality of life in patients compared to controls (*P* < .05).^134^

## Limitations

Although many studies and meta-analyses have examined post-COVID-19 symptoms, the present study is—to our knowledge—the largest meta-analysis looking at functional recovery after COVID-19 infection. Comprehensive data searches across a number of databases with wide inclusion criteria identified eligible studies and authors were contacted for missing data where possible. Despite being conducted in accordance with the Preferred Reporting Items for Systematic Reviews and Meta-Analyses guidelines, there were some limitations to this study. Some outcome measures were only reported in a small number of studies which limited meta-analysis. This, in addition to variation in study population selection, contributed to heterogeneity in results. Individual patient data on age, sex assigned at birth, and body mass index were not available, and only summary statistics (means or percentages) were reported in each paper, so we were unable to further investigate the effect of these factors on our outcomes. There is then a potential bias due to uncontrolled confounders such as age, sex assigned at birth, and body mass index, as well as single site populations and heterogeneity in observational studies. Physical functioning prior to COVID-19 infection was not known in the majority of studies**.** For future research, standardizing outcome measures for functional assessment after COVID-19 would aid translative research and collaborative work.

## Conclusion

We have shown that patients have reduced physical function more than 3 months after COVID-19 infection. Decreased physical function can remain over 11 months after acute infection; however, this impairment reduces over time with a better mean physical function found after a longer recovery time. Protracted recovery supports the need to assess physical function at clinical follow-up and further research into rehabilitation programs for patients who have not recovered.

## Supplementary Material

2023-0272_R2_Forest_plots_revision_SM_pzae023

2023-0272_R2_Supplementary_tables_revision_final_pzae023

## Systematic Review Registration

This systematic review was registered with PROSPERO (CRD42022367436).
