# Supplementary material for: Functional Recovery of Adults Following Acute COVID-19: A Systematic Review and Meta-Analysis
Source: Phys Ther. 2024 Feb 22;105(1):pzae023. doi: 10.1093/ptj/pzae023 (PMC11738174; doi:10.1093/ptj/pzae023)

Forest Plots

Supplementary Figure 1: Meta-analysis of mean of 6MWT 3 months to <5 months after discharge. Black box, effect estimates from single studies; Diamond, pooled proportion with confidence interval; Weight (in %), influence an individual study had on the pooled result.

Study	Mean	SD	Population	Weight	Mean IV, Random, 95%CI
Aiello et al	404.50	77.1100	121	2.2%	404.50 [390.76; 418.24]
Anastasio et al	520.00	103.7037	379	2.2%	520.00 [509.56; 530.44]
Betschart et al	578.00	129.0000	35	2.0%	578.00 [535.26; 620.74]
Blanco et al (low_DLCO)	513.00	107.1111	52	2.1%	513.00 [483.89; 542.11]
Blanco et al(normal_DLCO)	577.00	77.7778	48	2.2%	577.00 [555.00; 599.00]
Calvo-Paniagua et al	560.10	98.9000	68	2.1%	560.10 [536.59; 583.61]
Cao et al	552.00	54.7000	40	2.2%	552.00 [535.05; 568.95]
Clavario et al	540.00	89.8519	200	2.2%	540.00 [527.55; 552.45]
Eberst et al	481.00	121.4815	79	2.1%	481.00 [454.21; 507.79]
Gianella et al	539.30	102.8000	39	2.1%	539.30 [507.04; 571.56]
Gochicoa-Rangel et al (IMV)	503.00	233.3333	85	1.9%	503.00 [453.40; 552.60]
Gochicoa-Rangel et al (no_IMV)	567.00	182.9630	86	2.0%	567.00 [528.33; 605.67]
Guler et al (mild)	576.00	78.0000	38	2.1%	576.00 [551.20; 600.80]
Guler et al (severe)	456.00	105.0000	57	2.1%	456.00 [428.74; 483.26]
Gulhan et al	538.00	57.0000	53	2.2%	538.00 [522.65; 553.35]
Jacobson et al	416.40	84.8000	118	2.2%	416.40 [401.10; 431.70]
Janssen et al (control)	437.40	124.3000	52	2.1%	437.40 [403.62; 471.18]
Janssen et al (treated)	445.60	126.6000	66	2.1%	445.60 [415.06; 476.14]
Karoli et al (mod)	440.50	16.0000	64	2.2%	440.50 [436.58; 444.42]
Karoli et al (severe)	385.60	22.0000	38	2.2%	385.60 [378.61; 392.59]
Kersten et al (hosp)	497.30	90.8000	25	2.0%	497.30 [461.71; 532.89]
Kersten et al (no_symptom)	536.00	84.0000	47	2.1%	536.00 [511.99; 560.01]
Kersten et al (symptom)	523.80	81.1000	295	2.2%	523.80 [514.55; 533.05]
Kooner et al	454.00	84.0000	76	2.2%	454.00 [435.11; 472.89]
Landi et al	535.40	86.2000	66	2.2%	535.40 [514.60; 556.20]
Larsson et al	522.00	148.8889	45	2.0%	522.00 [478.50; 565.50]
Latronico et al	420.00	100.0000	98	2.2%	420.00 [400.20; 439.80]
Marando et al	539.30	102.8000	39	2.1%	539.30 [507.04; 571.56]
Morin et al	462.00	94.0741	161	2.2%	462.00 [447.47; 476.53]
Muller et al	484.00	110.0000	210	2.2%	484.00 [469.12; 498.88]
Ribeiro Baptista et al	485.18	111.0800	105	2.2%	485.18 [463.93; 506.43]
Riou et al (critical)	435.00	73.3333	45	2.2%	435.00 [413.57; 456.43]
Riou et al (mild)	452.00	113.3333	21	1.9%	452.00 [403.53; 500.47]
Riou et al (severe)	480.00	88.8889	15	1.9%	480.00 [435.02; 524.98]
Saluja et al	540.00	2.4000	166	2.2%	540.00 [539.63; 540.37]
Shah et al	504.00	107.0000	60	2.1%	504.00 [476.93; 531.07]
Sivori et al (moderate)	458.50	92.7000	82	2.2%	458.50 [438.44; 478.56]
Sivori et al (severe)	409.50	123.2000	79	2.1%	409.50 [382.33; 436.67]
Sneller et al	560.00	78.5185	189	2.2%	560.00 [548.81; 571.19]
Sperling et al	486.90	113.2500	218	2.2%	486.90 [471.87; 501.93]
Townsend et al	480.00	88.8889	71	2.2%	480.00 [459.32; 500.68]
Uz et al	518.50	55.5600	99	2.2%	518.50 [507.56; 529.44]
Van Gassel et al	480.00	115.5556	46	2.1%	480.00 [446.61; 513.39]
Vejen et al	463.00	135.5556	129	2.1%	463.00 [439.61; 486.39]
Wahlgren et al	475.50	147.0000	108	2.1%	475.50 [447.78; 503.22]
Wu, X et al	535.00	55.5556	83	2.2%	535.00 [523.05; 546.95]
Yang et al	486.00	88.8889	92	2.2%	486.00 [467.84; 504.16]
**Total (95% CI)**			**4388**	**100.0%**	**494.57 [476.84; 512.30]**

Heterogeneity: $Tau^2 = 3676.5590$; $Chi^2 = 5960.50$, df = 46 (P = 0); $I^2 = 99\%$

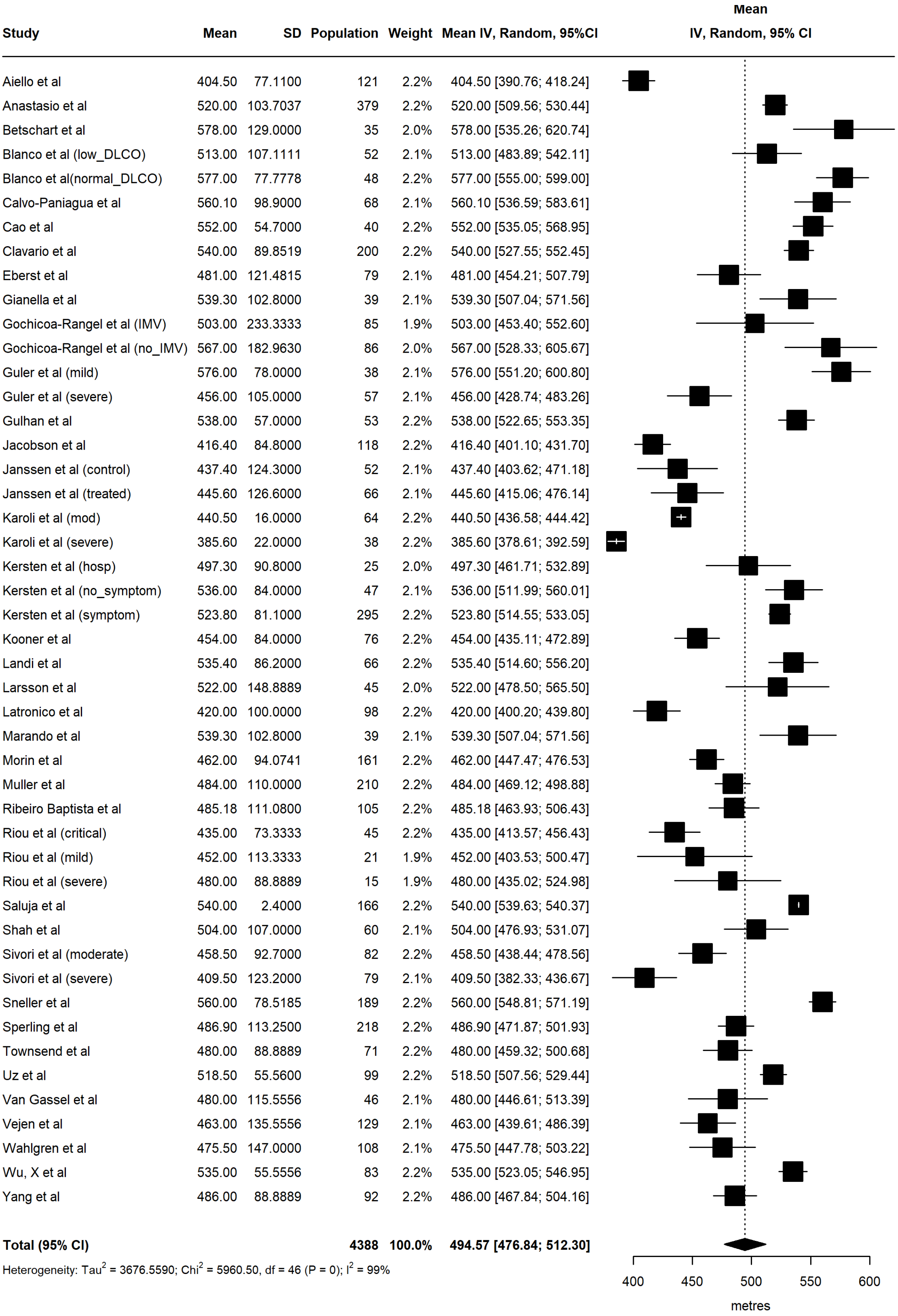

Supplementary Figure 2: Meta-analysis of mean of 6MWT 5 months to <11 months after discharge. Black box, effect estimates from single studies; Diamond, pooled proportion with confidence interval; Weight (in %), influence an individual study had on the pooled result.

Study	Mean	SD	Population	Weight	Mean IV, Random, 95%CI
Aparisi et al	558.00	85.1852	70	3.0%	558.00 [538.04; 577.96]
Aranda et al 2	377.00	117.0000	113	3.0%	377.00 [355.43; 398.57]
Bardakci et al (abnormal_CT)	492.00	117.7778	44	2.8%	492.00 [457.20; 526.80]
Bardakci et al (Normal_CT)	490.00	112.5926	21	2.5%	490.00 [441.84; 538.16]
Bretas et al (ICU)	466.60	83.6000	92	3.0%	466.60 [449.52; 483.68]
Bretas et al (Ward)	462.00	111.6000	102	3.0%	462.00 [440.34; 483.66]
Cherrez-Ojeda et al	376.90	108.4000	43	2.8%	376.90 [344.50; 409.30]
Damanti et al 2	460.00	72.2222	67	3.0%	460.00 [442.71; 477.29]
Eberst et al	549.50	94.4444	76	3.0%	549.50 [528.27; 570.73]
Faverio et al (CPAP)	473.00	72.5926	144	3.1%	473.00 [461.14; 484.86]
Faverio et al (ICU)	485.00	99.1111	97	3.0%	485.00 [465.28; 504.72]
Faverio et al (oxygen)	450.00	74.0741	71	3.0%	450.00 [432.77; 467.23]
Ferioli et al	489.00	141.0000	75	2.8%	489.00 [457.09; 520.91]
Huang, C et al	495.00	72.5926	1692	3.1%	495.00 [491.54; 498.46]
Janssen et al (control)	474.30	119.4000	43	2.8%	474.30 [438.61; 509.99]
Janssen et al (treated)	471.60	109.8000	54	2.9%	471.60 [442.31; 500.89]
Kattainen et al	548.00	75.5556	55	3.0%	548.00 [528.03; 567.97]
Latronico et al	420.00	70.3704	77	3.0%	420.00 [404.28; 435.72]
O'Brien et al	421.00	92.0000	55	2.9%	421.00 [396.69; 445.31]
Pogosova et al	480.00	59.2593	213	3.1%	480.00 [472.04; 487.96]
Riou et al (critical)	484.00	94.8148	29	2.8%	484.00 [449.49; 518.51]
Riou et al (mild)	527.00	55.5556	12	2.8%	527.00 [495.57; 558.43]
Shogenova et al (control)	397.00	30.0000	30	3.1%	397.00 [386.26; 407.74]
Shogenova et al (treatment)	430.00	45.0000	30	3.0%	430.00 [413.90; 446.10]
Sivori et al (moderate)	466.00	96.8000	56	2.9%	466.00 [440.65; 491.35]
Sivori et al (severe)	434.90	95.9000	63	2.9%	434.90 [411.22; 458.58]
Tauekelova et al (abnormal spiro)	381.00	54.5600	169	3.1%	381.00 [372.77; 389.23]
Tauekelova et al (normal spiro)	400.00	74.0741	143	3.1%	400.00 [387.86; 412.14]
Wu, X et al	585.00	54.8148	83	3.1%	585.00 [573.21; 596.79]
Yang et al	526.00	89.6296	72	3.0%	526.00 [505.30; 546.70]
Yildirim et al (ICU)	445.00	125.1852	31	2.6%	445.00 [400.93; 489.07]
Yildirim et al (non_ICU)	461.00	101.4815	39	2.8%	461.00 [429.15; 492.85]
Zhang et al	500.00	66.6667	280	3.1%	500.00 [492.19; 507.81]
Zhou et al	514.10	73.0000	120	3.1%	514.10 [501.04; 527.16]
**Total (95% CI)**			**4361**	**100.0%**	**470.25 [452.48; 488.03]**

Heterogeneity: Tau^2 = 2649.3397; Chi^2 = 1827.72, df = 33 (P = 0); I^2 = 98%

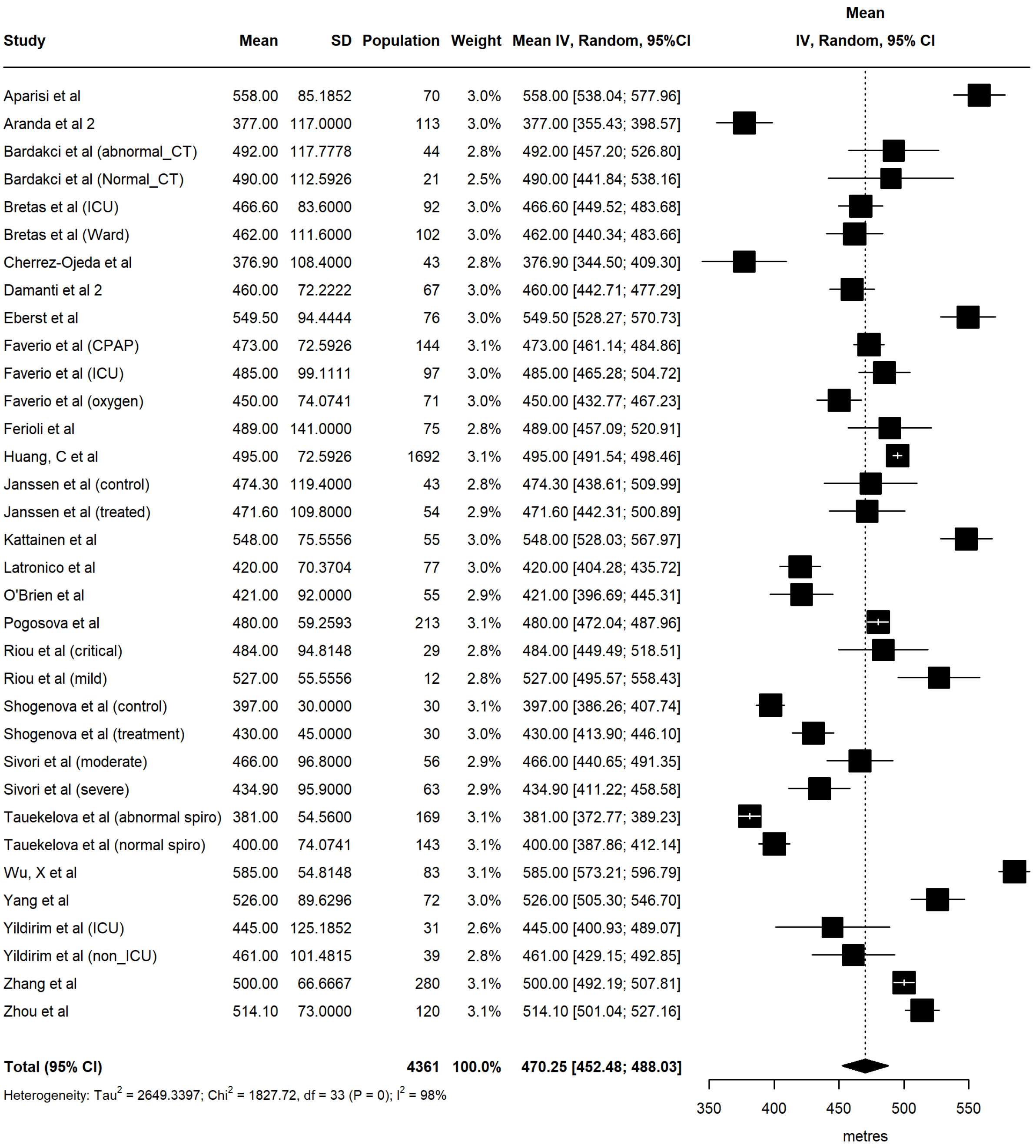

Supplementary Figure 3: Meta-analysis of mean of 6MWT ≥11 months after discharge. Black box, effect estimates from single studies; Diamond, pooled proportion with confidence interval; Weight (in %), influence an individual study had on the pooled result.

Study	Mean	SD	Population	Weight	Mean IV, Random, 95%CI
Aranda et al	419.00	136.0000	146	5.3%	419.00 [396.94; 441.06]
Betschart et al	563.00	124.0000	36	4.8%	563.00 [522.49; 603.51]
Eberst et al	542.00	77.7778	66	5.3%	542.00 [523.24; 560.76]
Faverio et al 2 (CPAP)	460.00	81.4815	136	5.4%	460.00 [446.31; 473.69]
Faverio et al 2 (ICU)	475.00	92.5926	90	5.3%	475.00 [455.87; 494.13]
Faverio et al 2 (oxygen)	470.00	83.7037	61	5.3%	470.00 [448.99; 491.01]
Huang, L et al	495.00	74.8148	1248	5.5%	495.00 [490.85; 499.15]
Larsson et al	525.00	132.5926	45	4.8%	525.00 [486.26; 563.74]
Latronico et al	420.00	44.4444	51	5.4%	420.00 [407.80; 432.20]
Liao et al	555.00	55.5556	303	5.5%	555.00 [548.74; 561.26]
Lorent et al	606.00	111.8519	222	5.4%	606.00 [591.29; 620.71]
Marando et al	556.40	92.1000	38	5.1%	556.40 [527.12; 585.68]
Martino et al (low_DLCO)	461.50	115.5556	28	4.7%	461.50 [418.70; 504.30]
Martino et al (normal_DLCO)	517.50	88.8889	31	5.1%	517.50 [486.21; 548.79]
O'Brien et al	447.00	85.0000	55	5.3%	447.00 [424.54; 469.46]
Wu, X et al	615.00	37.0370	83	5.5%	615.00 [607.03; 622.97]
Yang et al	533.00	82.2222	66	5.3%	533.00 [513.16; 552.84]
Zhang et al	505.00	74.0741	247	5.5%	505.00 [495.76; 514.24]
Zhao et al	504.00	39.7333	94	5.5%	504.00 [495.97; 512.03]
**Total (95% CI)**			**3046**	**100.0%**	**508.94 [484.28; 533.59]**

Heterogeneity: $Tau^2 = 2873.3999$; $Chi^2 = 1476.02$, df = 18 ($P < 0.01$); $I^2 = 99\%$

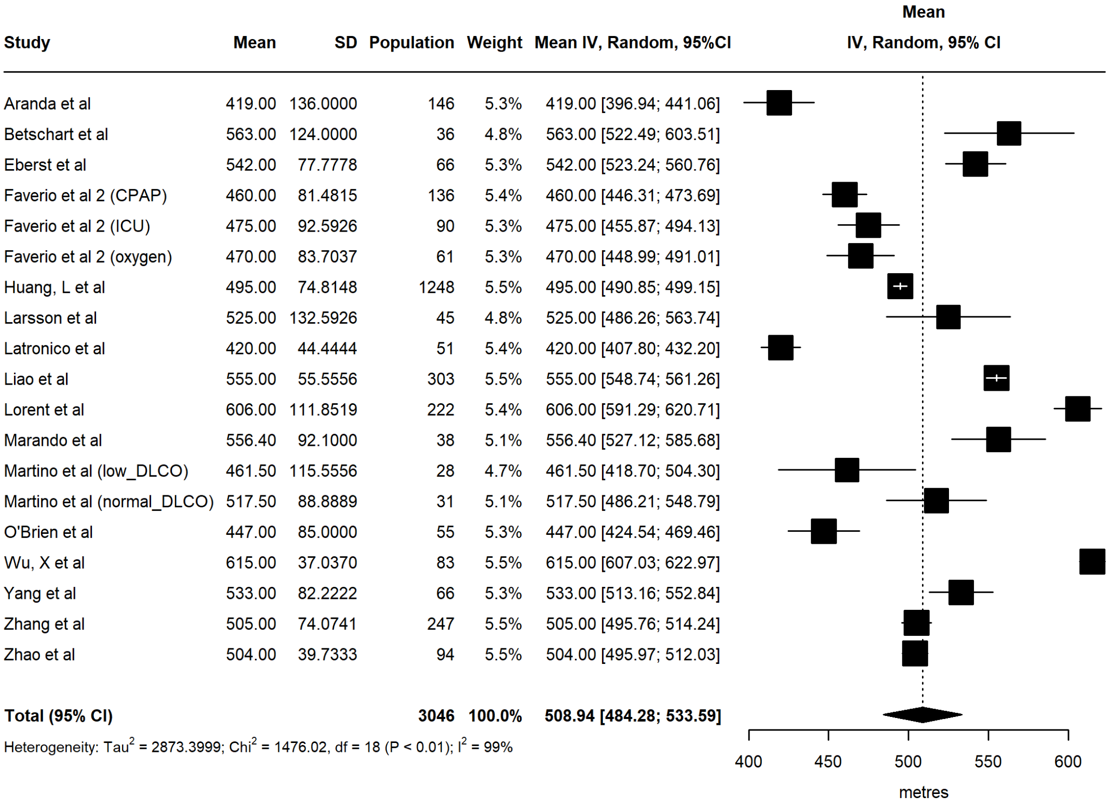

Supplementary Figure 4: Meta-analysis of mean of percentage predicted 6MWT 3 months to <5 months after discharge. Black box, effect estimates from single studies; Diamond, pooled proportion with confidence interval; Weight (in %), influence an individual study had on the pooled result.

Study	Mean	SD	Population	Weight	Mean IV, Random, 95%CI
Aiello et al	103.90	17.1000	121	4.8%	103.90 [100.85; 106.95]
Betschart et al	102.00	21.0000	35	4.5%	102.00 [95.04; 108.96]
Gochicoa-Rangel et al (no_IMV)	68.80	21.4815	86	4.7%	68.80 [64.26; 73.34]
Gochicoa-Rangel et al (IMV)	64.80	29.6296	85	4.6%	64.80 [58.50; 71.10]
Gonzalez et al	83.70	26.0000	93	4.7%	83.70 [78.42; 88.98]
Jacobson et al	59.20	11.7000	118	4.9%	59.20 [57.09; 61.31]
Janssen et al (control)	74.60	18.3000	52	4.7%	74.60 [69.63; 79.57]
Janssen et al (treated)	71.40	19.0000	66	4.7%	71.40 [66.82; 75.98]
Karoli et al (mod)	94.00	8.0000	64	4.9%	94.00 [92.04; 95.96]
Karoli et al (severe)	80.00	5.0000	38	4.9%	80.00 [78.41; 81.59]
Latronico et al	80.00	22.2222	98	4.8%	80.00 [75.60; 84.40]
Marando et al	99.00	13.6000	39	4.8%	99.00 [94.73; 103.27]
Muller et al	79.10	13.0000	210	4.9%	79.10 [77.34; 80.86]
Noel-Savina et al	92.60	21.4815	72	4.7%	92.60 [87.64; 97.56]
Sivori et al (moderate)	91.50	21.4000	82	4.7%	91.50 [86.87; 96.13]
Sivori et al (severe)	78.80	21.7000	79	4.7%	78.80 [74.01; 83.59]
van den Borst et al	92.00	18.0000	115	4.8%	92.00 [88.71; 95.29]
Van Gassel et al	80.80	22.3704	46	4.6%	80.80 [74.34; 87.26]
Yang et al	89.00	13.3333	92	4.9%	89.00 [86.28; 91.72]
Anastasio et al	88.00	16.2963	379	4.9%	88.00 [86.36; 89.64]
Shah et al	96.00	16.0000	60	4.8%	96.00 [91.95; 100.05]
**Total (95% CI)**			**2030**	**100.0%**	**84.25 [79.21; 89.30]**

Heterogeneity: $Tau^2 = 134.4007$; $Chi^2 = 1163.17$, df = 20 ($P < 0.01$); $I^2 = 98\%$

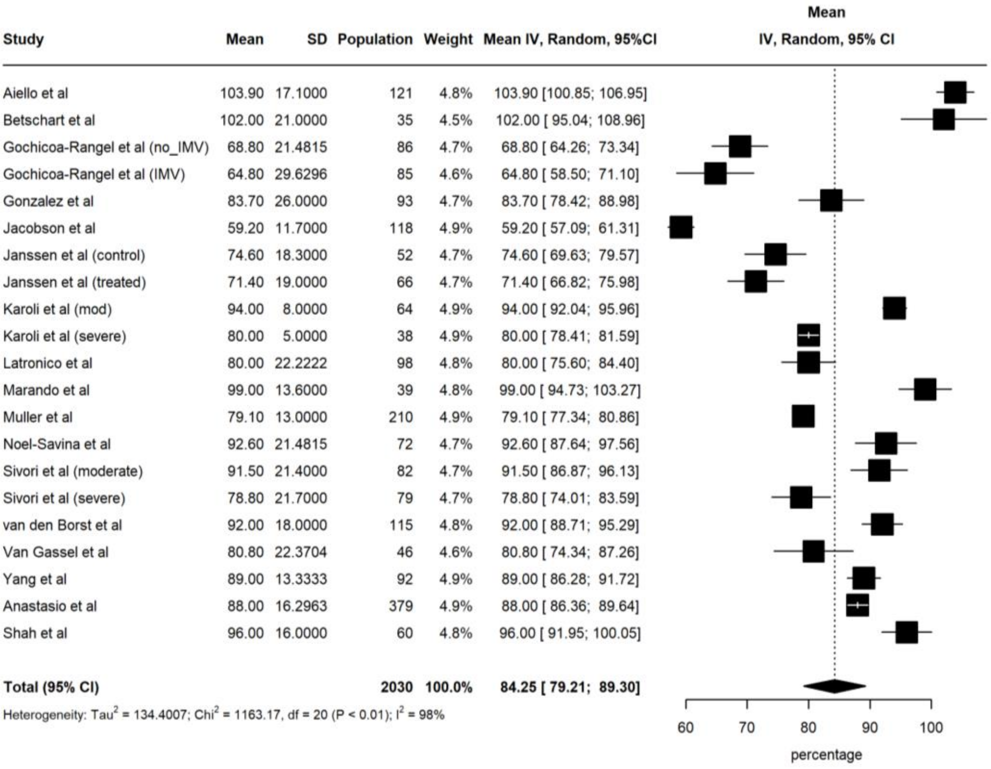

Supplementary Figure 5: Meta-analysis of mean percentage predicted 6MWT 5 months to <11 months after discharge. Black box, effect estimates from single studies; Diamond, pooled proportion with confidence interval; Weight (in %), influence an individual study had on the pooled result.

Study	Mean	SD	Population	Weight	Mean IV, Random, 95%CI
Bretas et al (Ward)	90.30	18.8000	102	5.9%	90.30 [86.65; 93.95]
Bretas et al (ICU)	90.00	16.3000	92	5.9%	90.00 [86.67; 93.33]
Cherrez-Ojeda et al	69.00	17.8000	43	5.7%	69.00 [63.68; 74.32]
Damanti et al 2	92.00	11.1111	67	6.0%	92.00 [89.34; 94.66]
Ferioli et al	73.00	22.0000	75	5.8%	73.00 [68.02; 77.98]
Gonzalez et al	91.40	19.9000	77	5.8%	91.40 [86.96; 95.84]
Janssen et al (control)	75.60	18.6000	43	5.7%	75.60 [70.04; 81.16]
Janssen et al (treated)	76.20	16.5000	54	5.8%	76.20 [71.80; 80.60]
Kattainen et al	107.70	15.1111	55	5.9%	107.70 [103.71; 111.69]
Latronico et al	70.00	7.4074	77	6.0%	70.00 [68.35; 71.65]
Schandl et al	88.00	18.5185	59	5.8%	88.00 [83.27; 92.73]
Sivori et al (moderate)	91.00	19.2000	56	5.8%	91.00 [85.97; 96.03]
Sivori et al (severe)	85.10	17.3000	63	5.8%	85.10 [80.83; 89.37]
Staudt et al	101.90	16.8148	99	5.9%	101.90 [98.59; 105.21]
Yang et al	97.00	12.5926	72	6.0%	97.00 [94.09; 99.91]
Zhang et al	87.90	13.9259	280	6.0%	87.90 [86.27; 89.53]
Zhou et al	92.40	12.5926	120	6.0%	92.40 [90.15; 94.65]
**Total (95% CI)**			**1434**	**100.0%**	**87.04 [81.71; 92.37]**

Heterogeneity: $Tau^2 = 121.6756$; $Chi^2 = 790.78$, df = 16 ($P < 0.01$); $I^2 = 98\%$

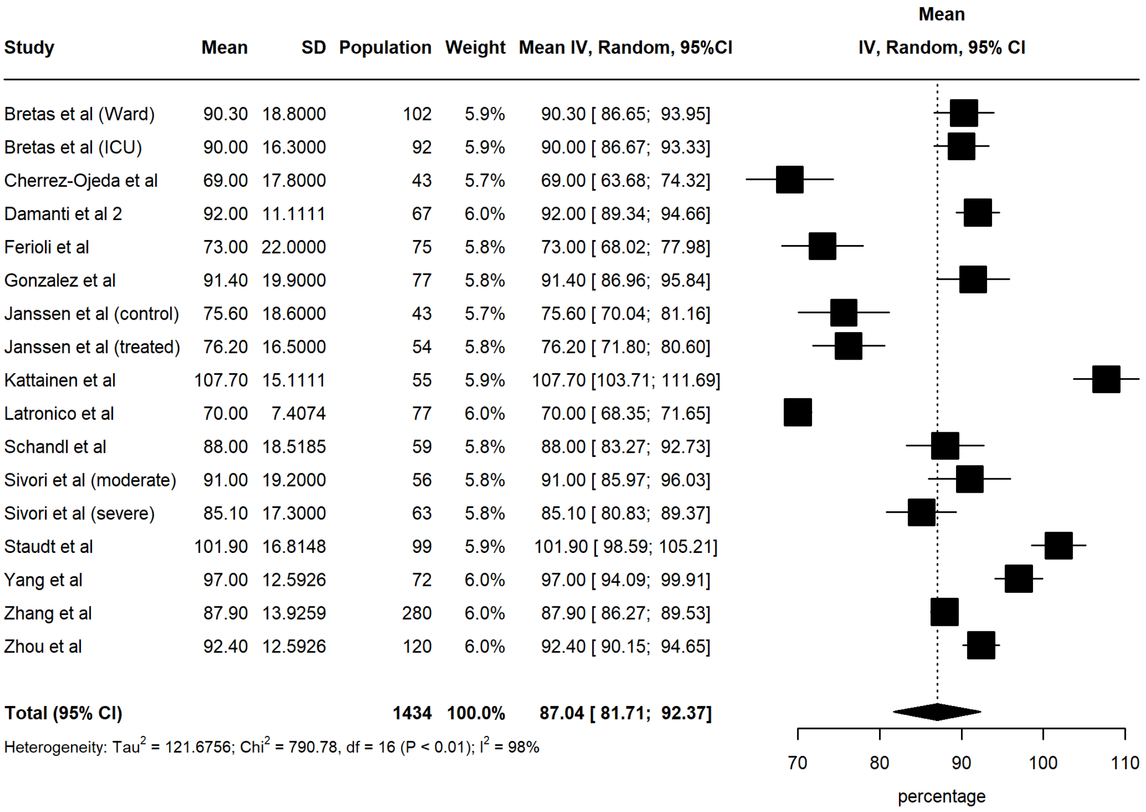

Supplementary Figure 6: Meta-analysis of mean of percentage predicted 6MWT ≥11 months after discharge. Black box, effect estimates from single studies; Diamond, pooled proportion with confidence interval; Weight (in %), influence an individual study had on the pooled result.

Study	Mean	SD	Population	Weight	Mean IV, Random, 95%CI
Betschart et al	100.00	22.0000	36	7.2%	100.00 [92.81; 107.19]
Gonzalez et al	95.30	21.4000	37	7.5%	95.30 [88.40; 102.20]
Huang, L et al	90.00	12.8148	1248	13.8%	90.00 [89.29; 90.71]
Latronico et al	80.00	22.2222	51	8.3%	80.00 [73.90; 86.10]
Liao et al	87.00	9.6296	303	13.7%	87.00 [85.92; 88.08]
Lorent et al	89.00	12.5926	222	13.3%	89.00 [87.34; 90.66]
Marando et al	104.60	12.7000	38	10.7%	104.60 [100.56; 108.64]
Yang et al	99.00	11.1111	66	12.3%	99.00 [96.32; 101.68]
Zhang et al	90.80	13.7037	247	13.2%	90.80 [89.09; 92.51]
**Total (95% CI)**			**2248**	**100.0%**	**92.52 [89.76; 95.28]**

Heterogeneity: $Tau^2 = 14.1963$; $Chi^2 = 145.00$, df = 8 ($P < 0.01$); $I^2 = 94\%$

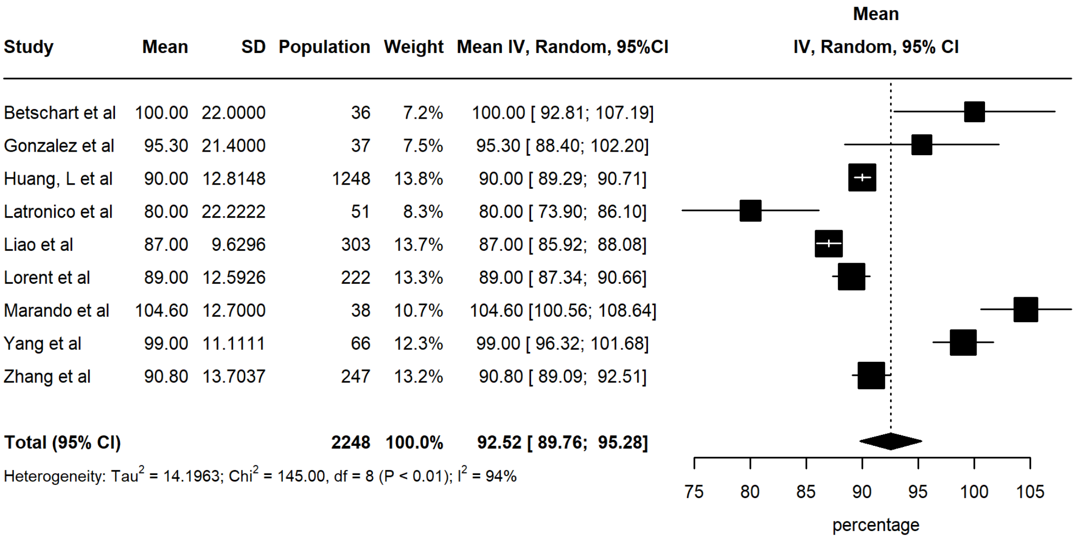

Supplementary Figure 7: Meta-analysis of mean Handgrip strength 3 months to <5 months after discharge. Black box, effect estimates from single studies; Diamond, pooled proportion with confidence interval; Weight (in %), influence an individual study had on the pooled result.

Study	Mean	SD	Population	Weight	Mean IV, Random, 95%CI
Damanti et al (frail)	19.80	8.9630	35	8.3%	19.80 [16.83; 22.77]
Damanti et al (robust)	21.70	10.5926	181	8.8%	21.70 [20.16; 23.24]
Del Corral et al	32.40	11.8000	102	8.6%	32.40 [30.11; 34.69]
González-Islas et al	23.60	9.3200	530	9.0%	23.60 [22.81; 24.39]
Landi et al	30.80	10.9000	66	8.4%	30.80 [28.17; 33.43]
Latronico et al	29.00	8.1481	98	8.8%	29.00 [27.39; 30.61]
Levy et al	28.00	38.5185	139	6.4%	28.00 [21.60; 34.40]
Mittal et al	27.86	10.4700	52	8.3%	27.86 [25.01; 30.71]
Ribeiro Baptista et al	34.42	10.3700	105	8.7%	34.42 [32.44; 36.40]
Rousseau et al	28.00	11.8519	32	7.7%	28.00 [23.89; 32.11]
Van Gassel et al	29.40	9.3000	46	8.4%	29.40 [26.71; 32.09]
Muller et al	36.40	14.7000	147	8.5%	36.40 [34.02; 38.78]
**Total (95% CI)**			**1533**	**100.0%**	**28.45 [25.46; 31.45]**

Heterogeneity: $Tau^2 = 25.8916$; $Chi^2 = 286.98$, df = 11 ($P < 0.01$); $I^2 = 96\%$

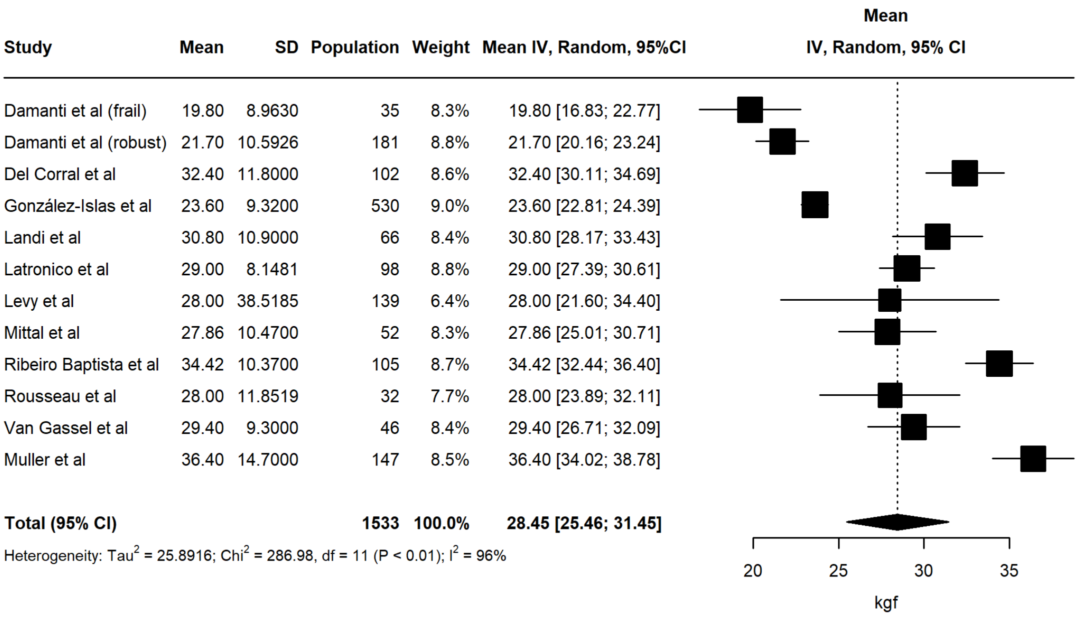

Supplementary Figure 8: Meta-analysis of mean Handgrip strength 5 months to <11 months after discharge. Black box, effect estimates from single studies; Diamond, pooled proportion with confidence interval; Weight (in %), influence an individual study had on the pooled results.

Study	Mean	SD	Population	Weight	Mean IV, Random, 95%CI
Damanti et al (frail)	22.40	6.8889	28	12.7%	22.40 [19.85; 24.95]
Damanti et al (robust)	22.20	9.1111	148	13.3%	22.20 [20.73; 23.67]
de Sousa et al	26.70	10.3704	40	12.3%	26.70 [23.49; 29.91]
Del Brutto et al	22.70	7.9000	149	13.3%	22.70 [21.43; 23.97]
Del Corral et al	33.70	11.9000	102	12.9%	33.70 [31.39; 36.01]
Jimeno-Almazan et al (a)	34.50	9.9000	20	11.5%	34.50 [30.16; 38.84]
Jimeno-Almazan et al (b)	35.70	9.7000	19	11.5%	35.70 [31.34; 40.06]
Latronico et al	32.00	12.5926	77	12.6%	32.00 [29.19; 34.81]
**Total (95% CI)**			**583**	**100.0%**	**28.52 [24.74; 32.29]**

Heterogeneity: $Tau^2 = 27.4647$; $Chi^2 = 150.68$, df = 7 ($P < 0.01$); $I^2 = 95\%$

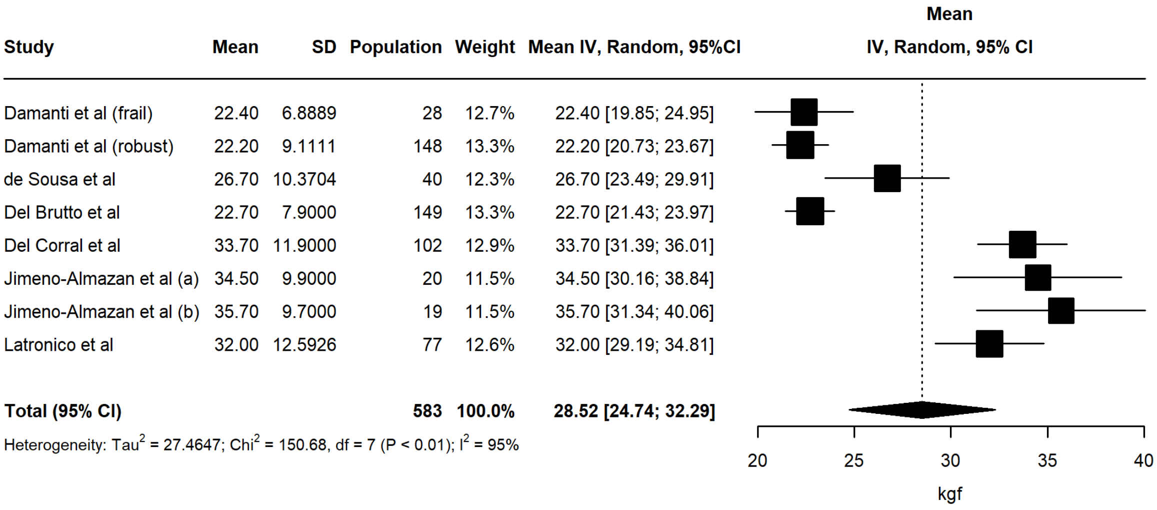

Supplementary Figure 9: Meta-analysis of mean handgrip strength ≥11 months after discharge. Black box, effect estimates from single studies; Diamond, pooled proportion with confidence interval; Weight (in %), influence an individual study had on the pooled result.

Study	Mean	SD	Population	Weight	Mean IV, Random, 95%CI
Latronico et al	37.00	10.3704	51	49.2%	37.00 [34.15; 39.85]
Xiong et al 2	25.50	6.8889	446	50.8%	25.50 [24.86; 26.14]
**Total (95% CI)**			**497**	**100.0%**	**31.16 [19.89; 42.43]**

Heterogeneity: $Tau^2 = 65.0174$; $Chi^2 = 59.70$, df = 1 ($P < 0.01$); $I^2 = 98\%$

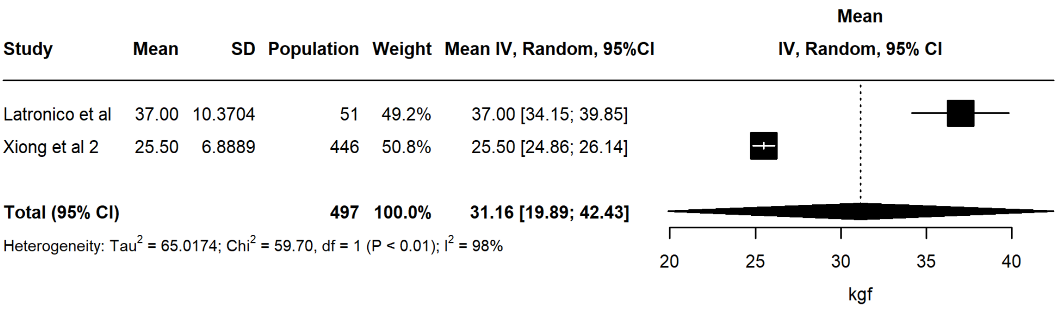

Supplementary Figure 10: Meta-analysis of mean 1 minute sit to stand 3 months to <5 months after discharge. Black box, effect estimates from single studies; Diamond, pooled proportion with confidence interval; Weight (in %), influence an individual study had on the pooled result.

Study	Mean	SD	Population	Weight	Mean IV, Random, 95%CI
Clavario et al	10.50	2.8889	200	33.5%	10.50 [10.10; 10.90]
Del Corral et al	31.70	9.7000	102	33.3%	31.70 [29.82; 33.58]
Landi et al	27.10	10.5000	66	33.2%	27.10 [24.57; 29.63]
**Total (95% CI)**			**368**	**100.0%**	**23.08 [7.43; 38.72]**

Heterogeneity: $Tau^2 = 190.2626$; $Chi^2 = 609.93$, df = 2 ($P < 0.01$); $I^2 = 100\%$

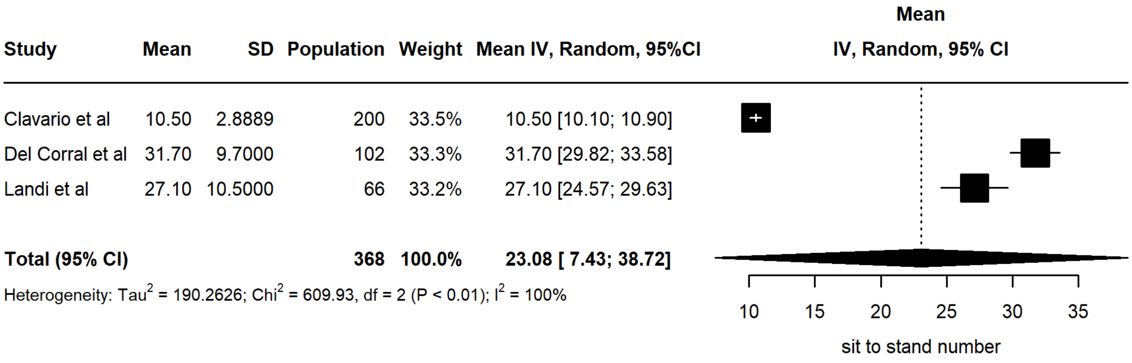

Supplementary Figure 11: Meta-analysis of mean 1 minute sit to stand 5 months to <11 months after discharge. Black box, effect estimates from single studies; Diamond, pooled proportion with confidence interval; Weight (in %), influence an individual study had on the pooled result.

Study	Mean	SD	Population	Weight	Mean IV, Random, 95%CI
Del Corral et al	34.70	11.9000	102	49.7%	34.70 [32.39; 37.01]
Sevillano-Castano et al	22.00	4.6667	42	50.3%	22.00 [20.59; 23.41]
**Total (95% CI)**			**144**	**100.0%**	**28.32 [15.87; 40.76]**

Heterogeneity: $Tau^2 = 79.6916$; $Chi^2 = 84.58$, df = 1 ($P < 0.01$); $I^2 = 99\%$

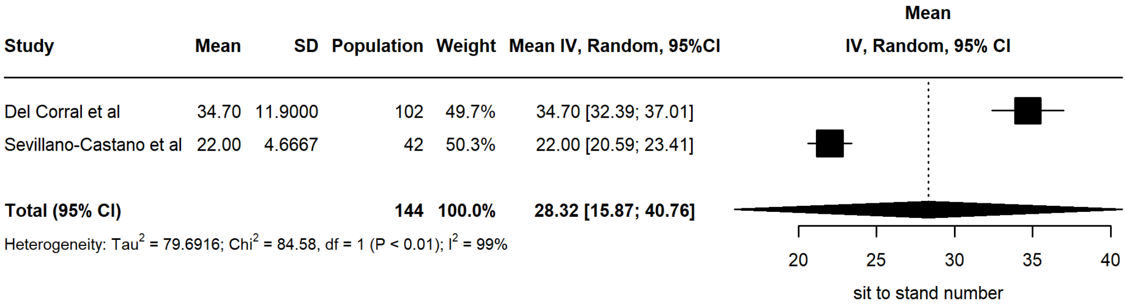

Supplementary Figure 12: Meta-analysis of mean SPPB 3 months to <5 months after discharge. Black box, effect estimates from single studies; Diamond, pooled proportion with confidence interval; Weight (in %), influence an individual study had on the pooled result.

Study	Mean	SD	Population	Weight	Mean IV, Random, 95%CI
Damanti et al (frail)	11.00	2.2222	35	10.6%	11.00 [10.26; 11.74]
Damanti et al (robust)	12.00	0.7407	181	49.5%	12.00 [11.89; 12.11]
Muller et al	12.00	1.3333	147	39.9%	12.00 [11.78; 12.22]
**Total (95% CI)**			**363**	**100.0%**	**11.89 [11.63; 12.16]**

Heterogeneity: $Tau^2 = 0.0345$; $Chi^2 = 6.97$, df = 2 (P = 0.03); $I^2 = 71\%$

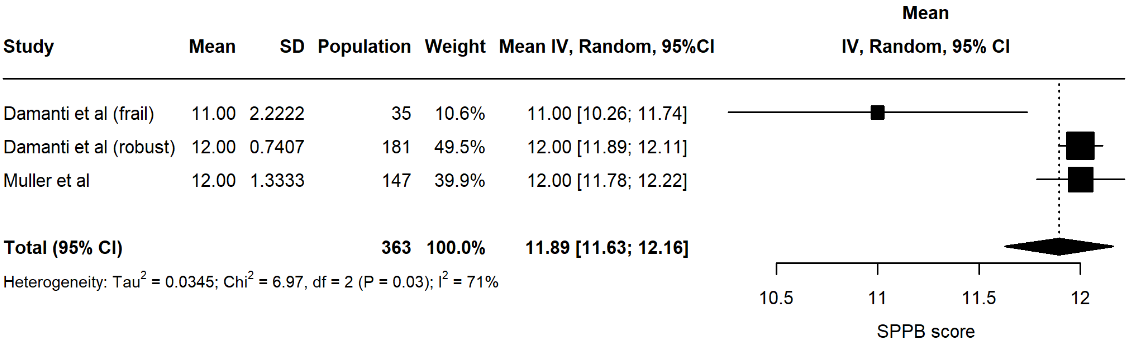

Supplementary Figure 13: Meta-analysis of mean SPPB 5 months to <11 months after discharge. Black box, effect estimates from single studies; Diamond, pooled proportion with confidence interval; Weight (in %), influence an individual study had on the pooled result.

Study	Mean	SD	Population	Weight	Mean IV, Random, 95%CI
Damanti et al (frail)	11.00	1.4815	28	32.5%	11.00 [10.45; 11.55]
Damanti et al (robust)	12.00	0.7407	148	33.7%	12.00 [11.88; 12.12]
Evans et al 1	9.90	2.4000	970	33.7%	9.90 [9.75; 10.05]
**Total (95% CI)**			**1146**	**100.0%**	**10.97 [9.35; 12.58]**

Heterogeneity: $Tau^2 = 2.0138$; $Chi^2 = 457.67$, df = 2 (P < 0.01); $I^2 = 100\%$

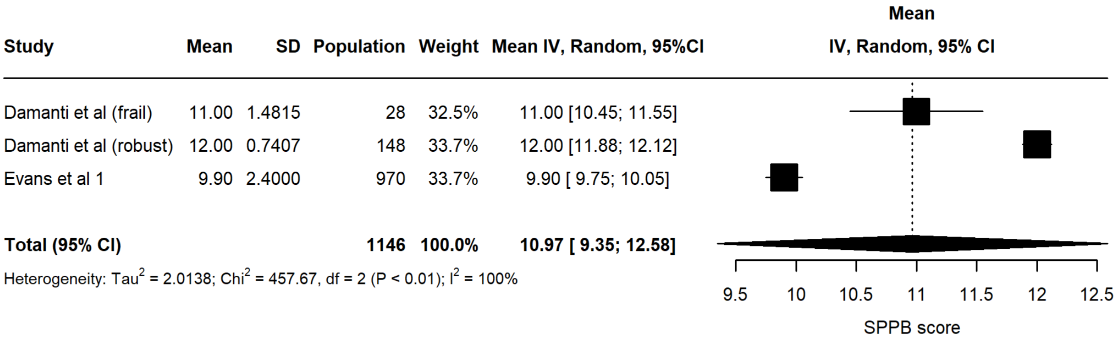

Supplementary Figure 14: Meta-analysis of mean peak VO2 (ml/min/Kg) from cycle CPET at 3 months to <5 months after discharge. Black box, effect estimates from single studies; Diamond, pooled proportion with confidence interval; Weight (in %), influence an individual study had on the pooled result.

Study	Mean	SD	Population	Weight	Mean IV, Random, 95%CI
Clavario et al	20.10	5.8519	200	16.0%	20.10 [19.29; 20.91]
Evers et al	24.00	7.0000	30	12.5%	24.00 [21.50; 26.50]
Johnsen et al	27.50	8.9000	31	11.0%	27.50 [24.37; 30.63]
Joris et al	24.80	6.1481	31	13.4%	24.80 [22.64; 26.96]
Motiejunaite et al	17.90	4.3704	114	16.0%	17.90 [17.10; 18.70]
Ribeiro Baptista et al	18.28	5.3400	105	15.7%	18.28 [17.26; 19.30]
Rinaldo et al	20.00	5.5000	75	15.3%	20.00 [18.76; 21.24]
**Total (95% CI)**			**586**	**100.0%**	**21.38 [19.58; 23.18]**

Heterogeneity: $Tau^2 = 5.1213$; $Chi^2 = 83.92$, df = 6 (P < 0.01); $I^2 = 93\%$

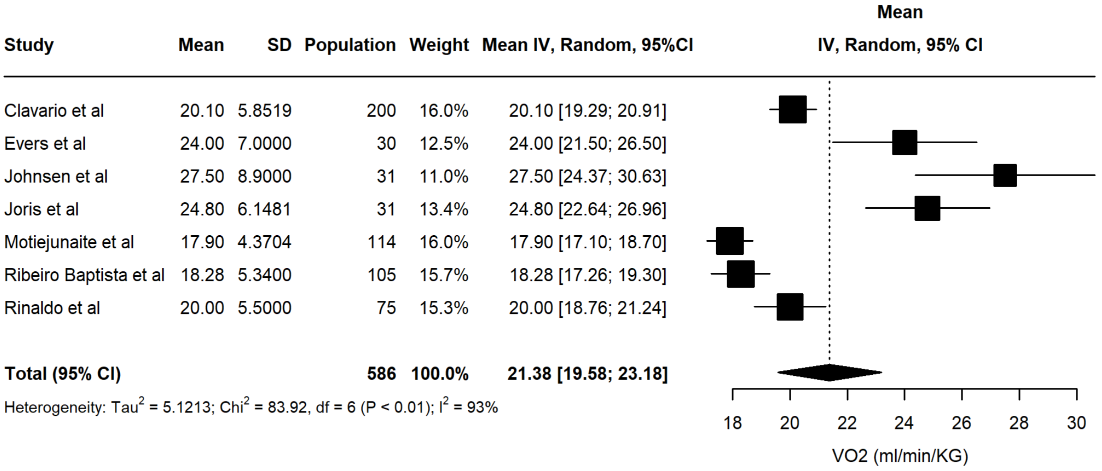

Supplementary Figure 15: Meta-analysis of mean peak VO2 (ml/min/Kg) from cycle CPET at 5 months to <11 months after discharge. Black box, effect estimates from single studies; Diamond, pooled proportion with confidence interval; Weight (in %), influence an individual study had on the pooled result

Study	Mean	SD	Population	Weight	Mean IV, Random, 95%CI
Cassar et al	20.50	6.3704	42	17.2%	20.50 [18.57; 22.43]
Evers et al	23.00	7.0000	16	16.5%	23.00 [19.57; 26.43]
Jimeno-Almazan et al (control)	36.40	10.1000	20	16.0%	36.40 [31.97; 40.83]
Jimeno-Almazan et al (exercise)	36.80	10.2000	19	15.9%	36.80 [32.21; 41.39]
Ladlow et al	34.40	7.2000	34	17.0%	34.40 [31.98; 36.82]
Ladlow et al 2	34.50	7.4000	205	17.4%	34.50 [33.49; 35.51]
**Total (95% CI)**			**336**	**100.0%**	**30.84 [24.83; 36.85]**

Heterogeneity: $Tau^2 = 53.7851$; $Chi^2 = 197.73$, df = 5 ($P < 0.01$); $I^2 = 97\%$

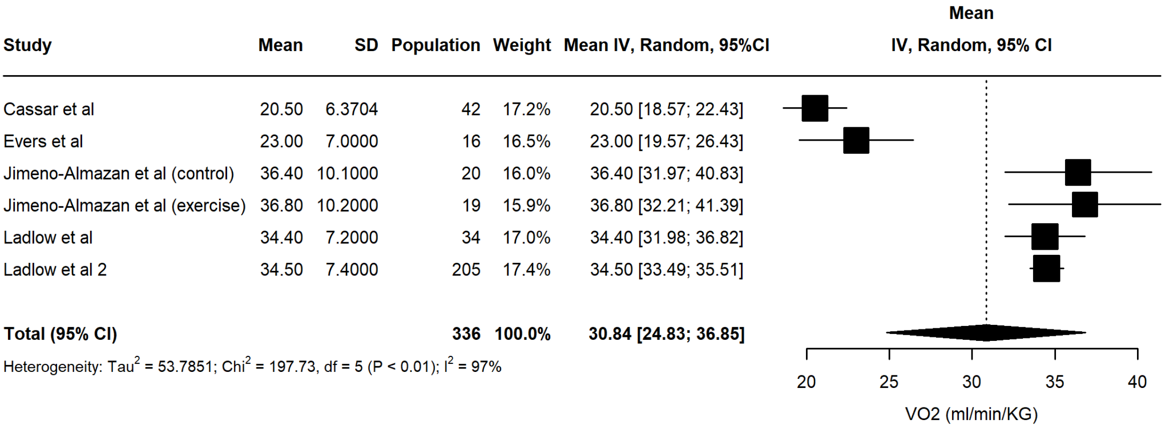

Supplementary Figure 16: Meta-analysis of mean % predicted peak VO2 from cycle CPET at 3 months to <5 months after discharge. Black box, effect estimates from single studies; Diamond, pooled proportion with confidence interval; Weight (in %), influence an individual study had on the pooled result.

Study	Mean	SD	Population	Weight	Mean IV, Random, 95%CI
Abdallah et al (H)	64.30	19.2000	25	15.2%	64.30 [56.77; 71.83]
Abdallah et al (NH)	83.50	17.9000	38	16.7%	83.50 [77.81; 89.19]
Clavario et al	85.00	17.9259	200	18.8%	85.00 [82.52; 87.48]
Joris et al	74.50	29.7778	31	12.7%	74.50 [64.02; 84.98]
Motiejunaite et al	71.00	18.5185	114	18.3%	71.00 [67.60; 74.40]
Rinaldo et al	83.00	15.0000	75	18.3%	83.00 [79.61; 86.39]
**Total (95% CI)**			**483**	**100.0%**	**77.34 [70.98; 83.71]**

Heterogeneity: $Tau^2 = 54.5890$; $Chi^2 = 64.66$, df = 5 ($P < 0.01$); $I^2 = 92\%$

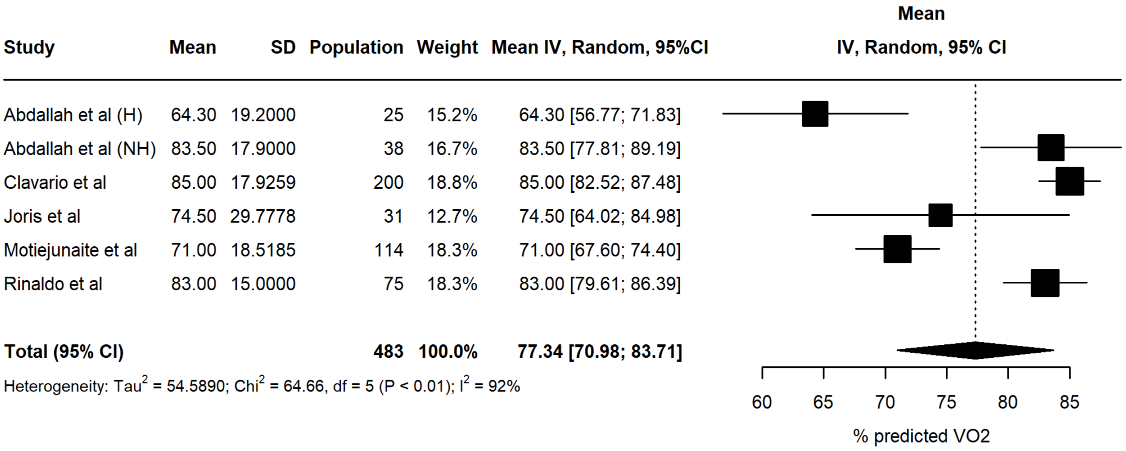

Supplementary Figure 17: Meta-analysis of mean % predicted peak VO2 from cycle CPET at 5 months to <11 months after discharge. Black box, effect estimates from single studies; Diamond, pooled proportion with confidence interval; Weight (in %), influence an individual study had on the pooled result.

Study	Mean	SD	Population	Weight	Mean IV, Random, 95%CI
Ladlow et al	111.00	19.0000	34	49.8%	111.00 [104.61; 117.39]
Vannini et al	81.00	16.0000	41	50.2%	81.00 [76.10; 85.90]
**Total (95% CI)**			**75**	**100.0%**	**95.93 [66.53; 125.33]**

Heterogeneity: $Tau^2 = 441.5692$; $Chi^2 = 53.38$, df = 1 ($P < 0.01$); $I^2 = 98\%$

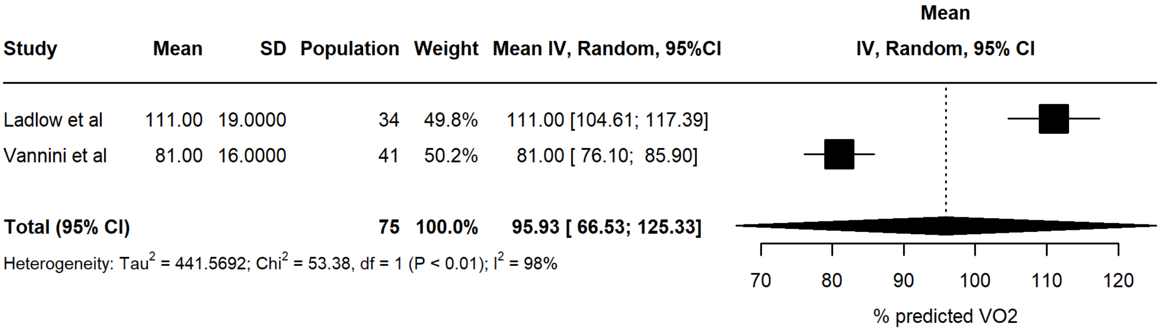

Supplementary Figure 18: Meta-analysis of mean % predicted peak VO2 from cycle CPET at ≥11 months after discharge. Black box, effect estimates from single studies; Diamond, pooled proportion with confidence interval; Weight (in %), influence an individual study had on the pooled result.

Study	Mean	SD	Population	Weight	Mean IV, Random, 95%CI
Eberst et al	99.00	13.3333	61	57.4%	99.00 [95.65; 102.35]
Steinbeis et al	90.48	27.0900	54	42.6%	90.48 [83.25; 97.71]
**Total (95% CI)**			**115**	**100.0%**	**95.37 [87.11; 103.63]**

Heterogeneity: $Tau^2 = 28.0429$; $Chi^2 = 4.40$, df = 1 (P = 0.04); $I^2 = 77\%$

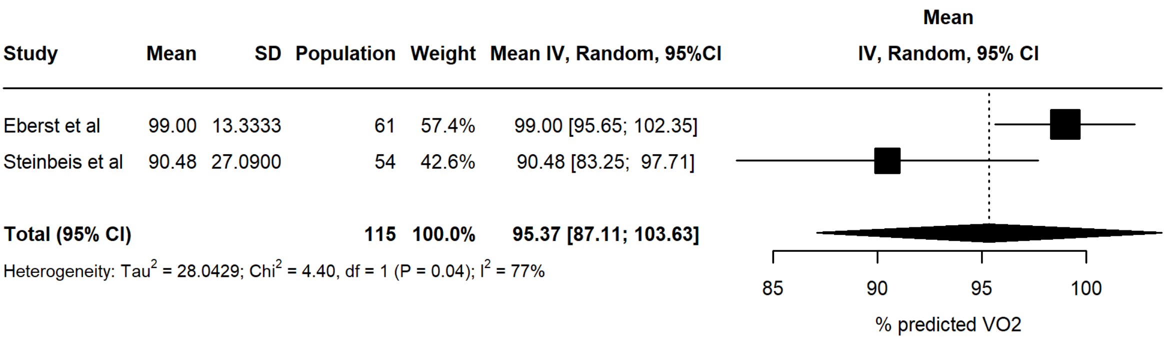

Supplementary Figure 19: Meta-analysis of mean peak VO2 (ml/min/Kg) from treadmill CPET at 5 months to <11 months after discharge. Black box, effect estimates from single studies; Diamond, pooled proportion with confidence interval; Weight (in %), influence an individual study had on the pooled result.

Study	Mean	SD	Population	Weight	Mean IV, Random, 95%CI
Aparisi et al	19.40	5.6296	70	25.3%	19.40 [18.08; 20.72]
Longobardi et al	21.58	5.2519	30	24.9%	21.58 [19.70; 23.46]
Margalit et al (N)	30.70	7.5200	63	25.0%	30.70 [28.84; 32.56]
Margalit et al (Fatigue)	27.69	7.5200	50	24.8%	27.69 [25.61; 29.77]
**Total (95% CI)**			**213**	**100.0%**	**24.82 [19.41; 30.22]**

Heterogeneity: $Tau^2 = 29.5535$; $Chi^2 = 114.35$, df = 3 (P < 0.01); $I^2 = 97\%$

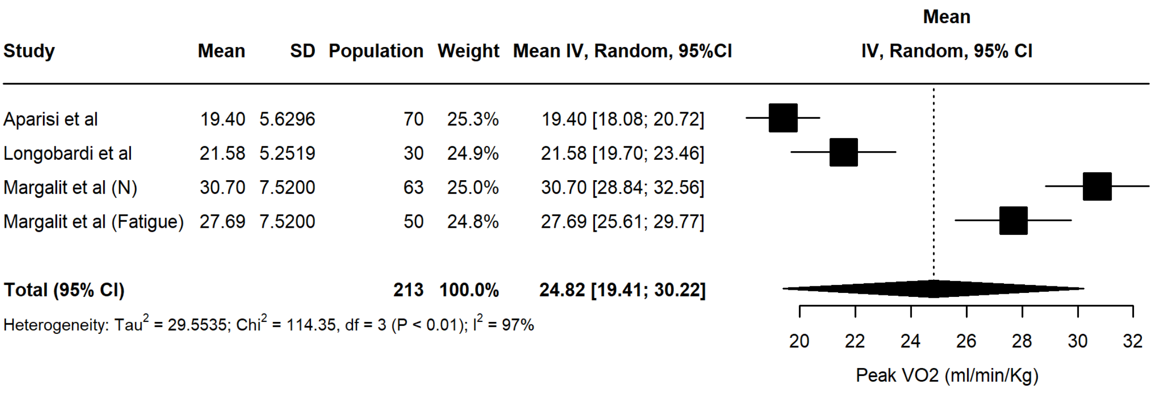

Supplementary Figure 20: Meta-analysis of mean percentage predicted peak VO2 from treadmill CPET at 5 months to <11 months after discharge. Black box, effect estimates from single studies; Diamond, pooled proportion with confidence interval; Weight (in %), influence an individual study had on the pooled result.

Study	Mean	SD	Population	Weight	Mean IV, Random, 95%CI
Aparisi et al	88.00	17.7778	70	25.4%	88.00 [83.84; 92.16]
Longobardi et al	72.10	16.9630	30	24.3%	72.10 [66.03; 78.17]
Margalit et al (N)	99.60	17.4000	63	25.3%	99.60 [95.30; 103.90]
Margalit et al (Fatigue)	96.07	18.3300	50	24.9%	96.07 [90.99; 101.15]
**Total (95% CI)**			**213**	**100.0%**	**89.08 [78.46; 99.70]**

Heterogeneity: $Tau^2 = 111.1030$; $Chi^2 = 58.44$, df = 3 (P < 0.01); $I^2 = 95\%$

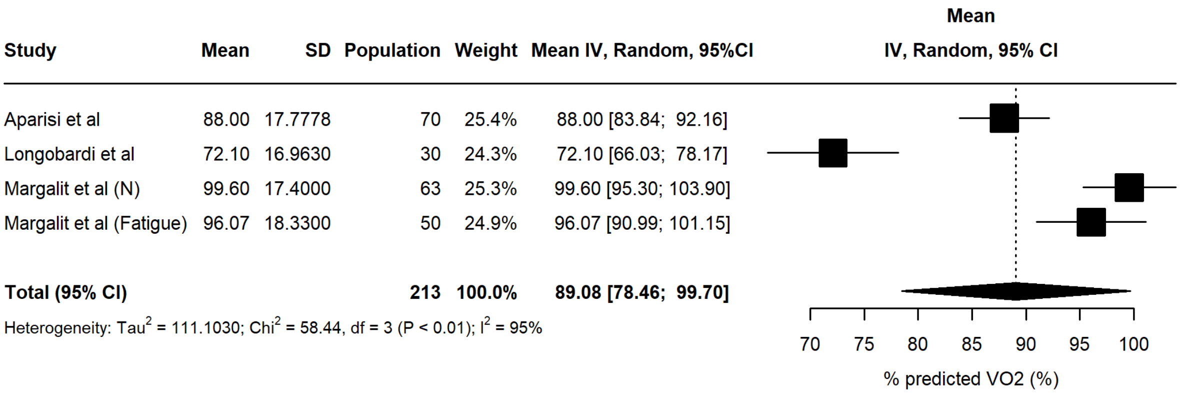

Supplementary Figure 21: Meta-analysis of mean 6MWT of studies with participant mean Age <60 during follow-up post COVID-19 infection. Black box, effect estimates from single studies; Diamond, pooled proportion with confidence interval; Weight (in %), influence an individual study had on the pooled result.

Study	Mean	SD	Population	Weight	Mean IV, Random, 95%CI
Anastasio et al	520.00	103.7037	379	2.1%	520.00 [509.56; 530.44]
Aparisi et al	558.00	85.1852	70	2.1%	558.00 [538.04; 577.96]
Blanco et al (low_DLCO)	513.00	107.1111	52	2.0%	513.00 [483.89; 542.11]
Blanco et al(normal_DLCO)	577.00	77.7778	48	2.1%	577.00 [555.00; 599.00]
Calvo-Paniagua et al	560.10	98.9000	68	2.0%	560.10 [536.59; 583.61]
Cao et al	552.00	54.7000	40	2.1%	552.00 [535.05; 568.95]
Cherrez-Ojeda et al	376.90	108.4000	43	1.9%	376.90 [344.50; 409.30]
Clavario et al	540.00	89.8519	200	2.1%	540.00 [527.55; 552.45]
Gochicoa-Rangel et al (IMV)	503.00	233.3333	85	1.7%	503.00 [453.40; 552.60]
Gochicoa-Rangel et al (no_IMV)	567.00	182.9630	86	1.9%	567.00 [528.33; 605.67]
Guler et al (mild)	576.00	78.0000	38	2.0%	576.00 [551.20; 600.80]
Gulhan et al	538.00	57.0000	53	2.1%	538.00 [522.65; 553.35]
Huang, C et al	495.00	72.5926	1692	2.2%	495.00 [491.54; 498.46]
Huang, L et al	495.00	74.8148	1248	2.1%	495.00 [490.85; 499.15]
Huang, L. et al 2	512.00	77.7778	1065	2.1%	512.00 [507.33; 516.67]
Jacobson et al (hosp)	376.00	72.5000	22	2.0%	376.00 [345.70; 406.30]
Jacobson et al (nohosp)	426.00	85.0000	96	2.1%	426.00 [409.00; 443.00]
Karoli et al (mod)	440.50	16.0000	64	2.1%	440.50 [436.58; 444.42]
Karoli et al (severe)	385.60	22.0000	38	2.1%	385.60 [378.61; 392.59]
Kersten et al (hosp)	497.30	90.8000	25	1.9%	497.30 [461.71; 532.89]
Kersten et al (no_symptom)	536.00	84.0000	47	2.0%	536.00 [511.99; 560.01]
Kersten et al (symptom)	523.80	81.1000	295	2.1%	523.80 [514.55; 533.05]
Kooner et al (hosp)	405.00	86.0000	23	1.9%	405.00 [369.85; 440.15]
Kooner et al (nohosp)	471.00	76.0000	53	2.1%	471.00 [450.54; 491.46]
Larsson et al	522.00	148.8889	45	1.8%	522.00 [478.50; 565.50]
Liao et al	555.00	55.5556	303	2.1%	555.00 [548.74; 561.26]
Lorent et al	606.00	111.8519	222	2.1%	606.00 [591.29; 620.71]
Morin et al	462.00	94.0741	161	2.1%	462.00 [447.47; 476.53]
O'Brien et al	421.00	92.0000	55	2.0%	421.00 [396.69; 445.31]
Pogosova et al	480.00	59.2593	213	2.1%	480.00 [472.04; 487.96]
Riou et al (mild)	452.00	113.3333	21	1.7%	452.00 [403.53; 500.47]
Riou et al (severe)	480.00	88.8889	15	1.8%	480.00 [435.02; 524.98]
Saluja et al	540.00	2.4000	166	2.2%	540.00 [539.63; 540.37]
Shogenova et al (control)	397.00	30.0000	30	2.1%	397.00 [386.26; 407.74]
Shogenova et al (treatment)	430.00	45.0000	30	2.1%	430.00 [413.90; 446.10]
Sivori et al (moderate)	458.50	92.7000	82	2.1%	458.50 [438.44; 478.56]
Sivori et al (severe)	409.50	123.2000	79	2.0%	409.50 [382.33; 436.67]
Sneller et al	560.00	78.5185	189	2.1%	560.00 [548.81; 571.19]
Tauekelova et al (abnormal spiro)	381.00	78.5185	169	2.1%	381.00 [369.16; 392.84]
Tauekelova et al (normal spiro)	400.00	74.0741	143	2.1%	400.00 [387.86; 412.14]
Townsend et al	480.00	88.8889	71	2.1%	480.00 [459.32; 500.68]
Uz et al	518.50	55.5600	99	2.1%	518.50 [507.56; 529.44]
Wahlgren et al	475.50	147.0000	108	2.0%	475.50 [447.78; 503.22]
Yang et al	486.00	88.8889	92	2.1%	486.00 [467.84; 504.16]
Yildirim et al (ICU)	445.00	125.1852	31	1.8%	445.00 [400.93; 489.07]
Yildirim et al (non_ICU)	461.00	101.4815	39	1.9%	461.00 [429.15; 492.85]
Zhang et al	500.00	66.6667	280	2.1%	500.00 [492.19; 507.81]
Zhao et al	504.00	39.7333	94	2.1%	504.00 [495.97; 512.03]
Zhou et al	514.10	73.0000	120	2.1%	514.10 [501.04; 527.16]
**Total (95% CI)**			**8687**	**100.0%**	**487.76 [473.35; 502.17]**

Heterogeneity: $Tau^2 = 2510.6016$; $Chi^2 = 8930.12$, df = 48 (P = 0); $I^2 = 99\%$

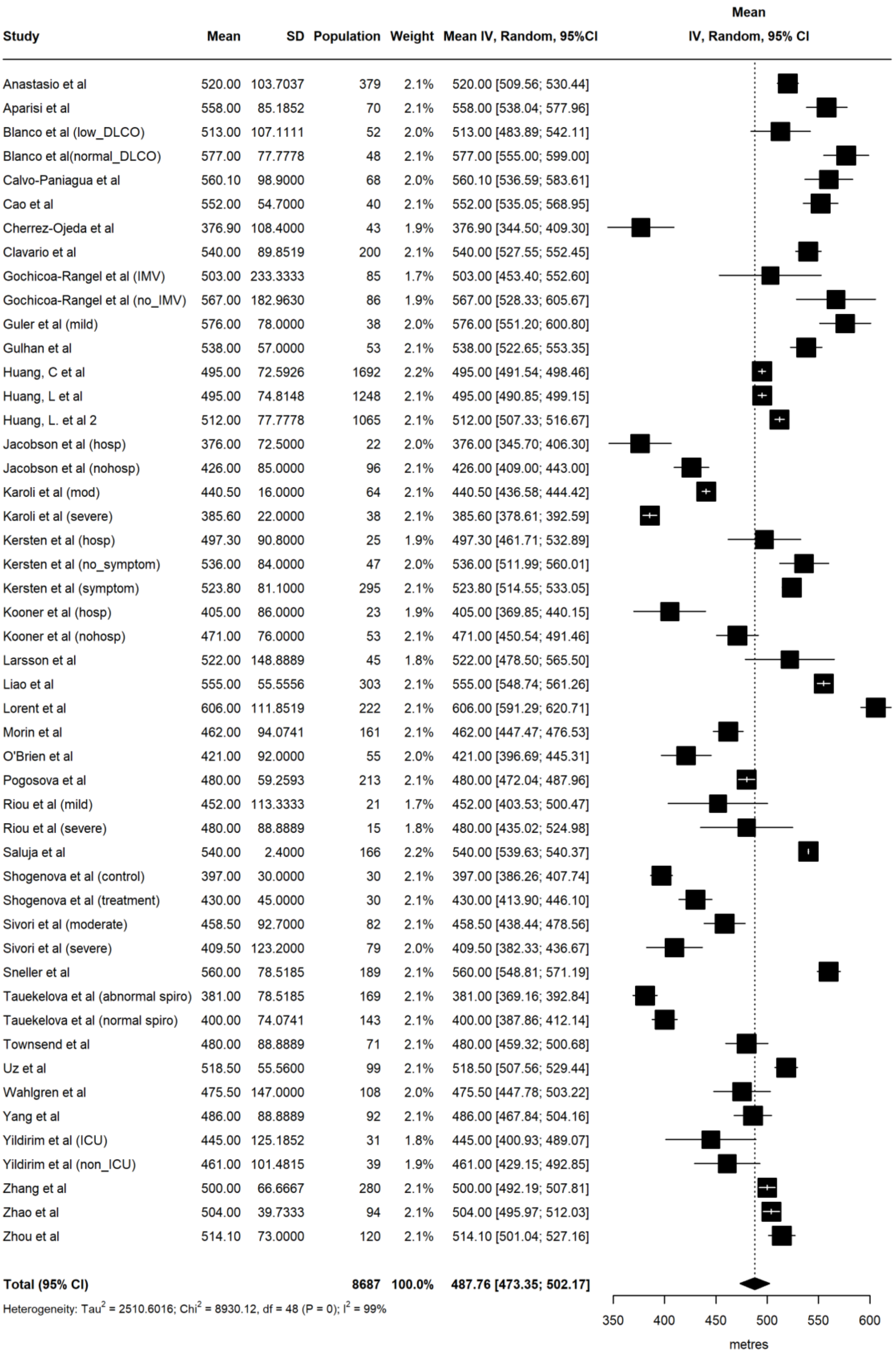

Supplementary Figure 22: Meta-analysis of mean 6MWT of studies with participant mean Age ≥60 during follow-up post COVID-19 infection. Black box, effect estimates from single studies; Diamond, pooled proportion with confidence interval; Weight (in %), influence an individual study had on the pooled result.

Study	Mean	SD	Population	Weight	Mean IV, Random, 95%CI
Aiello et al	404.50	77.1100	121	3.2%	404.50 [390.76; 418.24]
Aranda et al	419.00	136.0000	146	3.1%	419.00 [396.94; 441.06]
Aranda et al 2	377.00	117.0000	113	3.1%	377.00 [355.43; 398.57]
Betschart et al	578.00	129.0000	35	2.6%	578.00 [535.26; 620.74]
Bretas et al (ICU)	466.60	83.6000	92	3.2%	466.60 [449.52; 483.68]
Bretas et al (Ward)	462.00	111.6000	102	3.1%	462.00 [440.34; 483.66]
Damanti et al 2	460.00	72.2222	67	3.2%	460.00 [442.71; 477.29]
Eberst et al	481.00	121.4815	79	3.0%	481.00 [454.21; 507.79]
Faverio et al (CPAP)	473.00	72.5926	144	3.2%	473.00 [461.14; 484.86]
Faverio et al (ICU)	485.00	99.1111	97	3.1%	485.00 [465.28; 504.72]
Faverio et al (oxygen)	450.00	74.0741	71	3.2%	450.00 [432.77; 467.23]
Faverio et al 2 (CPAP)	460.00	81.4815	136	3.2%	460.00 [446.31; 473.69]
Faverio et al 2 (ICU)	475.00	92.5926	90	3.1%	475.00 [455.87; 494.13]
Faverio et al 2 (oxygen)	470.00	83.7037	61	3.1%	470.00 [448.99; 491.01]
Ferioli et al	489.00	141.0000	75	2.9%	489.00 [457.09; 520.91]
Gianella et al	539.30	102.8000	39	2.8%	539.30 [507.04; 571.56]
Guler et al (severe)	456.00	105.0000	57	3.0%	456.00 [428.74; 483.26]
Janssen et al (control)	473.40	124.3000	52	2.8%	473.40 [439.62; 507.18]
Janssen et al (treated)	445.60	126.6000	66	2.9%	445.60 [415.06; 476.14]
Kattainen et al	548.00	75.5556	55	3.1%	548.00 [528.03; 567.97]
Landi et al	535.40	86.2000	66	3.1%	535.40 [514.60; 556.20]
Latronico et al	420.00	100.0000	98	3.1%	420.00 [400.20; 439.80]
Marando et al	539.30	102.8000	39	2.8%	539.30 [507.04; 571.56]
Martino et al (low_DLCO)	461.50	115.5556	28	2.6%	461.50 [418.70; 504.30]
Martino et al (normal_DLCO)	517.50	88.8889	31	2.9%	517.50 [486.21; 548.79]
Muller et al	484.00	110.0000	210	3.2%	484.00 [469.12; 498.88]
Ribeiro Baptista et al	485.18	111.0800	105	3.1%	485.18 [463.93; 506.43]
Riou et al (critical)	435.00	73.3333	45	3.1%	435.00 [413.57; 456.43]
Shah et al	504.00	107.0000	60	3.0%	504.00 [476.93; 531.07]
Sperling et al	486.90	113.2500	218	3.2%	486.90 [471.87; 501.93]
Van Gassel et al	480.00	115.5556	46	2.8%	480.00 [446.61; 513.39]
Vejen et al	463.00	135.5556	123	3.0%	463.00 [439.04; 486.96]
Wu, X et al	535.00	55.5556	83	3.2%	535.00 [523.05; 546.95]
**Total (95% CI)**			**2850**	**100.0%**	**476.71 [462.37; 491.04]**

Heterogeneity: $Tau^2 = 1610.4428$; $Chi^2 = 523.95$, df = 32 (P < 0.01); $I^2 = 94\%$

Mean IV, Random, 95% CI

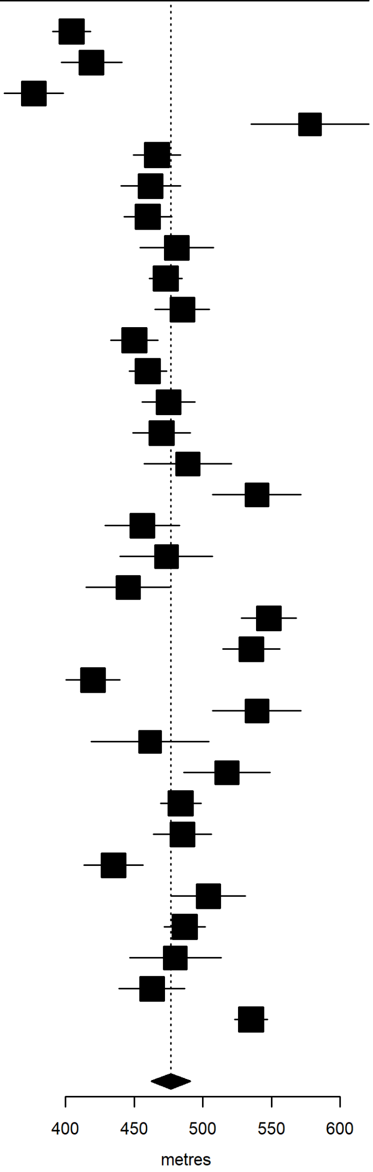

Supplementary Figure 23: Meta-analysis of mean of percentage predicted 6MWT of studies with participant mean Age <60 during follow-up post COVID-19 infection. Black box, effect estimates from single studies; Diamond, pooled proportion with confidence interval; Weight (in %), influence an individual study had on the pooled result.

Study	Mean	SD	Population	Weight	Mean IV, Random, 95%CI
Anastasio et al	88.00	16.2963	379	5.4%	88.00 [86.36; 89.64]
Cherrez-Ojeda et al	69.00	17.8000	43	4.9%	69.00 [63.68; 74.32]
Gochicoa-Rangel et al (IMV)	64.80	29.6296	85	4.7%	64.80 [58.50; 71.10]
Gochicoa-Rangel et al (no_IMV)	68.80	21.4815	86	5.1%	68.80 [64.26; 73.34]
Huang, L et al	90.00	12.8148	1248	5.5%	90.00 [89.29; 90.71]
Huang, L. et al 2	94.00	14.3704	1065	5.5%	94.00 [93.14; 94.86]
Jacobson et al (hosp)	54.40	8.6000	22	5.2%	54.40 [50.81; 57.99]
Jacobson et al (nohosp)	60.40	12.1000	96	5.4%	60.40 [57.98; 62.82]
Karoli et al (mod)	94.00	8.0000	64	5.4%	94.00 [92.04; 95.96]
Karoli et al (severe)	80.00	5.0000	38	5.4%	80.00 [78.41; 81.59]
Liao et al	87.00	9.6296	303	5.5%	87.00 [85.92; 88.08]
Lorent et al	89.00	12.5926	222	5.4%	89.00 [87.34; 90.66]
Schandl et al	88.00	18.5185	59	5.0%	88.00 [83.27; 92.73]
Sivori et al (moderate)	91.50	21.4000	82	5.0%	91.50 [86.87; 96.13]
Sivori et al (severe)	78.80	21.7000	79	5.0%	78.80 [74.01; 83.59]
van den Borst et al	92.00	18.0000	115	5.3%	92.00 [88.71; 95.29]
Yang et al	89.00	13.3333	92	5.3%	89.00 [86.28; 91.72]
Zhang et al	87.90	13.9259	280	5.4%	87.90 [86.27; 89.53]
Zhou et al	92.40	12.5926	120	5.4%	92.40 [90.15; 94.65]
**Total (95% CI)**			**4478**	**100.0%**	**82.30 [78.69; 85.92]**

Heterogeneity: $Tau^2 = 61.7296$; $Chi^2 = 1395.08$, df = 18 (P < 0.01); $I^2 = 99\%$

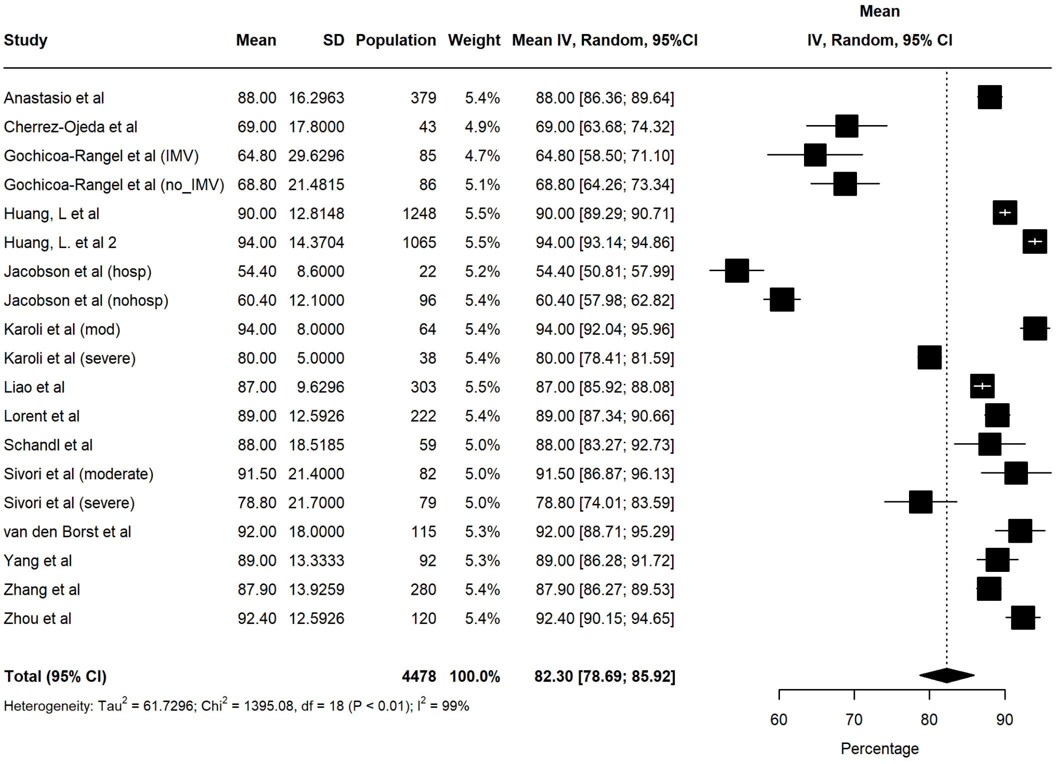

Supplementary Figure 24: Meta-analysis of mean of percentage predicted 6MWT of studies with participant mean Age ≥60 during follow-up post COVID-19 infection. Black box, effect estimates from single studies; Diamond, pooled proportion with confidence interval; Weight (in %), influence an individual study had on the pooled result.

Study	Mean	SD	Population	Weight	Mean IV, Random, 95%CI
Aiello et al	103.90	17.1000	121	6.0%	103.90 [100.85; 106.95]
Betschart et al	102.00	21.0000	35	5.6%	102.00 [95.04; 108.96]
Bretas et al (ICU)	90.00	16.3000	92	6.0%	90.00 [86.67; 93.33]
Bretas et al (Ward)	90.30	18.8000	102	6.0%	90.30 [86.65; 93.95]
Damanti et al 2	92.00	11.1111	67	6.0%	92.00 [89.34; 94.66]
Ferioli et al	73.00	22.0000	75	5.8%	73.00 [68.02; 77.98]
Gonzalez et al	83.70	26.0000	93	5.8%	83.70 [78.42; 88.98]
Janssen et al (control)	74.60	18.3000	52	5.8%	74.60 [69.63; 79.57]
Janssen et al (treated)	71.40	19.0000	66	5.9%	71.40 [66.82; 75.98]
Kattainen et al	107.70	15.1111	55	5.9%	107.70 [103.71; 111.69]
Latronico et al	80.00	22.2222	98	5.9%	80.00 [75.60; 84.40]
Marando et al	99.00	13.6000	39	5.9%	99.00 [94.73; 103.27]
Muller et al	79.10	13.0000	210	6.1%	79.10 [77.34; 80.86]
Noel-Savina et al	92.60	21.4815	72	5.8%	92.60 [87.64; 97.56]
Shah et al	96.00	16.0000	60	5.9%	96.00 [91.95; 100.05]
Staudt et al	101.90	16.8148	99	6.0%	101.90 [98.59; 105.21]
Van Gassel et al	80.80	22.3704	46	5.6%	80.80 [74.34; 87.26]
**Total (95% CI)**			**1382**	**100.0%**	**89.33 [83.97; 94.69]**

Heterogeneity: $Tau^2 = 122.0046$; $Chi^2 = 564.42$, df = 16 (P < 0.01); $I^2 = 97\%$

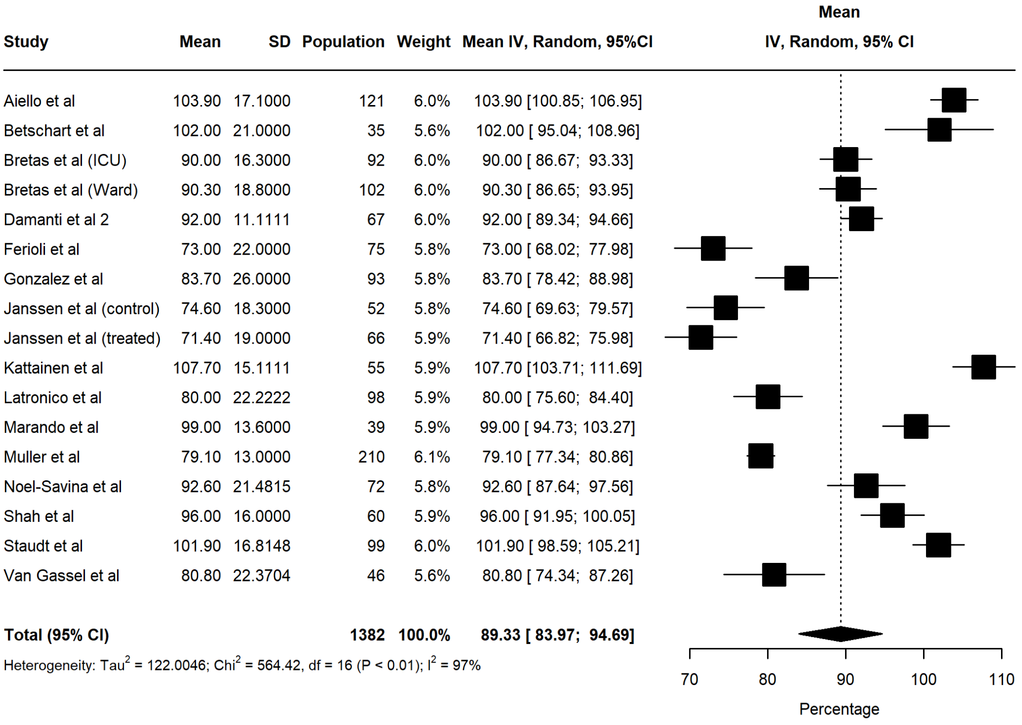

Supplementary Figure 25: Meta-analysis of mean 6MWT of studies with <80% of participants hospitalised during acute COVID-19 infection. Black box, effect estimates from single studies; Diamond, pooled proportion with confidence interval; Weight (in %), influence an individual study had on the pooled result.

**Study**	**Mean**	**SD**	**Population**	**Weight**	**Mean IV, Random, 95%CI**
Aparisi et al	558.00	85.1852	70	6.6%	558.00 [538.04; 577.96]
Calvo-Paniagua et al	560.10	98.9000	68	6.6%	560.10 [536.59; 583.61]
Cherrez-Ojeda et al	376.90	108.4000	43	6.3%	376.90 [344.50; 409.30]
Gulhan et al	538.00	57.0000	53	6.7%	538.00 [522.65; 553.35]
Jacobson et al (nohosp)	426.00	85.0000	96	6.7%	426.00 [409.00; 443.00]
Kersten et al (no_symptom)	536.00	84.0000	47	6.6%	536.00 [511.99; 560.01]
Kersten et al (symptom)	523.80	81.1000	295	6.8%	523.80 [514.55; 533.05]
Kooner et al (nohosp)	471.00	76.0000	53	6.6%	471.00 [450.54; 491.46]
Landi et al	535.40	86.2000	66	6.6%	535.40 [514.60; 556.20]
Muller et al	484.00	110.0000	210	6.7%	484.00 [469.12; 498.88]
Saluja et al	540.00	2.4000	166	6.8%	540.00 [539.63; 540.37]
Sneller et al	560.00	78.5185	189	6.8%	560.00 [548.81; 571.19]
Tauekelova et al (abnormal spiro)	381.00	78.5185	169	6.8%	381.00 [369.16; 392.84]
Tauekelova et al (normal spiro)	400.00	74.0741	143	6.8%	400.00 [387.86; 412.14]
Townsend et al	480.00	88.8889	71	6.6%	480.00 [459.32; 500.68]
**Total (95% CI)**			**1739**	**100.0%**	**491.58 [461.14; 522.01]**

Heterogeneity: $Tau^2 = 3528.9994$; $Chi^2 = 1629.54$, df = 14 (P = 0); $I^2 = 99\%$

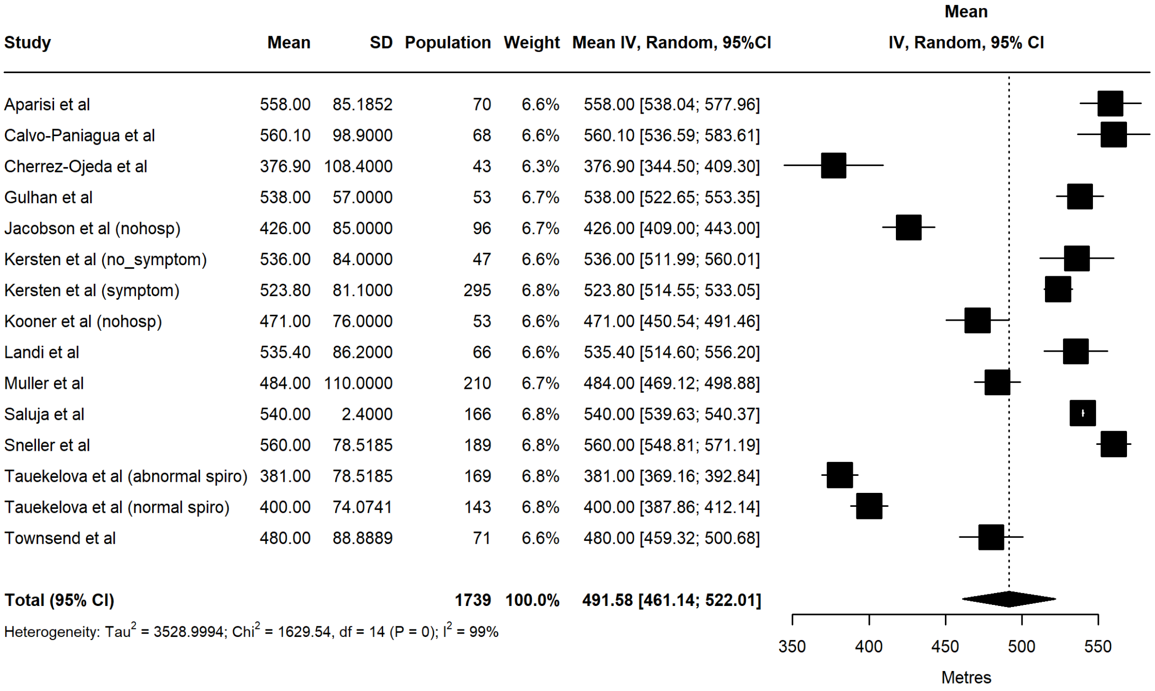

Supplementary Figure 26: Meta-analysis of mean 6MWT of studies with ≥80% of participants hospitalised during acute COVID-19 infection. Black box, effect estimates from single studies; Diamond, pooled proportion with confidence interval; Weight (in %), influence an individual study had on the pooled result.

Study	Mean	SD	Population	Weight	Mean IV, Random, 95%CI
Aiello et al	404.50	77.1100	121	1.6%	404.50 [390.76; 418.24]
Aranda et al	419.00	136.0000	146	1.5%	419.00 [396.94; 441.06]
Aranda et al 2	377.00	117.0000	113	1.5%	377.00 [355.43; 398.57]
Bardakci et al (abnormal_CT)	492.00	117.7778	44	1.4%	492.00 [457.20; 526.80]
Bardakci et al (Normal_CT)	490.00	112.5926	21	1.2%	490.00 [441.84; 538.16]
Betschart et al	578.00	129.0000	35	1.3%	578.00 [535.26; 620.74]
Blanco et al (low_DLCO)	513.00	107.1111	52	1.4%	513.00 [483.89; 542.11]
Blanco et al(normal_DLCO)	577.00	77.7778	48	1.5%	577.00 [555.00; 599.00]
Bretas et al (ICU)	466.60	83.6000	92	1.5%	466.60 [449.52; 483.68]
Bretas et al (Ward)	462.00	111.6000	102	1.5%	462.00 [440.34; 483.66]
Cao et al	552.00	54.7000	40	1.5%	552.00 [535.05; 568.95]
Clavario et al	540.00	89.8519	200	1.6%	540.00 [527.55; 552.45]
Damanti et al 2	460.00	72.2222	67	1.5%	460.00 [442.71; 477.29]
Eberst et al	481.00	121.4815	79	1.5%	481.00 [454.21; 507.79]
Faverio et al (CPAP)	473.00	72.5926	144	1.6%	473.00 [461.14; 484.86]
Faverio et al (ICU)	485.00	99.1111	97	1.5%	485.00 [465.28; 504.72]
Faverio et al (oxygen)	450.00	74.0741	71	1.5%	450.00 [432.77; 467.23]
Faverio et al 2 (CPAP)	460.00	81.4815	136	1.6%	460.00 [446.31; 473.69]
Faverio et al 2 (ICU)	475.00	92.5926	90	1.5%	475.00 [455.87; 494.13]
Faverio et al 2 (oxygen)	470.00	83.7037	61	1.5%	470.00 [448.99; 491.01]
Ferioli et al	489.00	141.0000	75	1.4%	489.00 [457.09; 520.91]
Gianella et al	539.30	102.8000	39	1.4%	539.30 [507.04; 571.56]
Gochicoa-Rangel et al (IMV)	503.00	233.3333	85	1.2%	503.00 [453.40; 552.60]
Gochicoa-Rangel et al (no_IMV)	567.00	182.9630	86	1.3%	567.00 [528.33; 605.67]
Guler et al (mild)	576.00	78.0000	38	1.5%	576.00 [551.20; 600.80]
Guler et al (severe)	456.00	105.0000	57	1.5%	456.00 [428.74; 483.26]
Huang, C et al	495.00	72.5926	1692	1.6%	495.00 [491.54; 498.46]
Huang, L et al	495.00	74.8148	1248	1.6%	495.00 [490.85; 499.15]
Huang, L. et al 2	512.00	77.7778	1065	1.6%	512.00 [507.33; 516.67]
Jacobson et al (hosp)	376.00	72.5000	22	1.4%	376.00 [345.70; 406.30]
Janssen et al (control)	473.40	124.3000	52	1.4%	473.40 [439.62; 507.18]
Janssen et al (treated)	445.60	126.6000	66	1.4%	445.60 [415.06; 476.14]
Karoli et al (mod)	440.50	16.0000	64	1.6%	440.50 [436.58; 444.42]
Karoli et al (severe)	385.60	22.0000	38	1.6%	385.60 [378.61; 392.59]
Kattainen et al	548.00	75.5556	55	1.5%	548.00 [528.03; 567.97]
Kersten et al (hosp)	497.30	90.8000	25	1.4%	497.30 [461.71; 532.89]
Kooner et al (hosp)	405.00	86.0000	23	1.4%	405.00 [369.85; 440.15]
Larsson et al	522.00	148.8889	45	1.3%	522.00 [478.50; 565.50]
Latronico et al	420.00	100.0000	98	1.5%	420.00 [400.20; 439.80]
Liao et al	555.00	55.5556	303	1.6%	555.00 [548.74; 561.26]
Lorent et al	606.00	111.8519	222	1.5%	606.00 [591.29; 620.71]
Marando et al	539.30	102.8000	39	1.4%	539.30 [507.04; 571.56]
Martino et al (low_DLCO)	461.50	115.5556	28	1.3%	461.50 [418.70; 504.30]
Martino et al (normal_DLCO)	517.50	88.8889	31	1.4%	517.50 [486.21; 548.79]
Morin et al	462.00	94.0741	161	1.6%	462.00 [447.47; 476.53]
O'Brien et al	421.00	92.0000	55	1.5%	421.00 [396.69; 445.31]
Pogosova et al	480.00	59.2593	213	1.6%	480.00 [472.04; 487.96]
Ribeiro Baptista et al	485.18	111.0800	105	1.5%	485.18 [463.93; 506.43]
Riou et al (critical)	435.00	73.3333	45	1.5%	435.00 [413.57; 456.43]
Riou et al (mild)	452.00	113.3333	21	1.2%	452.00 [403.53; 500.47]
Riou et al (severe)	480.00	88.8889	15	1.3%	480.00 [435.02; 524.98]
Shah et al	504.00	107.0000	60	1.5%	504.00 [476.93; 531.07]
Shogenova et al (control)	397.00	30.0000	30	1.6%	397.00 [386.26; 407.74]
Shogenova et al (treatment)	430.00	45.0000	30	1.5%	430.00 [413.90; 446.10]
Sivori et al (moderate)	458.50	92.7000	82	1.5%	458.50 [438.44; 478.56]
Sivori et al (severe)	409.50	123.2000	79	1.5%	409.50 [382.33; 436.67]
Sperling et al	486.90	113.2500	218	1.5%	486.90 [471.87; 501.93]
Uz et al	518.50	55.5600	99	1.6%	518.50 [507.56; 529.44]
Van Gassel et al	480.00	115.5556	46	1.4%	480.00 [446.61; 513.39]
Vejen et al	463.00	135.5556	123	1.5%	463.00 [439.04; 486.96]
Wahlgren et al	475.50	147.0000	108	1.4%	475.50 [447.78; 503.22]
Wu, X et al	535.00	55.5556	83	1.6%	535.00 [523.05; 546.95]
Yang et al	486.00	88.8889	92	1.5%	486.00 [467.84; 504.16]
Yildirim et al (ICU)	445.00	125.1852	31	1.3%	445.00 [400.93; 489.07]
Yildirim et al (non_ICU)	461.00	101.4815	39	1.4%	461.00 [429.15; 492.85]
Zhang et al	500.00	66.6667	280	1.6%	500.00 [492.19; 507.81]
Zhao et al	504.00	39.7333	94	1.6%	504.00 [495.97; 512.03]
Zhou et al	514.10	73.0000	120	1.6%	514.10 [501.04; 527.16]
**Total (95% CI)**			**9484**	**100.0%**	**481.17 [470.09; 492.26]**

Heterogeneity: $Tau^2 = 2009.5985$; $Chi^2 = 3559.37$, df = 67 (P = 0); $I^2 = 98\%$

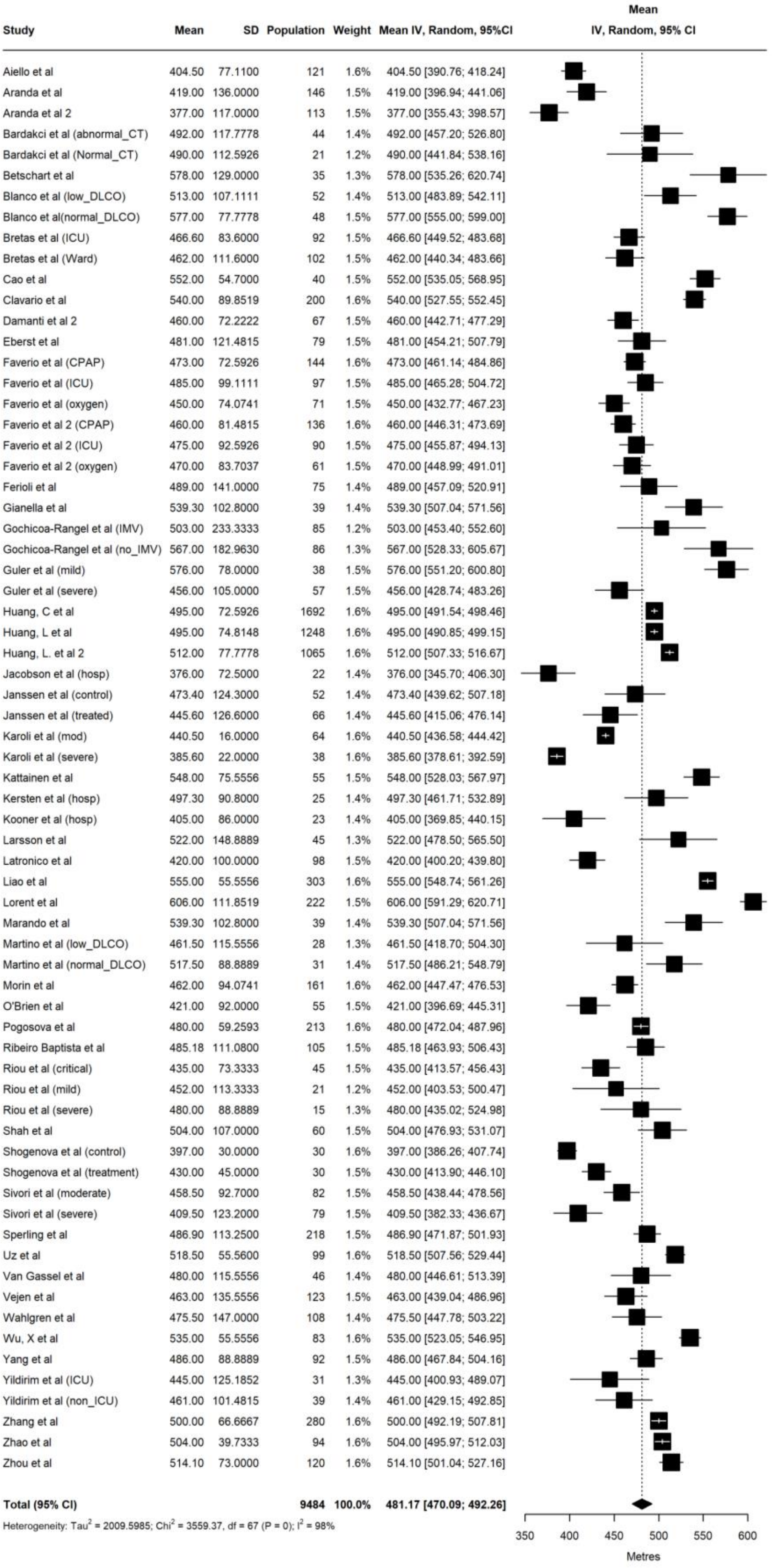

Supplementary Figure 27: Meta-analysis of mean of percentage predicted 6MWT of studies with <80% of participants hospitalised during acute COVID-19 infection. Black box, effect estimates from single studies; Diamond, pooled proportion with confidence interval; Weight (in %), influence an individual study had on the pooled result.

Study	Mean	SD	Population	Weight	Mean IV, Random, 95%CI
Cherrez-Ojeda et al	69.00	17.8000	43	24.4%	69.00 [63.68; 74.32]
Jacobson et al (nohosp)	60.40	12.1000	96	25.2%	60.40 [57.98; 62.82]
Muller et al	79.10	13.0000	210	25.3%	79.10 [77.34; 80.86]
van den Borst et al	92.00	18.0000	115	25.0%	92.00 [88.71; 95.29]
**Total (95% CI)**			**464**	**100.0%**	**75.15 [62.02; 88.27]**

Heterogeneity: $Tau^2 = 176.3336$; $Chi^2 = 267.89$, df = 3 ($P < 0.01$); $I^2 = 99\%$

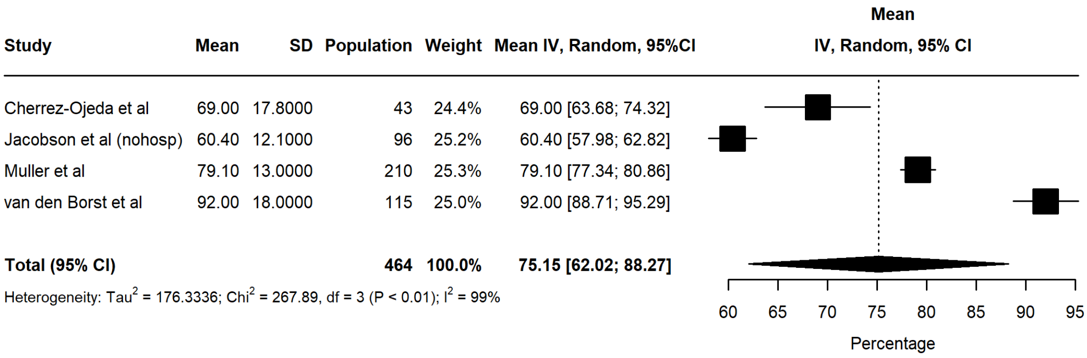

Supplementary Figure 28: Meta-analysis of mean of percentage predicted 6MWT of studies with ≥80% of participants hospitalised during acute COVID-19 infection. Black box, effect estimates from single studies; Diamond, pooled proportion with confidence interval; Weight (in %), influence an individual study had on the pooled result.

Study	Mean	SD	Population	Weight	Mean IV, Random, 95%CI
Aiello et al	103.90	17.1000	121	3.3%	103.90 [100.85; 106.95]
Betschart et al	102.00	21.0000	35	2.8%	102.00 [95.04; 108.96]
Bretas et al (ICU)	90.00	16.3000	92	3.3%	90.00 [86.67; 93.33]
Bretas et al (Ward)	90.30	18.8000	102	3.3%	90.30 [86.65; 93.95]
Damanti et al 2	92.00	11.1111	67	3.4%	92.00 [89.34; 94.66]
Ferioli et al	73.00	22.0000	75	3.1%	73.00 [68.02; 77.98]
Gochicoa-Rangel et al (IMV)	64.80	29.6296	85	2.9%	64.80 [58.50; 71.10]
Gochicoa-Rangel et al (no_IMV)	68.80	21.4815	86	3.2%	68.80 [64.26; 73.34]
Gonzalez et al	83.70	26.0000	93	3.0%	83.70 [78.42; 88.98]
Huang, L et al	90.00	12.8148	1248	3.5%	90.00 [89.29; 90.71]
Huang, L. et al 2	94.00	14.3704	1065	3.5%	94.00 [93.14; 94.86]
Jacobson et al (hosp)	54.40	8.6000	22	3.3%	54.40 [50.81; 57.99]
Janssen et al (control)	74.60	18.3000	52	3.1%	74.60 [69.63; 79.57]
Janssen et al (treated)	71.40	19.0000	66	3.1%	71.40 [66.82; 75.98]
Karoli et al (mod)	94.00	8.0000	64	3.4%	94.00 [92.04; 95.96]
Karoli et al (severe)	80.00	5.0000	38	3.4%	80.00 [78.41; 81.59]
Kattainen et al	107.70	15.1111	55	3.2%	107.70 [103.71; 111.69]
Latronico et al	80.00	22.2222	98	3.2%	80.00 [75.60; 84.40]
Liao et al	87.00	9.6296	303	3.5%	87.00 [85.92; 88.08]
Lorent et al	89.00	12.5926	222	3.4%	89.00 [87.34; 90.66]
Marando et al	99.00	13.6000	39	3.2%	99.00 [94.73; 103.27]
Noel-Savina et al	92.60	21.4815	72	3.1%	92.60 [87.64; 97.56]
Schandl et al	88.00	18.5185	59	3.1%	88.00 [83.27; 92.73]
Shah et al	96.00	16.0000	60	3.2%	96.00 [91.95; 100.05]
Sivori et al (moderate)	91.50	21.4000	82	3.1%	91.50 [86.87; 96.13]
Sivori et al (severe)	78.80	21.7000	79	3.1%	78.80 [74.01; 83.59]
Staudt et al	101.90	16.8148	99	3.3%	101.90 [98.59; 105.21]
Van Gassel et al	80.80	22.3704	46	2.9%	80.80 [74.34; 87.26]
Yang et al	89.00	13.3333	92	3.4%	89.00 [86.28; 91.72]
Zhang et al	87.90	13.9259	280	3.4%	87.90 [86.27; 89.53]
Zhou et al	92.40	12.5926	120	3.4%	92.40 [90.15; 94.65]
**Total (95% CI)**			**5017**	**100.0%**	**86.88 [84.26; 89.50]**

Heterogeneity: $Tau^2 = 51.4698$; $Chi^2 = 1252.39$, df = 30 ($P < 0.01$); $I^2 = 98\%$

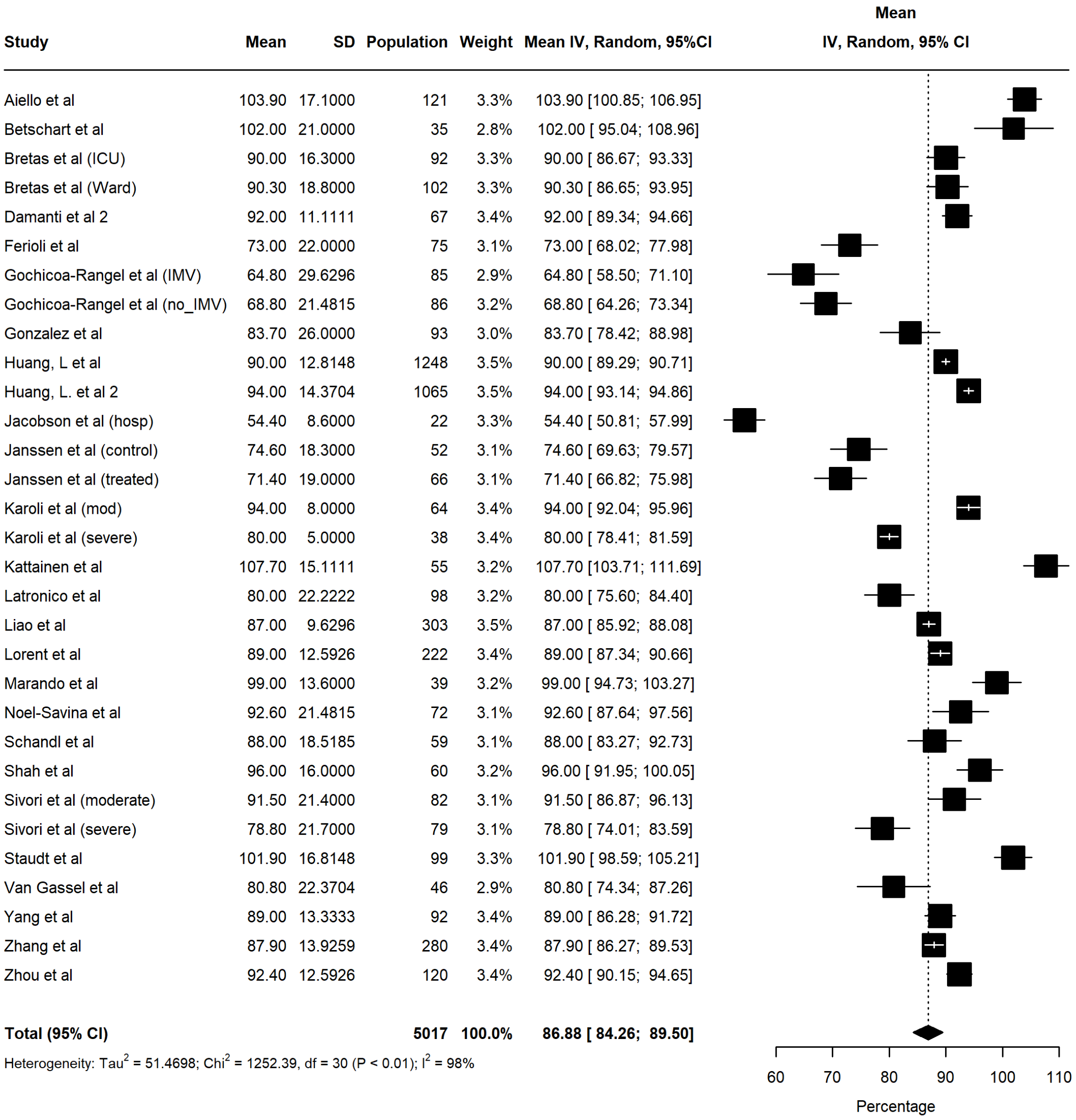

Supplementary Figure 29: Meta-analysis of mean 6MWT of studies of hospitalised patients with <20% of participants admitted to ICU during acute COVID-19 infection. Black box, effect estimates from single studies; Diamond, pooled proportion with confidence interval; Weight (in %), influence an individual study had on the pooled result.

Study	Mean	SD	Population	Weight	Mean IV, Random, 95%CI
Aranda et al	419.00	136.0000	146	6.3%	419.00 [396.94; 441.06]
Bardakci et al (abnormal_CT)	492.00	117.7778	44	5.6%	492.00 [457.20; 526.80]
Bardakci et al (Normal_CT)	490.00	112.5926	21	4.8%	490.00 [441.84; 538.16]
Bretas et al (Ward)	462.00	111.6000	102	6.3%	462.00 [440.34; 483.66]
Huang, C et al	495.00	72.5926	1692	6.9%	495.00 [491.54; 498.46]
Huang, L et al	495.00	74.8148	1248	6.9%	495.00 [490.85; 499.15]
Huang, L. et al 2	512.00	77.7778	1065	6.9%	512.00 [507.33; 516.67]
Karoli et al (mod)	440.50	16.0000	64	6.9%	440.50 [436.58; 444.42]
Karoli et al (severe)	385.60	22.0000	38	6.8%	385.60 [378.61; 392.59]
Pogosova et al	480.00	59.2593	213	6.8%	480.00 [472.04; 487.96]
Riou et al (mild)	452.00	113.3333	21	4.8%	452.00 [403.53; 500.47]
Riou et al (severe)	480.00	88.8889	15	5.0%	480.00 [435.02; 524.98]
Sperling et al	486.90	113.2500	218	6.6%	486.90 [471.87; 501.93]
Yildirim et al (non_ICU)	461.00	101.4815	39	5.8%	461.00 [429.15; 492.85]
Zhang et al	500.00	66.6667	280	6.8%	500.00 [492.19; 507.81]
Zhao et al	504.00	39.7333	94	6.8%	504.00 [495.97; 512.03]
**Total (95% CI)**			**5300**	**100.0%**	**472.27 [453.19; 491.36]**

Heterogeneity: $Tau^2 = 1374.9636$; $Chi^2 = 1486.52$, df = 15 ($P < 0.01$); $I^2 = 99\%$

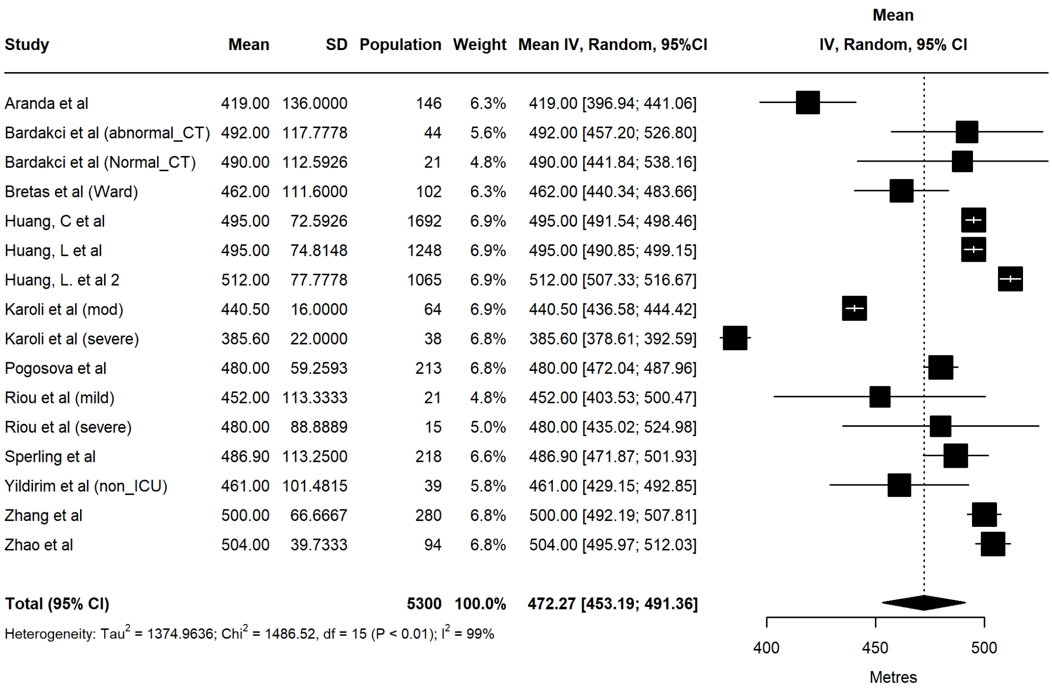

Supplementary Figure 30: Meta-analysis of mean 6MWT of studies of hospitalised patients with 20% to <80% of participants admitted to ICU during acute COVID-19 infection. Black box, effect estimates from single studies; Diamond, pooled proportion with confidence interval; Weight (in %), influence an individual study had on the pooled result.

Study	Mean	SD	Population	Weight	Mean IV, Random, 95%CI
Aranda et al 2	377.00	117.0000	113	12.6%	377.00 [355.43; 398.57]
Gianella et al	539.30	102.8000	39	12.3%	539.30 [507.04; 571.56]
Jacobson et al (hosp)	376.00	72.5000	22	12.4%	376.00 [345.70; 406.30]
Lorent et al	606.00	111.8519	222	12.7%	606.00 [591.29; 620.71]
Marando et al	539.30	102.8000	39	12.3%	539.30 [507.04; 571.56]
Morin et al	462.00	94.0741	161	12.7%	462.00 [447.47; 476.53]
O'Brien et al	421.00	92.0000	55	12.5%	421.00 [396.69; 445.31]
Ribeiro Baptista et al	485.18	111.0800	105	12.6%	485.18 [463.93; 506.43]
**Total (95% CI)**			**756**	**100.0%**	**475.76 [413.99; 537.53]**

Heterogeneity: $Tau^2 = 7786.2599$; $Chi^2 = 461.91$, df = 7 ($P < 0.01$); $I^2 = 98\%$

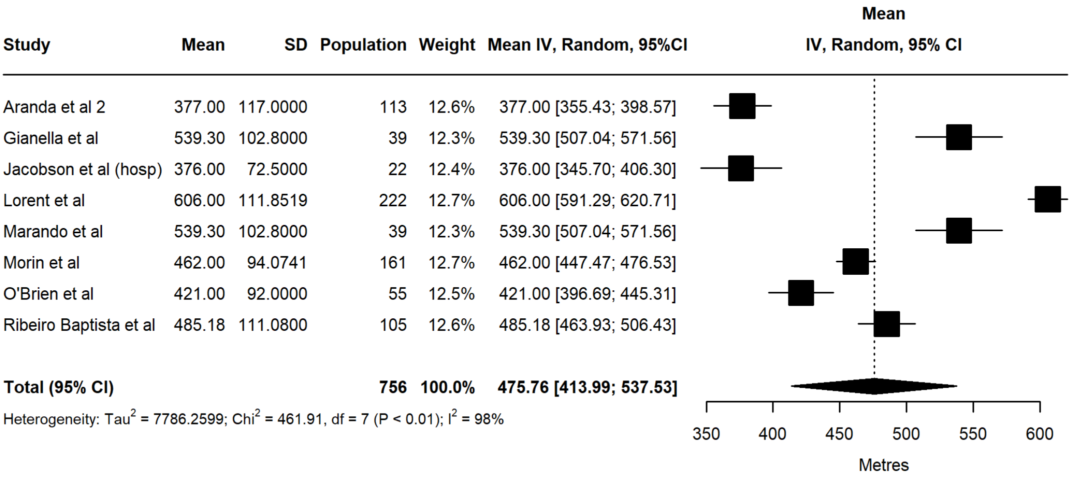

Supplementary Figure 31: Meta-analysis of mean 6MWT of studies of hospitalised patients with ≥80% of participants admitted to ICU during acute COVID-19 infection. Black box, effect estimates from single studies; Diamond, pooled proportion with confidence interval; Weight (in %), influence an individual study had on the pooled result.

Study	Mean	SD	Population	Weight	Mean IV, Random, 95%CI
Bretas et al (ICU)	466.60	83.6000	92	11.4%	466.60 [449.52; 483.68]
Eberst et al	481.00	121.4815	79	10.4%	481.00 [454.21; 507.79]
Faverio et al (ICU)	485.00	99.1111	97	11.1%	485.00 [465.28; 504.72]
Faverio et al 2 (ICU)	475.00	92.5926	90	11.2%	475.00 [455.87; 494.13]
Gochicoa-Rangel et al (IMV)	503.00	233.3333	85	7.7%	503.00 [453.40; 552.60]
Kattainen et al	548.00	75.5556	55	11.1%	548.00 [528.03; 567.97]
Larsson et al	522.00	148.8889	45	8.4%	522.00 [478.50; 565.50]
Riou et al (critical)	435.00	73.3333	45	11.0%	435.00 [413.57; 456.43]
Van Gassel et al	480.00	115.5556	46	9.6%	480.00 [446.61; 513.39]
Yildirim et al (ICU)	445.00	125.1852	31	8.3%	445.00 [400.93; 489.07]
**Total (95% CI)**			**665**	**100.0%**	**483.56 [461.03; 506.10]**

Heterogeneity: Tau^2 = 1087.0574; Chi^2 = 70.83, df = 9 (P < 0.01); I^2 = 87%

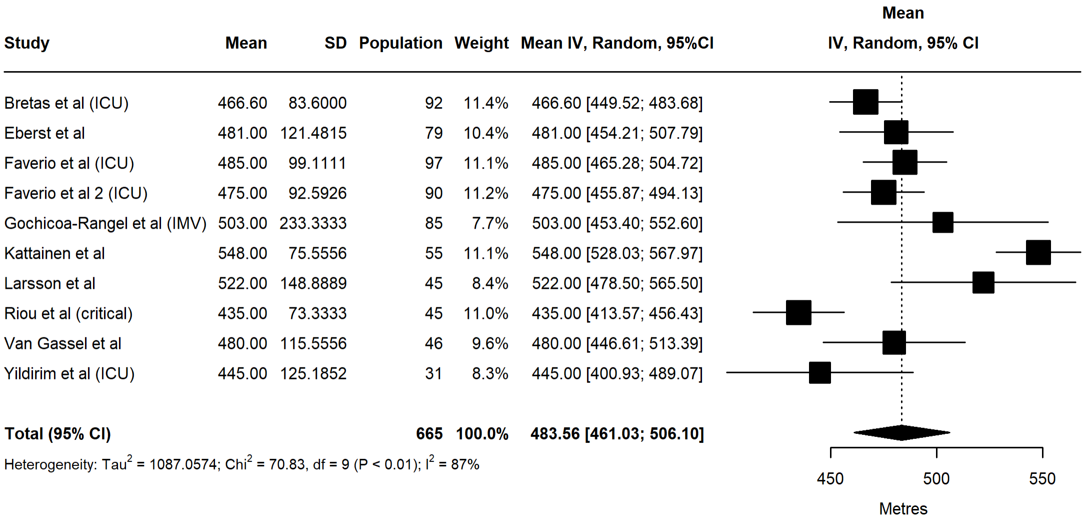

Supplementary Figure 32: Meta-analysis of mean of percentage predicted 6MWT in studies of hospitalised patients with <20% of participants admitted to ICU during acute COVID-19 infection. Black box, effect estimates from single studies; Diamond, pooled proportion with confidence interval; Weight (in %), influence an individual study had on the pooled result.

Study	Mean	SD	Population	Weight	Mean IV, Random, 95%CI
Bretas et al (Ward)	90.30	18.8000	102	13.1%	90.30 [86.65; 93.95]
Huang, L et al	90.00	12.8148	1248	14.9%	90.00 [89.29; 90.71]
Huang, L. et al 2	94.00	14.3704	1065	14.9%	94.00 [93.14; 94.86]
Karoli et al (mod)	94.00	8.0000	64	14.4%	94.00 [92.04; 95.96]
Karoli et al (severe)	80.00	5.0000	38	14.6%	80.00 [78.41; 81.59]
Staudt et al	101.90	16.8148	99	13.4%	101.90 [98.59; 105.21]
Zhang et al	87.90	13.9259	280	14.6%	87.90 [86.27; 89.53]
**Total (95% CI)**			**2896**	**100.0%**	**91.04 [87.31; 94.77]**

Heterogeneity: Tau^2 = 24.1393; Chi^2 = 299.96, df = 6 (P < 0.01); I^2 = 98%

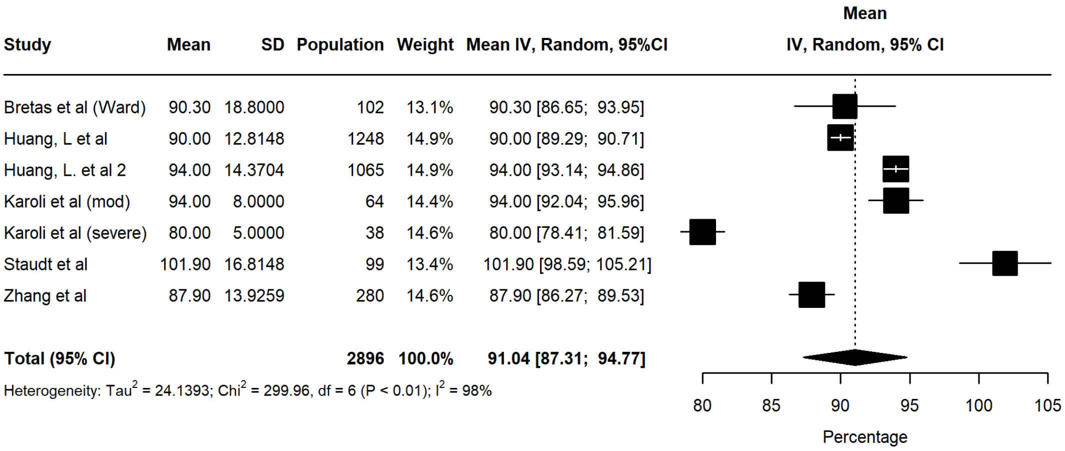

Supplementary Figure 33: Meta-analysis of mean of percentage predicted 6MWT in studies of hospitalised patients with 20% to <80% of participants admitted to ICU during acute COVID-19 infection. Black box, effect estimates from single studies; Diamond, pooled proportion with confidence interval; Weight (in %), influence an individual study had on the pooled result.

Study	Mean	SD	Population	Weight	Mean IV, Random, 95%CI
Jacobson et al (hosp)	54.40	8.6000	22	25.0%	54.40 [50.81; 57.99]
Lorent et al	89.00	12.5926	222	25.2%	89.00 [87.34; 90.66]
Marando et al	99.00	13.6000	39	24.9%	99.00 [94.73; 103.27]
Noel-Savina et al	92.60	21.4815	72	24.8%	92.60 [87.64; 97.56]
**Total (95% CI)**			**355**	**100.0%**	**83.73 [65.68; 101.77]**

Heterogeneity: Tau^2 = 335.1055; Chi^2 = 351.07, df = 3 (P < 0.01); I^2 = 99%

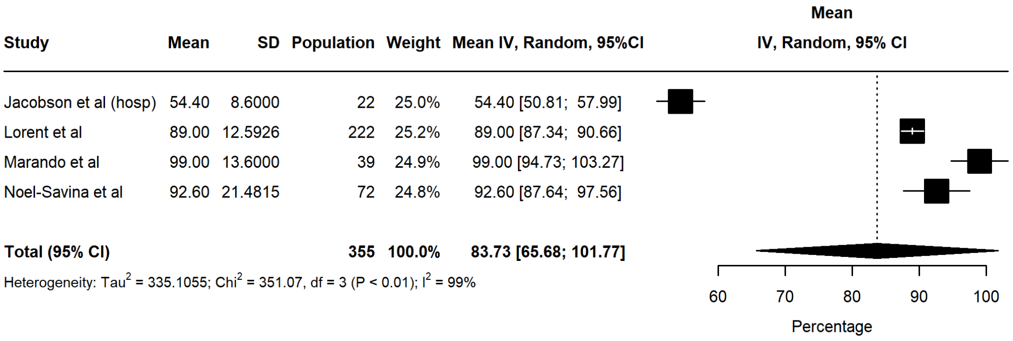

Supplementary Figure 34: Meta-analysis of mean of percentage predicted 6MWT in studies of hospitalised patients with ≥80% of participants admitted to ICU during acute COVID-19 infection. Black box, effect estimates from single studies; Diamond, pooled proportion with confidence interval; Weight (in %), influence an individual study had on the pooled result.

Study	Mean	SD	Population	Weight	Mean IV, Random, 95%CI
Bretas et al (ICU)	90.00	16.3000	92	17.0%	90.00 [86.67; 93.33]
Gochicoa-Rangel et al (IMV)	64.80	29.6296	85	16.3%	64.80 [58.50; 71.10]
Gonzalez et al	83.70	26.0000	93	16.6%	83.70 [78.42; 88.98]
Kattainen et al	107.70	15.1111	55	16.9%	107.70 [103.71; 111.69]
Schandl et al	88.00	18.5185	59	16.8%	88.00 [83.27; 92.73]
Van Gassel et al	80.80	22.3704	46	16.3%	80.80 [74.34; 87.26]
**Total (95% CI)**			**430**	**100.0%**	**86.00 [75.37; 96.62]**

Heterogeneity: $Tau^2 = 169.4710$; $Chi^2 = 150.82$, df = 5 ($P < 0.01$); $I^2 = 97\%$

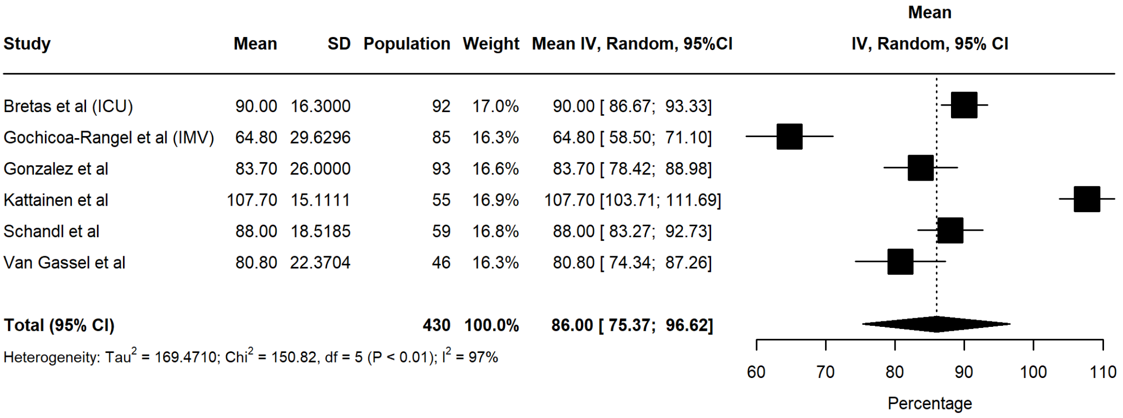

Supplement: 2023-0272_R2_Forest_plots_revision_SM_pzae023 [file 2023-0272_r2_forest_plots_revision_sm_pzae023.pdf]
