# Supplementary material for: Functional Recovery of Adults Following Acute COVID-19: A Systematic Review and Meta-Analysis
Source: Phys Ther. 2024 Feb 22;105(1):pzae023. doi: 10.1093/ptj/pzae023 (PMC11738174; doi:10.1093/ptj/pzae023)
Supplement: 2023-0272_R2_Supplementary_tables_revision_final_pzae023 [file 2023-0272_r2_supplementary_tables_revision_final_pzae023.pdf]

Supplementary Text 1: Search strategy

Coronavirus OR Coronavirinae OR “Severe acute respiratory syndrome” OR "SARS coronavirus" OR "Covid-19"OR "coronavirus disease 2019"OR "SARS-COV-2 OR "long Covid*" OR "long hauler*" OR "post-Covid-19" OR "post Covid-19 syndrome"

AND

recovery OR "follow-up" or "follow up"

AND

ADL OR "exercise tolerance” OR function* OR "functional status” OR "functional performance” OR “functional outcome*” OR "exercise test*" OR "activities of daily living" OR Strength OR “walk test" OR 6MWT OR "six minute walk test" OR "6MWD" OR "shuttle walk test" OR "incremental shuttle walk test" OR ISWT OR "short physical performance battery" OR SPPB OR "sit-to-stand" OR STS OR "chair stand" OR "gait speed" OR balance OR "grip strength" OR "muscle strength OR Barthel index

Supplementary table 1: Summary of included studies. Abbreviations: Not recorded (NR), Invasive mechanical ventilation (IMV), Intensive Care unit (ICU)

Study	Year	Country	Study design	Inclusion Criteria	Exclusion criteria	Participants	Age (years)	Male (%)	BMI (average)	% hospitalised	% requiring ICU	Length of stay (median days)
**Abdallah et al (H)[1]**	2021	Canada	cohort	>18 years of age and diagnosed with SARS-CoV-2 by PCR 3 months (+6wk) before enrollment.	NR	25	59.1	64	NR	100	40	17.12
**Abdallah et al (NH)[1]**						38	42.4	52.6	NR	0	0	0
**Aiello et al[2]**	2022	Italy	cohort	Age ≥18 years with radiologically and laboratory documented diagnosis of COVID-19-related pneumonia, hospitalized and discharged.	Subjects unable to meet the criteria of acceptability and repeatability of functional investigations or who have not signed the informed consent	121	60.4	68.6	29.0	100	NR	NR
**Aparisi et al[3]**	2021	Spain	cohort	NR	< 18 years old, pregnancy, terminally ill patients, active SARS-CoV-2 infection, inability to exercise, and previous known severe cardiopulmonary disease.	70	54.8	35.7	27.2	75.7	NR	8
**Aranda et al[4]**	2022	Spain	cohort	> 18 years old admitted with RT-PCR-proven SARS-CoV-2 infection and COVID-19 pneumonia. Hospitalised and survived one year	Institutionalized patients or had been admitted to hospital at the time of follow-up, refused to participate, could not be contacted after	150	63	58	27.8	100	6.7	9

				after hospital discharge.	three calls, or lived outside the area							
**Aranda et al 2**[5]	2021	Spain	cohort	> 18 years old, RT-PCR proven SARS-CoV-2 infection and severe COVID-19 pneumonia who had ARDS during hospital admission and survived to hospital discharge	Institutionalized or admitted to hospital at follow-up, refused to participate, could not be contacted after three calls, and lived outside the area covered by the hospital	113	64	69.9	29.3	100	37.2	23
**Bardakci et al**[6]	2021	Turkey	cohort	>18 years old who were followed up, in COVID inpatient clinics; patients with SARS-CoV-2 PCR (+) and/or SARS-CoV-2 IgM/G (+); patients clinically and radiologically compatible with COVID-19 classed as severe	Patients who had neurological diseases, such as advanced dementia, Alzheimer's, SVD, etc; patients who had a history of orthopedic or other surgical operations, and consequently, were not able to take the walking test; and patients over 90 years old.	65	NR	75.4	NR	100	12.3	11.7
**Bellan et al**[7]	2021	Italy	cohort	Over 18, admitted and discharged for COVID-19	Pregnant, dead	200	62	61	27.5	100	11.5	9
**Bellan et al 2**[8]	2021	Italy	cohort	Over 18, admitted and discharged for COVID-19	Died	238	61	59.7	NR	100	11.8	NR

**Betschart et al[9]**	2021	Switzerland	cohort	RT-PCR positive for COVID, hospitalised for at least a day, more than 14 days ago and at least 4 days without COVID-19 related symptoms, over 18	Mental disability or impairments to reasoning or judgment, were immunocompromised due to medical treatment, or had a documented objection to subsequent use of their personal health data.	42	60	70	NR	100	NR	10
**Blanco et al (group 1)[10] (mild/moderate group)**	2021	Spain	cohort	>18 years old, admitted to hospital with PT-PCR positive COVID-19	Patients with a need for prior invasive mechanical ventilation, chronic infectious diseases, chronic lung diseases, concurrent autoimmune diseases or cancer, chronic use of corticosteroids or immunosuppressive therapy, pregnancy, alcohol/drug abuse, or patients whose condition did not allow participation in this study.	52	55.0	63.5	NR	100	NR	7
**Blanco et al (group 2)[10]- (Severe group)**						48	54.8	64.6	NR	100	NR	8

**Bretas et al[11]**	2022	Brazil	cohort	18 or over, admitted to three public referral hospitals for COVID-19 in Belo Horizonte, Minas Gerais, Brazil, with a confirmed diagnosis of COVID-19 positive RT-PCR result and severe acute respiratory syndrome (SARS)	Patients with indication for palliative care. Patients who were too weak to perform the tests, and those who withdrew consent.	211	60.8	51.7	NR	100	46.9	10
**Calvo-Paniagua et al[12]**	2022	Spain	quasi-experimental	Aged from 25 to 65 years, who had surpassed COVID-19, with a negative PCR test at the moment of the study and reporting fatigue and dyspnea as main post-COVID symptoms from at least three months after the infection	1) Patients with other post-COVID symptoms, 2) evidence of pluri-pathology 3) evidence of any medical co-morbidity 4) presence of fatal medical co-morbidities e.g., cancer; 5) immunodeficient patients; 6) previous history of dementia or psychiatric disorders; 7) patients with severe functional limitations or 8) patients with cognitive problems.	68	48.5	38.2	NR	44.1	4.4	7.7
**Cao et al[13]**	2021	China	cohort	Survivors with COVID-19 from a designated tertiary centre in China	Three patients were excluded due to age criteria	61	43.5	54	NR	100	NR	NR

**Capin et al[14]**	2022	USA	cohort	>18 years of age and had been hospitalized due to COVID-19 for more than 24 h between March 2020 and November 2020 and subsequently discharged home. Participants were required to speak and read either English or Spanish, be able to provide informed consent, and be able to access online questionnaires through a computer, tablet, or smart phone.	Participants requiring a higher level of care on discharge (e.g., skilled nursing facility)	109	53	46.8	NR	100	25.7	4
**Cassar et al[15]**	2021	UK	cohort	Moderate to severe acute respiratory distress syndrome. Coronavirus-2 (SARS-CoV-2 infection) positive RT-PCR nasopharyngeal swab test	Contraindications to MRI and severe comorbidities – end-stage renal, cardiac, liver, neurological disease	46	55.2	63	30.6	100	37	9
**Cherrez-Ojeda et al[16]**	2022	Ecuador	cohort	Past clinical diagnosis compatible with an acute SARS-CoV2 pulmonary infection, further confirmed by RT-PCR, dating >90 days.	NR	43	55.7	55.8	NR	25.6	NR	NR

**Clavario et al[17]**	2021	Italy	cohort	Consecutive patients undergoing post-COVID-19 evaluation post RT-PCR - confirmed SARS-CoV-2 infection admitted to COVID-19 wards.	Missing clinical data, unable to perform CPET testing	200	58.8	57	26.6	100	NR	17
**Combret et al[18]**	2022	France	cohort	All adult patients >18yrs old admitted for SARS-CoV-2 infection with positive RT-PCR test or lung computed tomography (CT) and clinical symptoms.	1) Patients who died during hospitalization or after hospital discharge; 2) length of stay< 24 h; 3) incidental asymptomatic infection 4) patients under guardianship.	128	69	51.6	27.6	100	17.2	9
**Damanti et al[19] (frail)**	2022	Italy	cohort	NR	NR	58	78	70.7	27.4	100	5.2	18.5
**Damanti et al[19] (robust)**						299	74	57.5	27.3	100	4.4	14
**Damanti et al 2[20]**	2021	Italy	cohort	COVID-19 inpatients treated with CPAP without previous intubation.	Patients with: i) chronically receiving CPAP for obstructive sleep apnoea; ii) previously intubated or requiring ICU during the same admission; iii) enrolled in a concomitant randomized trial on the use of early CPAP; iv) with severe contraindications to CPAP (e.g. coma or	67	62.8	85	28.5	100	NR	20.4

					hemodynamic instability)							
**Daynes et al[21]**	2021	UK	cohort	self-identified rehabilitation needs, whereby they displayed physical and/or psychological symptoms that were affecting their daily activities	demonstrated acute symptoms or were not medically stable or had only symptoms that were deemed not modifiable from a rehabilitation programme	30	58	52	NR	87	NR	10
**De Lorenzo et al[22]**	2022	Italy	cohort	Age ≥ 18 years individuals, who were not transferred to the intensive care unit (ICU)	NR	316	61.8	67.7	NR	100	0	11
**de Sousa et al[23]**	2022	Brazil	cohort	≥18 years old, had a previous diagnosis of COVID-19 confirmed by RTqPCR and diagnosis of PCS, and did not require hospitalization or intensive care unit (ICU) admission.	Patients with positive RT-qPCR at the time of inclusion in the study and those with neurological or musculoskeletal disease before COVID-19 were excluded.	40	35	40	27.2	0	0	0
**Del Brutto et al[24]**	2021	Ecuador	cohort	NR	NR	149	69.9	38	NR	NR	NR	NR

**Del Corral et al[25]**	2022	Spain	cohort	1) Confirmed SARS-CoV-2 infection; 2) non-hospital management; 3) age ≥18 years and 4) no more than 3 months since the infection.	1) Clinically evident cognitive impairment or active mental disorders; 2) difficulty understanding the language, or visual, abstraction or orientation impairment precluding the ability to complete the questionnaires; 3) presence of any concomitant condition that might affect the respiratory and/or functional state and 4) living outside the Community of Madrid.	102	46.6	37.3	26.4	0	NR	0
**Eberst et al[26]**	2022	France	cohort	SARS-CoV-2 infection diagnosed by viral RNA RT-PCR. Admitted to the ICU with SpO2 < 92% and evidence of air-space changes in 25% of lung parenchyma on chest CT scan.	>79 years. Chronic respiratory insufficiency, long-term oxygen therapy, interstitial lung disease, significant psychiatric disorders, or a life expectancy estimated at less than one year.	85	68.4	78.8	NR	100	100	44

**Evans et al 2[27]**	2022	UK	cohort	Patients aged over 18 years old who were discharged from one of 53 National Health Service (NHS) hospitals across England, Northern Ireland, Scotland and Wales following admission to a medical assessment or ward for confirmed or clinician-diagnosed COVID-19.	i) Had a confirmed diagnosis of a pathogen unrelated to the objectives of this study, ii) attended an accident and emergency department but were not admitted, iii) had another life-limiting illness with life expectancy less than six months such as disseminated malignancy	807	58.7	64.4	31.5	100	NR	17.8
**Evers et al[28]**	2022	Germany	cohort	NR	NR	30	51.5	60	27	70	NR	NR
**Faverio et al [29](Oxygen)**	2021	Italy	cohort	Age between 18 and 80 years Diagnosis of SARS-CoV-2 infection by positive PCR. Clinical/instrumental signs of interstitial pneumonia and acute respiratory failure (PaO2/FiO2 <300 in room air) on hospital admission. Written informed consent and discharged at home or in another hospital facility	Severe renal failure or NYHA class IV , upon discharge Pregnancy or breastfeeding Bacterial and/or fungal pulmonary superinfection during hospital stay. Prior diagnosis of chronic obstructive pulmonary disease, pulmonary emphysema, pulmonary fibrosis, or bronchiectasis.	71	61.1	56	27.5	100	NR	NR
**Faverio et al[29] (CPAP)**						144	61.1	77	28.7	100	NR	NR
**Faverio et al[29] (IMV)**						97	60.8	80	28.1	100	NR	NR

**Faverio et al 2 [30](oxygen)**	2022	Italy	cohort	Age between 18 and 80 years. Diagnosis of SARS-CoV-2 infection by positive PCR. Clinical/instrumental signs of interstitial pneumonia and acute respiratory failure (PaO_2/FiO_2 <300 in room air) on hospital admission. Written informed consent and discharged at home or in another hospital facility.	Severe renal failure or NYHA class IV, upon discharge Pregnancy or breastfeeding. Bacterial and/or fungal pulmonary superinfection during hospital stay. Prior diagnosis of chronic obstructive pulmonary disease, pulmonary emphysema, pulmonary fibrosis, or bronchiectasis.	61	60.7	54	27.4	100	NR	NR
**Faverio et al 2 [30](CPAP)**						136	60.7	78	28.7	100	NR	NR
**Faverio et al 2 [30](IMV)**						9	60.3	82	28.3	100	NR	NR
**Ferioli et al[31]**	2022	Italy	cohort	Previous molecular diagnosis of COVID-19 and an age older than 18 years.	Nil	100	60	55	26	100	NR	22
**Gianella et al[32]**	2021	Switzerland	cohort	39 consecutive laboratory-confirmed COVID-19 patients with pathological findings on a chest ultralow dose (uld) CT scan performed at hospital admission.	Age <18 years, pregnancy and absence of a written informed consent.	39	62.5	76.9	NR	100	25.6	15

**Gochicoa-Rangel et al[33] (noIMV)**	2021	Mexico	cohort	Subjects who survived severe pneumonia (who either did or did not require invasive mechanical ventilation), and reported their lung function as out-patients during convalescence.	NR	85	46	65	29.3	100	NR	10
**Gochicoa-Rangel et al[33] (IMV)**						86	47.3	63	28.8	100	NR	26
**Gonzalez et al[34]**	2022	Spain	cohort	All patients were positive for SARS-CoV-2, were older than 18 years and had been admitted to the ICU	(i) Treatment with palliative care, (ii) follow-up in another center, and (iii) severe mental disability that made it impossible to assess pulmonary function.	105	61	67.6	NR	100	100	23
**González-Islas et al[35]**	2022	Mexico	cohort	Moderate to severe COVID-19 patients with PCR confirmed diagnosis, > 18 years old, blood oxygen saturation ≤ 93% on room air, PaO2/FiO2 ratio of arterial partial pressure of oxygen to fraction of inspired oxygen < 300, who required hospitalization were discharged and, signed informed consent	Died before the follow-up visit, declined to participate, or could not be contacted.	530	53.8	60.9	NR	100	NR	17

**Guler et al (severe)**[36]	2021	Switzerland	cohort	Adults who survived acute COVID-19 and presented for clinical follow-up after either mild to moderate or severe to critical COVID-19.	NR	66	60.3	60.6	29.8	100	NR	NR
**Guler et al (mild)**[36]						47	52.9	57.4	25.5	100	NR	NR
**Gulhan et al**[37]	2022	Turkey	cohort	NR	NR	53	38	45.3	NR	17	NR	9
**Huang, C et al**[38]	2021	China	cohort	All patients with laboratory confirmed COVID-19 who were discharged	1) Those who died before the follow-up visit, 2) those for whom follow-up would be difficult owing to psychotic disorder, dementia, or re-admission to hospital attributed to underlying diseases, 3) those who were unable to move freely due to concomitant osteoarthropathy or immobile before or after discharge due to diseases such as stroke or pulmonary embolism, 4) those who declined to participate, 5) those unable to be contacted, and 6) those living outside of Wuhan or in nursing or welfare homes	1733	57	52	NR	100	4	14

**Huang, L. et al 2**[39]	2022	China	cohort	All patients with laboratory confirmed COVID-19 who were discharged	1) Those who died before the follow-up visit, 2) those for whom follow-up would be difficult owing to psychotic disorder, dementia, or re-admission to hospital attributed to underlying diseases, 3) those who were unable to move freely due to concomitant osteoarthropathy or immobile before or after discharge due to diseases such as stroke or pulmonary embolism, 4) those who declined to participate, 5) those unable to be contacted, and 6) those living outside of Wuhan or in nursing or welfare homes	1192	57	54	NR	100	4	14

**Huang, L et al[40]**	2021	China	cohort	All patients with laboratory confirmed COVID-19 who were discharged	1) those who died before the follow-up visit, 2) those for whom follow-up would be difficult owing to psychotic disorder, dementia, or re-admission to hospital attributed to underlying diseases, 3) those who were unable to move freely due to concomitant osteoarthropathy or immobile before or after discharge due to diseases such as stroke or pulmonary embolism, 4) those who declined to participate, 5) those unable to be contacted, and 6) those living outside of Wuhan or in nursing or welfare homes	1276	59	53	NR	100	4	14
**Jacobson et al[41]**	2021	USA	cohort	NR	NR	118	43.3	53.4	30.4	18.6	9.3	7.5
**Janssen et al[42] (Control)**	2021	Netherlands	cohort	Patient with COVID-19- associated hyperinflammation defined by: oxygen saturation at rest ≤94% or tachypnoea (>30/min); and at least two out of the	Unable to visit the outpatient clinic	52	63	83	30.3	100	NR	NR
**Janssen et al[42] (treated)**						66	65	79	27.8	100	NR	NR

				following three biomarker criteria: C reactive protein >100 mg/L, serum ferritin >900 µg/L at one occasion or a twofold increase of the level at admission within 48 hours and D- dimer level >1500 µg/L								
**Jimeno-Almazan et al[43] (control)**	2022	Spain	RCT	Aged over 18 who had a confirmed diagnosis of COVID-19 by SARS-CoV2 PCR. Who presented a chronic symptomatic phase, lasting >12 weeks from the onset of symptoms, and had not been hospitalized because of the acute COVID- 19 infection.	Pregnant patients and those who had acute or unstable chronic diseases such as unstable myocardiopathy, ischemic heart disease, uncontrolled hypertension, uncontrolled chronic obstructive pulmonary disease (COPD), or major surgery in the past 3 months.	20	46	20	NR	0	0	0
**Jimeno-Almazan et al[43] (Exercise)**						19	44.6	32	NR	0	0	0
**Johnsen et al[44]**	2021	Denmark	cohort	NR	NR	57	51	49	27	59.6	NR	13.3
**Joris et al[45]**	2022	Belgium	cohort	ICU admission	NR	31	61	67.7	32.9	100	100	29
**Karoli et al (mod)[46]**	2022	India	cohort	Patients of moderate to severe COVID 19 who had RT-PCR test-confirmed SARS-CoV-2 infection and required hospitalization	NR	64	55	45	22.7	100	NR	NR
**Karoli et al (severe)[46]**						38	57	66	25.2	100	NR	NR

**Kattainen et al[47]**	2022	Finland	cohort	Age ≥ 18 years and Finnish as their primary language.	Major prior neurological diseases such as Parkinson's disease, dementia, traumatic brain injury, stroke, and developmental disability or pregnant	85	60	61	30.1	100	100	20
**Kersten et al[48]**	2022	Germany	cohort	NR	NR	367	47.3	42.5	25.8	6.8	NR	NR
**Kooner et al[49]**	2022	Canada	cohort	Individuals aged 18–80 years, a local public health office confirmed (positive-test) case of COVID-19, persistent symptoms up to 3 months post infection, including but not limited to respiratory, neurological and metabolic systems, and a clinical diagnosis of PACS. Inclusion criteria for Site 2 consisted of people aged 18 or older who recently (≤12 weeks) recovered from COVID- 19 with the date of recovery confirmed in accordance with provincial and local public health unit protocols.	NR	76	53	50	30	30.3	NR	13

**Ladlow et al[50]**	2022	UK	cohort	UK Service Personnel, previous COVID-19 infection- severe illness at time of acute illness requiring hospital admission, positive for COVID-19 antigen PCR test at time of acute illness, or clinically adjudicated COVID-19, negative for COVID-19 antigen PCR test at time of recruitment to study, >4 weeks since resolution of fever/acute illness	Past medical history of cardiac or pulmonary disease, unwilling or unable to give informed consent, active acute infection at the time of recruitment as determined by clinical assessment, pregnant at the time of recruitment to the study, unable to meet the criteria of the MRI safety questionnaire, unable, or unwilling, to complete any research investigations	113	37	82	29	0	0	0
**Ladlow et al 2[51]**	2022	UK	cohort	Hospitalization, life-limiting symptoms beyond 12 weeks, desaturation 95% on a Harvard step test, or chest pain with electrocardiographic [ECG] changes during acute illness	NR	205	38	83.9	29	NR	NR	NR
**Lam et al[52]**	2021	Canada	cohort	NR	NR	165	50.7	NR	30.7	40	NR	NR
**Landi et al[53]**	2022	Italy	cohort	Enrolled 33 subjects suffering from fatigue at the time of baseline visit and who had received the prescription for a multicomponent oral	NR	66	61	56	24.9	50	12	15.7

				nutritional supplement								
**Larsson et al[54]**	2022	Sweden	cohort	Previously been included in an ongoing research project investigating the acute effects of COVID-19	NR	46	59	74	29	100	100	22
**Latronico et al[55]**	2021	Italy	cohort	Consecutive critically ill adult patients (≥18 years old) with confirmed SARS- CoV-2 infection and ARDS3 discharged alive.	NR	114	60	77	27	100	NR	29
**Levy et al[56]**	2022	France	cohort	NR	NR	139	62	68	29	100	71	21
**Liao et al[57]**	2022	China	cohort	NR	1) Having a history of chronic lung disease, 2) having a history of psychiatric disorders, 3) being SARS-CoV-2 nucleic acid repositive, and 4) refusing to participate	303	39	19.5	23.4	100	NR	15

**Longobardi et al**[58]	2022	Brazil	cohort	Survivors of severe COVID-19, aged ≥ 45 yr, with diagnosis confirmed by RT-PCR for SARS-CoV-2 and that had been discharged from intensive care unit (ICU) treatment at our tertiary referral hospital between 3 and 6mo before the study.	Individuals with inability to walk, unstable angina, need for oxygen supply or resting oxygen saturation of <85% while breathing room air, anemia, pulmonary hypertension, recent myocardial infarction (<12 mo), severe valve disease, unstable angina, untreated heart failure, uncontrolled arrhythmias, uncontrolled hypertension, active oncological disease or recent malignancy (<5 yr), transplant history, uncontrolled type 2 diabetes, and/or autoimmune diseases.	35	59	63	30.1	100	100	19
**Lorent et al**[59]	2022	Belgium	cohort	Consecutive adult patients, who were admitted hospital with COVID-19, and were seen in the outpatient clinic at 3 and 12 months after discharge.	Residents of a medical care facility, patients with cognitive impairment or those with a geriatric profile (clinical frailty scale >4)	299	59	69	27.4	100	36	10
**Marando et al**[60]	2022	Switzerland	cohort	NR	<18 years, pregnancy and absence of a	39	64.5	79	NR	100	26.3	15

					written informed consent.							

**Margalit et al (no fatigue)[61]**	2022	Israel	cohort	At least 2 months following a PCR–proven diagnosis of COVID-19.	ICU admission or required IMV during acute admission. Individuals diagnosed with PE/pulmonary infarction, on current oxygen supplementation, with sleep apnea/hypopnea syndrome or with post-COVID-19 chest imaging suggesting pulmonary fibrosis. Individuals with heart failure, ACS or percutaneous cardiac intervention during the year prior to the diagnosis. Individuals who underwent surgical procedure under general anesthesia during the year prior to the diagnosis. Individuals with cerebrovascular disease and a history of stroke or TIA. Individuals with paralysis of the lower limbs, amputees, bedridden, those assisted with walking aids or wheelchair. Individuals with	75	42.7	43.7	27.5	10.7	NR	9.3
**Margalit et al (fatigue)[61]**						66	48.9	39.4	27.5	9.1	NR	8.8

					myopathies or myositis, degenerative neurological or muscular diseases, and myasthenia gravis. Individuals with pre-COVID-19 diagnosis of chronic fatigue syndrome, fibromyalgia, major depressive disorder, necessitating pharmacotherapy. Immunosuppressed. Individuals with uncontrolled thyroid disease. Pregnant women. Chronic use of opioids.							
**Martino et al[62]**	2022	Italy	cohort	Diagnosis of severe COVID-19 based on the WHO interim guidance; laboratory confirmation of SARS-CoV-2 infection using RT-PCR; hospitalization because of severe disease.	Unwilling to be followed up at our institution, if they were unable to sign the informed consent or comply with the study procedures.	64	68	64	26	100	NR	NR

**McNarry et al (control)[63]**	2022	UK	RCT	Prior self-reported COVID-19 infection; primary symptom of breathlessness; aged ≥18 years.	i) Dementia meaning they could not follow commands/training; ii) unstable cardiac disease, myocardial infarction or non-ST-elevation myocardial infarction within six weeks; and/or iii) high risk of falls.	37	46.1	5	27.8	NR	NR	NR
**McNarry et al (intervention)[63]**						111	46.8	14	27.6	NR	NR	NR
**Mittal et al[64]**	2021	India	cohort	Patients with COVID-19 who suffered from mild symptoms and underwent home quarantine or had moderate to severe symptoms and were hospitalized or intensive care unit.	Having history of any other prolonged illness other than COVID-19 and severe complications of diabetes	52	55.2	47.8	27.8	41.5	36	NR
**Motiejunaite et al[65]**	2021	France	cohort	NR	NR	114	57	67	27.8	91	22	10
**Muller et al[66]**	2022	Switzerland	cohort	Participants aged ≥50 years	NR	288	65.1	65	28.7	71	44	NR
**Nanwani-Nanwani et al[67]**	2022	Spain	cohort	≥ 18 years old admitted to the ICU due to severe SARS-CoV-2 infection, requiring IMV and who were alive at the time of hospital discharge	Previous severe psychiatric conditions, cognitive deficits, and any sort of functional dependency, patients from a different geographical area and patients who refused to sign the informed consent form.	186	59	68	31	100	100	54

**Noel-Savina et al[68]**	2021	France	cohort	COVID-19 pneumonia diagnosed by PCR. Hospitalization, with clinical and radiological lung involvement: saturation below 94% without respiratory support and CT of the chest, presenting common features of COVID-19 pneumonia. Patients had to be 18 years old or more, and give a written informed consent.	Lung disease not documented on CT of the chest, negative COVID-19 PCR, and prior cardiopulmonary disease that could itself provoke abnormal gas exchange.	72	60.5	76.4	NR	100	76.1	NR
**O'Brien et al[69]**	2022	Ireland	cohort	All patients who were referred to the clinic physiotherapy service, hospitalisation for COVID-19 (confirmed by positive SARS-CoV-2 PCR), ability to complete study questionnaires in English and aged ≥ 18 years.	NR	55	58.6	57	NR	100	26	13.1
**Plekhanova et al[70]**	2022	UK	cohort	Patients aged ≥ 18 years who were discharged from 83 National Health Service (NHS) hospitals across England, Northern Ireland, Scotland, and Wales following	i) Had a confirmed diagnosis of a pathogen unrelated to the objectives of this study, ii) attended an accident and emergency department but were not admitted, iii) had	512	59	64.6	31	100	NR	NR

				admission to a medical assessment or ward for confirmed or clinician-diagnosed COVID-19.	another life-limiting illness with life expectancy less than six months.							
**Pogosova et al[71]**	2022	Russia	cohort	NR	NR	213	57	55.4	29.4	100	8.5	NR
**Evans et al 1[72]**	2021	UK	cohort	Patients aged over 18 years old who were discharged from one of 53 National Health Service (NHS) hospitals across England, Northern Ireland, Scotland and Wales following admission to a medical assessment or ward for confirmed or clinician-diagnosed COVID-19.	i) Had a confirmed diagnosis of a pathogen unrelated to the objectives of this study, ii) attended an accident and emergency department but were not admitted, iii) had another life-limiting illness with life expectancy less than six months such as disseminated malignancy	1077	57.9	64.3	30.1	100	NR	9
**Ribeiro Baptista et al[73]**	2022	France	cohort	Diagnosed with severe COVID-19 pulmonary infection, confirmed by positive PCR or serology, hospitalized at intensive care unit (ICU) or at conventional care unit for more than 7 days	Under 18 years old or pregnant.	105	59.2	75.2	27.9	100	43.3	NR

				and with oxygen therapy during hospitalization (> 3 l/min)								
**Rinaldo et al**[74]	2021	Italy	cohort	1) Age >18 years, 2) previous molecular diagnosis of SARS-CoV-2 infection	Absence of a signed informed consent, acute respiratory exacerbation in the previous 4 weeks and the presence of medical conditions contraindicating CPET	75	57	57	28.6	100	NR	NR
**Riou et al (mild)**[75]	2021	France	cohort	NR	NR	21	58	67	28.7	100	0	NR
**Riou et al (severe)**[75]						15	63	80	30.1	100	0	NR
**Riou et al (critical)**[75]						45	63	73	28	100	100	NR
**Rousseau et al**[76]	2021	Belgium	cohort	All consecutive critically ill COVID-19 patients admitted to ICU during the first wave	Still hospitalized in an inpatient rehabilitation facility, or if they were unable to communicate in the French language	32	62	72	31.5	100	100	40
**Saluja et al**[77]	2022	India	cohort	Lab-confirmed RT-PCR positive report, age between 18 years to 70 years, history of hospitalisation or treatment in home-	Patients with any gross chest deformity, prior respiratory, cardiac, mental, or neurological illness, BMI > 28 kg/m^2	166	41.2	88	NR	42.8	NR	NR

				isolation 2 months ago or more	current or past smoking							
**Schandl et al**[78]	2021	Sweden	cohort	Survivors of severe COVID- 19 infection who had been admitted to either of the two ICUs. All patients with a positive PCR for COVID- 19 and treated for respiratory failure with invasive ventilation, highflow treatment with oxygen (HFNO) or non- invasive ventilation (NIV) in the ICU	Dead	113	58	76.1	28	100	100	NR
**Sevillano-Castano et al**[79]	2022	Spain	cohort	Patients > 18 years old; diagnosis of COVID-19 by positive PCR and pulmonary rehabilitation candidates by post-COVID-19 condition, defined as individuals with a history of probable or confirmed SARS-CoV-2 infection, usually 3 months from the onset of COVID-19 with symptoms that	Presence of locomotor or cognitive impairment before the infection; refusal to participate; and any pre-existing condition, such as orthopaedic or neurological comorbidities, limiting the ability to perform the standard field test.	42	53.8	48	29.3	70	16.7	15

				lasted for ⩾2 months and cannot be explained by an alternative diagnosis								
**Shi et al (MSC)[80]**	2022	China	RCT	(1) Severe COVID19 diagnosed after onset of disease; (2) chest computed tomography (CT) imaging confirmed pneumonia combined with lung damage.	Patients with shock or COVID-19 combined with any one of other organ failures, those who received invasive ventilation, or patients with any malignant tumor, pregnancy or breastfeeding, or coinfection of other pathogens.	65	60.7	56.9	24.7	100	NR	NR
**Shi et al (placebo)[80]**						35	59.9	54.3	25	100	NR	NR
**Shogenova et al (control)[81]**	2021	Russia	RCT	1) Staff of the D. D. Plet nev City Clinical Hospital (Moscow); 2) age >18 years; 3) negative SARS-CoV-2 nucleic acid test using the polymerase chain reaction at the time of the study; 4) documented CT of the lung with COVID-19 signs; 5) 2 major and at least 6 minor diagnostic signs of	1) Need of respiratory support; 2) acute and decompensated heart failure; 3) acute and exacerbated chronic respiratory failure; 4) stroke during the prior 6 months; 5) myocardial infarction during the prior 6 months; 6) decompensated diabetes ; 7) pregnancy; 8) long term (>14 days)	30	51	13.3	30	100	NR	NR

**Shogenova et al (intervention)[81]**				CFS. 6) no signs of respiratory disorders.	immunosuppressive therapy during the prior 6 months; 9) prior cancer.	30	53	16.7	29	100	NR	NR
**Sivori et al (mod)[82]**	2022	Argentina	cohort	Patients >18 years old, with a confirmed diagnosis of SARS-CoV-2 disease determined by PCR, hospitalized in the general ward or ICU, with pneumonia imaging criteria on admission.	Unable to sign or did not want to give their consent to participate in the study	94	58.4	58.5	30	100	NR	14
**Sivori et al (severe)[82]**						100	57.5	70	30	100	NR	24
**Sneller et al[83]**	2022	USA	cohort	Adults with laboratory-confirmed SARS-CoV-2 infection, at least 6 weeks past onset of COVID-19 symptoms, no fever within 7 days before enrollment, and did not have worsening respiratory symptoms. Persons with asymptomatic disease were eligible 4 weeks after the first positive SARS-CoV-2 RT-PCR test result	NR	189	50	45	28.8	11.6	NR	NR
**Sperling et al[84]**	2022	Denmark	cohort	Patients ≥18 years old hospitalized for COVID-19 with a confirmed SARS-CoV-2 infection by PCR	NR	218	59.9	59	29.2	100	19	9.3

				testing. Informed consent.								
**Staudt et al**[85]	2022	Germany	cohort	NR	Not capable of informed consent, positive PCR test without clinical correlate, not traceable, or without sufficient proficiency in German or English language	101	60	58.4	27.5	100	20	8
**Steinbeis et al**[86]	2022	Germany	cohort	NR	NR	54	56	59.3	26.2	87.9	46.3	NR
**Tauekelova et al**[87]	2022	Kazakhstan	cohort	1)≥ 18 years old; 2) at least one persistent symptom for at least 3 months after the first onset of COVID-19 infection	NR	312	54	32.4	26.9	35.6	NR	NR
**Townsend et al**[88]	2021	Ireland	cohort	All individuals with a positive SARS-CoV-2 nasopharyngeal swab PCR at our institution, including both those hospitalised and those managed in the community during their acute illness. Minimum of six weeks following resolution of symptoms or hospital discharge.	Outpatient appointments were not offered to residents in long term care facilities.	71	44.3	30	NR	25	8	NR

**Uz et al[89]**	2021	Turkey	cohort	Patients over 18 years of age, who were not bedridden, not on a mechanical ventilator, cognitively intact cooperative and, ambulatory with or without support.	Patients with a mechanical ventilator, being bedridden or wheelchair dependent, unable to cooperate, with known progressive/non-progressive neurological disease, patients with previously diagnosed pulmonary disease, previous surgery and a trauma history of the lower extremities and vertebrae, as well as those with a history of malignancy and inflammatory disease.	99	49.1	53.5	27.9	100	NR	4
**van den Borst et al[90]**	2021	Netherlands	Cohort	Consecutive patients who had been discharged after inpatient treatment for COVID-19. General practitioner (GP) referrals of RT-PCR-confirmed or clinically suspect SARS-CoV-2 patients without hospitalization but with symptoms persisting >6 weeks.	NR	124	59	60	NR	76.4	NR	8
**Van Gassel et al[91]**	2021	Netherlands	cohort	All patients admitted to ICU requiring	Died	46	62	69.6	27.7	100	100	32

				mechanical ventilation due to COVID-19								
**Vannini et al**[92]	2021	Spain	cohort	Patients > 18 and <75 years old without severe comorbidities.	NR	41	57.3	61	30.4	100	NR	NR
**Vejen et al**[93]	2022	Denmark	cohort	1) SARS-CoV-2 infection verified by RT-PCR, 2) Primary diagnosis of COVID-19, 3) Admitted with COVID-19, and 4) Signs of COVID-19 pneumonitis on chest X-ray, computed tomography scan and/or requiring supplemental oxygen.	1) Age < 18 years, 2) Not able to attend the follow-up visit due to severe disability or chronic cognitive deficits, or 3) Deceased prior to the intended time of follow-up.	128	64.5	58	27.2	100	NR	5
**Wahlgren et al**[94]	2022	Sweden	cohort	Ongoing concerning residual symptoms and limitations in activity	Fatalities, coincidental cases, and cases with premorbid conditions precluding assessment of COVID-19-attributable sequels.	158	57.4	61.4	NR	100	NR	7
**Wu, Q et al**[95]	2021	China	cohort	NR	Patients who (1) died before the follow-up, (2) refused to participate in the follow-up, and (3) left the local area and could not complete the follow-up.	132	42	55	NR	100	NR	NR

**Wu, X et al[96]**	2021	China	cohort	At least 18 years old with severe COVID-19 discharged from hospital.	Patients with a history of hypertension, diabetes, cardiovascular disease, cancer, and chronic lung disease, including asthma or COPD, or a history of smoking documented at time of hospital admission. Patients who required intubation and mechanical ventilation.	83	60	57	25	100	NR	29
**Xiong et al[97]**	2021	China	cohort	NR	1) Without admission information, 2) missing information on comorbidities, and 3) mild or moderate COVID-19.	333	36	23	23.5	100	5	NR
**Xiong et al 2[98]**	2022	China	cohort	Healthcare workers of “Rehabilitation Care Project for Medical Staff Infected with COVID-19” who completed any follow-up visits	NR	446	35	23	22.8	100	3	NR

**Yang et al**[99]	2022	China	cohort	All critically ill adult patients receiving invasive or non-invasive mechanical ventilation, high-flow nasal oxygen therapy, or vasopressors	(i) Died before the first follow-up; (ii) had dementia, psychotic disorders, or other neurological dysfunctions leading to inability to communicate before the admission or after discharge; (iii) unable to mobilise freely because of severely impaired cardiopulmonary function, disabilities (unable to walk), or sequalae at admission and after discharge; and (iv) prolonged hospitalisation or chronic lung disease before SARS-CoV-2 infection	92	60	56.6	23.9	100	NR	28
**Yildirim et al (ICU)**[100]	2021	Turkey	cohort	Patients who were followed up on for more than 48 hours in ICU and more than 72 hours in medical wards due to COVID-19.	1) Those who have neurodegenerative diseases, 2) those who were readmitted to the hospital due to any other conditions, 3) those with impaired movement due to physical disabilities.	31	59	84	31	100	100	19
**Yildirim et al (non-ICU)**[100]						39	56	62	30	100	0	8
**Zhang et al**[101]	2022	China	cohort	Participants with WHO scale 3 to 6	NR	288	55	61.1	24.6	100	12.5	14

				severity COVID-19 infection								
**Zhao et al**[102]	2021	China	cohort	All adult patients with laboratory-confirmed SARS-CoV-2 infection, and subsequently admitted to hospital	NR	94	48.1	57.5	NR	100	11.7	15.1
**Zhou et al**[103]	2021	China	cohort	Enrolled laboratory-confirmed COVID-19 patients discharged from hospital.	Patients without considerable data due to physiological or subjective rejection tests	120	51.6	40.8	24.6	100	NR	25.5
**Anastasio et al**[104]	2021	Italy	cohort	COVID-19 diagnosis by PCR, between 18 and 80 years old	Previous diagnosis of pulmonary disease, excluding asthma.	379	56	45.9	25.2	NR	NR	NR
**Morin et al**[105]	2021	France	cohort	Survival 4 months after hospital discharge or after intensive care unit (ICU) discharge, who were older than 18 years, who had been hospitalized for >24 hours primarily because of COVID-19, and who had received a diagnosis of SARS-CoV-2 RT-PCR, by typical CT lung scan associated with clinical features, or both.	Death within 4 months after discharge, persistent hospitalization, end-stage cancer, dementia, nosocomial COVID-19 infection, and incidental positive SARS-CoV-2 RT-PCR result during a hospital stay for a different medical indication	177	56.9	61.6	29.1	100	54.8	13
**Shah et al**[106]	2021	Canada	cohort	Adults with COVID-19 hospitalised with laboratory-confirmed SARS-CoV-2 infection.	NR	60	67	68	25	100	NR	10

Supplementary Table 2: Summary of outcome measures in each study. Abbreviations: six minute walk test (6MWT), Incremental shuttle walk test (ISWT), Short Physical Performance Battery (SPPB), One minute sit to stand (1minSTS), Handgrip strength (HGS), Two Minute Walk Test(2MWT), Five sit-to-stand(5STS), Manual muscle test(MMT)

Study	Median follow up time (months)	Outcome						
		6MWT	ISWT	SPPB	1min STS	HGS	CPET	Other
**Abdallah et al[1]**	4.3						x	
**Aiello et al[2]**	3.6	x						
**Aparisi et al[3]**	6.5	x					x	
**Aranda et al[4]**	14.1	x						
**Aranda et al 2[5]**	8.5	x						
**Bardakci et al[6]**	7.0	x						
**Bellan et al[7]**	12.0			x				2MWT
**Bellan et al 2[8]**	3.5			x				2MWT
**Betschart et [9]**	3.5; 12.5	x						
**Blanco et al[10]**	3.7	x						
**Bretas et al[11]**	6.4	x						
**Calvo-Paniagua et al[12]**	4.7	x						
**Cao et al[13]**	3.0	x						
**Capin et al[14]**	3.0; 4.5							Chair rise test
**Cassar et al[15]**	6.0						x	

**Cherrez-Ojeda et al[16]**	5.7	x						
**Clavario et al[17]**	3.8	x			x		x	
**Combret et al[18]**	11.0				x			
**Damanti et al[19]**	3.0; 6.0			x		x		
**Damanti et al 2[20]**	6.0	x						
**Daynes et al[21]**	4.5		x					
**De Lorenzo et al[22]**	6.7	x						
**de Sousa et al[23]**	8.0					x		
**Del Brutto et al[24]**	8.0					x		
**Del Corral et al[25]**	3.0; 6.5				x	x		
**Eberst et al[26]**	3.0; 6.0; 12.0	x						
**Evans et al 2[27]**	5.0; 13.0		x	x				
**Evers et al[28]**	4.3; 7.4						x	
**Faverio et al[29]**	6.0	x						
**Faverio et al 2[30]**	12.0	x						
**Ferioli et al[31]**	6.0	x						
**Gianella et al[32]**	3.0	x						
**Gochicoa-Rangel et al[33]**	3.0	x						
**Gonzalez et al[34]**	3.0; 6.0; 12.0	x						
**González-Islas et al[35]**	3.0					x		
**Guler et al[36]**	4.6	x						
**Gulhan et al[37]**	3.2	x						
**Huang, C et al[38]**	5.5	x						
**Huang, L. et al 2[39]**	24.5	x						
**Huang, L et al[40]**	12.5	x						
**Jacobson et al[41]**	4.3	x						
**Janssen et al[42]**	3.0; 6.0	x						
**Jimeno-Almazan et al[43]**	8.3					x	x	5STS
**Johnsen et al[44]**	3.0				x	x	x	
**Joris et al[45]**	3.0						x	
**Karoli et al[46]**	3.4	x						
**Kattainen et al[47]**	6.4	x						

**Kersten et al[48]**	3.0	x						
**Kooner et al[49]**	3.5	x						
**Ladlow et al[50]**	5.7						x	
**Ladlow et al 2[51]**	6.5						x	
**Lam et al [52]**	4.3	x						
**Landi et al[53]**	3.4	x			x	x		
**Larsson et al[54]**	4.0; 12.0	x						
**Latronico et al[55]**	3.0; 6.0; 12.0	x				x		
**Levy et al[56]**	3.0					x		
**Liao et al[57]**	14.1	x						
**Longobardi et al[58]**	6.1						x	
**Lorent et al[59]**	13.8	x				x		Quadriceps strength
**Marando et al[60]**	3.0; 12.0	x						
**Margalit et al[61]**	7.6						x	
**Martino et al[62]**	12.0	x						
**McNarry et al[63]**	9.0						x	
**Mittal et al[64]**	3.3					x		
**Motiejunaite et al[65]**	3.0						x	
**Muller et al[66]**	3.9	x		x		x		30 second STS
**Nanwani-Nanwani et al[67]**	3.1					x		
**Noel-Savina et al[68]**	4.3	x						
**O'Brien et al[69]**	8.6; 15.4	x						
**Plekhanova et al[70]**	8.8							Accelerometer
**Pogosova et al[71]**	5.0	x						
**Evans et al 1[72]**	5.0		x	x				
**Ribeiro Baptista et al[73]**	3.0	x				x	x	
**Rinaldo et al[74]**	3.5						x	
**Riou et al[75]**	3.0,6.0	x						
**Rousseau et al[76]**	3.4					x		Quadriceps strength
**Saluja et al[77]**	3.9	x						
**Schandl et al[78]**	5.0	x						

**Sevillano-Castano et al[79]**	5.8				x			
**Shi et al[80]**	3.2; 6.1; 9.5; 12.9							
**Shogenova et al[81]**	7.0	x						
**Sivori et al[82]**	3.0; 6.0	x						
**Sneller et al[83]**	5.3	x						
**Sperling et al[84]**	4.6	x						
**Staudt et al[85]**	11.0	x						
**Steinbeis et al[86]**	12.0						x	
**Tauekelova et al[87]**	5.8	x						
**Townsend et al[88]**	3.6	x						
**Uz et al[89]**	3.0	x						
**van den Borst et al[90]**	3.3	x						
**Van Gassel et al[91]**	4.3	x				x		
**Vannini et al[92]**	6.0						x	
**Vejen et al[93]**	5.0	x						
**Wahlgren et al[94]**	5.1	x						
**Wu, Q et al[95]**	6.0							MMT
**Wu, X et al[96]**	5.5; 6.8; 9.8; 12.4	x						
**Xiong et al[97]**	5.5; 8.7; 11.8							Muscle strength test, chair stand
**Xiong et al 2[98]**	13.8					x		Chair stand, step test
**Yang et al[99]**	3.0; 6.0; 12.0	x						
**Yildirim et al[100]**	7.1	x						
**Zhang et al[101]**	6.8; 12.8; 24.1	x						
**Zhao et al[102]**	13.1	x						
**Zhou et al[103]**	11.2	x						
**Anastasio et al[104]**	4.8	x						
**Morin et al[105]**	4.5	x						
**Shah et al[106]**	3.0	x						

Supplementary Table 3: Quality scoring of included studies.

Study	Aim/objective of the study clearly described?	Patient characteristics described?	Single site or multi-site	Representativeness of the cohort	Controls present?	Loss to follow up documented?	Main findings of the study clearly described?	Adjustments made for confounders?	Publication peer reviewed?	Total
	1 = Yes, 0 = No	1 = Yes, 0 = No	1 = Multi-site, 0 = Single site	2 = Truly representative, 1 = Selected cohort, 0 = No description	1 = Yes, 0 = No	1 = Yes, 0 = No	1 = Yes, 0 = No	1 = Yes, 0 = No	1 = Yes, 0 = No	/10
**Abdallah et al**[1]	1	1	0	2	0	1	1	1	1	8
**Aiello et al**[2]	1	1	0	1	0	0	1	1	1	6
**Aparisi et al**[3]	1	1	0	1	0	0	1	1	1	6
**Aranda et al**[4]	1	1	1	2	0	0	1	0	1	7
**Aranda et al 2**[5]	1	1	0	1	0	1	1	1	1	7
**Bardakci et al**[6]	1	1	0	1	0	1	1	1	1	7
**Bellan et al**[7]	1	1	0	1	0	1	0	1	1	6
**Bellan et al 2**[8]	1	1	0	1	0	1	1	0	1	6

**Betschart et [9]**	1	1	0	1	0	1	1	0	1	6
**Blanco et al[10]**	1	1	1	1	0	0	1	1	1	7
**Bretas et al[11]**	1	1	1	1	0	1	1	1	1	8
**Calvo-Paniagua et al[12]**	1	1	1	1	0	1	1	0	1	7
**Cao et al[13]**	1	1	0	1	0	1	1	1	1	7
**Capin et al[14]**	1	1	0	1	0	1	1	1	1	7
**Cassar et al[15]**	1	1	0	1	1	1	1	0	1	7
**Cherrez-Ojeda et al[16]**	1	0	0	1	0	0	1	0	1	4
**Clavario et al[17]**	1	0	0	1	0	1	1	1	1	6
**Combret et al[18]**	1	1	0	1	0	1	1	1	1	7
**Damanti et al[19]**	1	0	0	0	0	1	1	1	1	5
**Damanti et al 2[20]**	1	1	0	1	0	1	1	0	1	6
**Daynes et al[21]**	1	1	0	1	0	1	1	1	1	7
**De Lorenzo et al[22]**	1	1	0	1	0	1	0	1	1	6

**de Sousa et al[23]**	1	1	0	1	1	1	1	1	1	8
**Del Brutto et al[24]**	1	0	0	1	1	1	1	1	1	7
**Del Corral et al[25]**	1	1	0	1	0	1	1	1	1	7
**Eberst et al[26]**	1	1	0	1	0	1	1	1	1	7
**Evans et al 2[27]**	1	1	1	1	0	1	1	1	1	8
**Evers et al[28]**	1	0	0	1	0	0	1	1	1	5
**Faverio et al[29]**	1	1	1	1	0	1	1	1	1	8
**Faverio et al 2[30]**	1	1	1	1	0	1	1	1	1	8
**Ferioli et al[31]**	1	1	0	1	0	1	1	1	1	7
**Gianella et al[32]**	1	1	0	1	0	0	1	0	1	5
**Gochicoa-Rangel et al[33]**	1	1	0	1	0	0	1	1	1	6
**Gonzalez et al[34]**	1	1	0	1	0	1	1	1	1	7
**González-Islas et al[35]**	1	1	0	1	0	0	1	1	1	6
**Guler et al[36]**	1	0	1	0	0	0	1	0	1	4
**Gulhan et al[37]**	1	0	0	1	0	1	1	1	1	6

**Huang, C et al[38]**	1	1	0	1	0	1	1	0	1	6
**Huang, L. et al 2[39]**	1	1	0	1	0	1	1	1	1	7
**Huang, L et al[40]**	1	1	0	1	0	1	1	1	1	7
**Jacobson et al[41]**	1	0	0	0	0	0	1	1	1	4
**Janssen et al[42]**	1	1	0	1	0	1	1	1	1	7
**Jimeno-Almazan et al[43]**	1	1	0	1	0	0	1	1	1	6
**Johnsen et al[44]**	1	0	0	0	0	1	0	1	1	4
**Joris et al[45]**	1	1	0	1	1	1	1	1	1	8
**Karoli et al[46]**	1	1	0	1	0	0	1	1	1	6
**Kattainen et al[47]**	1	1	0	1	0	0	1	1	1	6
**Kersten et al[48]**	1	0	0	2	0	1	1	1	1	7
**Kooner et al[49]**	1	1	1	2	1	1	1	1	1	10
**Ladlow et al[50]**	1	1	0	1	1	1	1	1	1	8
**Ladlow et al 2[51]**	1	1	0	1	0	0	1	1	1	6
**Lam et al [52]**	1	0	0	0	0	0	0	1	1	3
**Landi et al[53]**	1	0	0	0	0	0	1	1	1	4

**Larsson et al[54]**	1	0	0	1	0	1	1	0	1	5
**Latronico et al[55]**	1	1	0	1	0	1	1	1	1	7
**Levy et al[56]**	1	0	0	1	0	0	1	1	1	5
**Liao et al[57]**	1	0	0	1	0	1	1	1	1	6
**Longobardi et al[58]**	1	1	0	1	1	0	1	1	1	7
**Lorent et al[59]**	1	1	1	1	0	1	1	1	1	8
**Marando et al[60]**	1	1	0	1	0	1	1	1	1	7
**Margalit et al[61]**	1	1	0	1	0	1	1	1	1	7
**Martino et al[62]**	1	1	0	2	0	1	1	1	1	8
**McNarry et al[63]**	1	1	0	1	0	1	1	1	1	7
**Mittal et al[64]**	1	1	0	1	1	0	1	1	1	7
**Motiejunaite et al[65]**	1	0	0	0	0	0	1	1	1	4
**Muller et al[66]**	1	1	0	1	0	1	0	1	1	6
**Nanwani-Nanwani et al[67]**	1	1	2	1	0	1	1	1	1	9
**Noel-Savina et al[68]**	1	1	0	1	0	1	1	1	1	7

**O'Brien et al[69]**	1	1	0	1	0	1	1	0	1	6
**Plekhanova et al[70]**	1	1	1	1	0	1	1	1	1	8
**Pogosova et al[71]**	0	0	0	0	0	1	1	1	1	4
**Evans et al 1[72]**	1	1	1	0	0	1	1	1	0	6
**Ribeiro Baptista et al[73]**	1	1	0	1	0	1	1	1	1	7
**Rinaldo et al[74]**	1	1	0	1	0	0	1	1	1	6
**Riou et al[75]**	1	0	0	1	0	0	1	1	1	5
**Rousseau et al[76]**	1	1	0	1	0	1	1	1	1	7
**Saluja et al[77]**	1	1	0	1	1	0	1	1	1	7
**Schandl et al[78]**	1	1	1	1	0	1	1	1	1	8
**Sevillano-Castano et al[79]**	1	1	0	1	0	0	1	0	1	5
**Shi et al[80]**	1	1	0	1	0	1	1	1	1	7
**Shogenova et al[81]**	1	1	0	1	0	0	1	0	1	5
**Sivori et al[82]**	1	1	1	1	0	1	1	1	1	8
**Sneller et al[83]**	1	1	1	1	1	0	1	1	1	8

**Sperling et al[84]**	1	1	0	1	0	0	1	1	1	6
**Staudt et al[85]**	1	0	0	1	0	1	1	1	1	6
**Steinbeis et al[86]**	1	0	0	0	0	1	0	1	1	4
**Tauekelova et al[87]**	1	0	1	1	0	0	0	1	1	5
**Townsend et al[88]**	1	1	0	1	1	0	1	1	1	7
**Uz et al[89]**	1	1	0	1	0	1	1	1	1	7
**van den Borst et al[90]**	1	1	0	1	0	1	1	1	1	7
**Van Gassel et al[91]**	1	1	0	1	0	1	1	1	1	7
**Vannini et al[92]**	1	1	0	1	0	1	1	1	1	7
**Vejen et al[93]**	1	1	0	1	0	1	1	1	1	7
**Wahlgren et al[94]**	1	1	1	1	0	1	0	1	1	7
**Wu, Q et al[95]**	1	0	0	1	0	1	1	1	1	6
**Wu, X et al[96]**	1	1	0	0	0	1	1	1	1	6
**Xiong et al[97]**	1	1	1	1	0	1	1	0	1	7
**Xiong et al 2[98]**	1	1	1	1	0	1	1	1	1	8
**Yang et al[99]**	1	1	1	1	0	1	1	1	1	8

**Yildirim et al[100]**	1	1	0	1	0	1	1	1	1	7
**Zhang et al[101]**	1	1	0	1	0	1	1	1	1	7
**Zhao et al[102]**	1	1	1	1	0	1	1	1	1	8
**Zhou et al[103]**	1	1	1	1	0	0	1	1	1	7
**Anastasio et al[104]**	1	1	1	1	0	0	1	1	1	7
**Morin et al[105]**	1	1	0	1	0	1	1	0	1	6
**Shah et al[106]**	1	0	0	1	0	1	1	1	1	6

References

1. Abdallah SJ, Voduc N, Corrales-Medina VF et al. Symptoms, pulmonary function, and functional capacity four months after covid-19. *Ann Am Thorac Soc*.2021;18:1912–1917. https://doi.org/10.1513/AnnalsATS.202012-1489RL .
2. Aiello M, Marchi L, Calzetta L et al. Coronavirus disease 2019: COSeSco– a risk assessment score to predict the risk of pul monary sequelae in COVID-19 patients. *Respiration*. 2022;101: 272–280. https://doi.org/doi:10.1159/000519385.
3. Aparisi A, Ybarra-falcon C, Garcia-gomez M et al. Exercise ventilatory inefficiency in post-covid-19 syndrome: insights from a prospective evaluation. *J Clin Med*. 2021;10:2591. https://doi.org/10.3390/jcm10122591 .

4. Aranda J, Oriol I, Feria L et al. Persistent COVID-19 symptoms 1 year after hospital discharge: a prospective multicenter study. *PLoS One*. 2022;17:e0275615. https://doi.org/10.1371/journal.pone.0275615 .
5. Aranda J, Oriol I, Martin M et al. Long-term impact of COVID 19 associated acute respiratory distress syndrome. *J Inf Secur*. 2021;83:581–588. https://doi.org/10.1016/j.jinf.2021.08.018 .
6. Bardakci MI, Ozturk EN, Ozkarafakili MA, Ozkurt H, Yanc U, Yildiz Sevgi D. Evaluation of long-term radiological findings, pulmonary functions, and health-related quality of life in survivors of severe COVID-19. *J Med Virol*. 2021;93:5574–5581. https://doi.org/10.1002/jmv.27101 .
7. Bellan M, Baricich A, Patrucco F et al. Long-term sequelae are highly prevalent one year after hospitalization for severe COVID-19. *Sci Rep*. 2021;11:22666. https://doi.org/10.1038/s41598-021-01215-4 .
8. Bellan M, Soddu D, Balbo PE et al. Respiratory and psychophysical sequelae among patients with COVID-19 four months after hospital discharge. *JAMA Netw Open*. 2021;4:e2036142. https://doi.org/doi:10.1001/jamanetworkopen.2020.36142.
9. Betschart M, Rezek S, Unger I et al. One year follow-up of physical performance and quality of life in patients surviving COVID-19: a prospective cohort study. *Swiss Med Wkly*. 2021;151:w30072. https://doi.org/10.4414/SMW.2021.w30072 .
10. Blanco JR, Cobos-Ceballos MJ, Navarro F et al. Pulmonary long-term consequences of COVID-19 infections after hospital discharge. *Clin Microbiol Infect*. 2021;27:892–896. https://doi.org/10.1016/j.cmi.2021.02.019
11. Bretas DC, Leite AS, Mancuzo EV et al. Lung function six months after severe COVID-19: does time, in fact, heal all wounds? *Braz J Infect Dis*. 2022;26:102352. https://doi.org/10.1016/j.bji d.2022.102352.
12. Calvo-Paniagua J, Diaz-Arribas MJ, Valera-Calero JA et al. A tele-health primary care rehabilitation program improves self-perceived exertion in COVID-19 survivors experiencing post-COVID fatigue and dyspnea: a quasi-experimental study. *PLoS One*. 2022;17: e0271802. https://doi.org/10.1371/journal.pone.0271802.
13. Cao J, Zheng X, Wei W et al. Three-month outcomes of recovered COVID-19 patients: prospective observational study. *Ther Adv Respir Dis*. 2021;15:175346662110094. https://doi.org/10.1177/17534666211009410.
14. Capin JJ, Wilson MP, Hare K et al. Prospective telehealth analysis of functional performance, frailty, quality of life, and mental health after COVID-19 hospitalization. *BMC Geriatr*. 2022;22:251. https://doi.org/10.1186/s12877-022-02854-6
15. Cassar MP, Tunnicliffe EM, Petousi N et al. Symptom persistence despite improvement in cardiopulmonary health- insights from longitudinal CMR, CPET and lung function testing post COVID-19. *EClinicalMedicine*. 2021;41:101159. https://doi.org/10.1016/j.eclinm.2021.101159
16. Cherrez-Ojeda I, Sanchez-Angarita E ,Vanegas E et al. Pulmonary evaluation of post-COVID-19 patients: An Ecuadorian experience. *J Community Hosp Intern Med Perspect*. 2022;12:30–34. https://doi.org/10.55729/2000-9666.1031
17. Clavario P, De Marzo V, Lotti R et al. Cardiopulmonary exercise testing in COVID-19 patients at 3 months follow-up. *Int J Cardiol.* 2021;340:113–118. https://doi.org/10.1016/j.ijca rd.2021.07.033
18. Combret Y, Kerne G, Pholoppe F et al. Remote assessment of quality of life and functional exercise capacity in a cohort of COVID-19 patients one year after hospitalization (TELECOVID). *J Clin Med*. 2022;11:905. https://doi.org/10.3390/jcm11040905

19. Damanti S, Cilla M, Cilona M et al. Prevalence of long COVID-19 symptoms after hospital discharge in frail and robust patients. *Front Med*. 2022;9:9. https://doi.org/10.3389/fmed.2022.834887
20. Damanti S, Ramirez GA, Bozzolo EP et al. Six-month respiratory outcomes and exercise capacity of COVID-19 acute respiratory failure patients treated with continuous positive airway pressure. *Intern Med J*. 2021;51:1810–1815. https://doi.org/doi:10.1111/imj.15345
21. Daynes E, Gerlis C, Chaplin E, Gardiner N, Singh SJ. Early expe riences of rehabilitation for individuals post-COVID to improve fatigue, breathlessness exercise capacity and cognition- a cohort study. *Chronic Respir Dis*. 2021;18:147997312110156. https://doi.org/10.1177/14799731211015691
22. De Lorenzo R, Magnaghi C, Cinel E et al. A nomogram-based model to predict respiratory dysfunction at 6 months in non critical COVID-19 survivors. *Front Med*. 2022;9:781410. https://doi.org/10.3389/fmed.2022.781410.
23. de Sousa KCA, Gardel DG, Lopes AJ. Postural balance and its association with functionality and quality of life in non-hospitalized patients with post-acute COVID-19 syndrome. *Physiother Res Int*. 2022;27:e1967. https://doi.org/10.1002/pri.1967
24. Del Brutto OH, Mera RM, Pérez P et al. Hand grip strength before and after SARS-CoV-2 infection in community-dwelling older adults. *J Am Geriatr Soc*. 2021;69:2722–2731. https://doi.org/doi:10.1111/jgs.17335
25. Del Corral T, Menor-Rodriguez N et al. Longitudinal study of changes observed in quality of life, psychological state cognition and pulmonary and functional capacity after COVID-19 infection: a six- to seven-month prospective cohort. *J Clin Nurs*. 2022;33:89–102. https://doi.org/10.1111/jocn.16352
26. Eberst G, Claude F ,Laurent L et al. Result of one-year ,prospective follow-up of intensive care unit survivors after SARS-CoV-2pneumonia. *Ann Intensive Care*. 2022;12:23. https://doi.org/10.1186/s13613-022-00997-8
27. Evans RA, Leavy OC, Richardson M et al. Clinical characteristics with inflammation profiling of long COVID and association with 1-year recovery following hospitalisation in the UK: a prospective observational study. *Lancet Respir Med*. 2022;10:761–775. https://doi.org/10.1016/S2213-2600(22)00127-8
28. Evers G, Schulze AB, Osiaevi I et al. Sustained impairment in cardiopulmonary exercise capacity testing in patients after COVID 19: a single center experience. *Can Respir J*. 2022;2022:1, 2466789–11. https://doi.org/10.1155/2022/2466789
29. Faverio P, Luppi F, Rebora P et al. Six-month pulmonary impairment after severe COVID-19: a prospective, multicentre follow-up study. *Respiration*. 2021;100:1078–1087. https://doi.org/doi:10.1159/000518141.
30. Faverio P, Luppi F, Rebora P et al. One-year pulmonary impairment after severe COVID-19: a prospective, multicenter follow up study. *Respir Res*. 2022;23:65. https://doi.org/10.1186/s12931-022-01994-y
31. Ferioli M, Prediletto I, Bensai S et al. Spontaneous evolution of COVID-19 lung sequelae: results from a double step follow-up. *Respiration*. 2022;101:381–393. https://doi.org/doi:10.1159/000521316.
32. Gianella P, Rigamonti E, Marando M et al. Clinical, radio logical and functional outcomes in patients with SARS-CoV-2 pneumonia: a prospective observational study. *BMC Pulm Med*. 2021;21:136. https://doi.org/10.1186/s12890-021-01509-3

33. Gochicoa-Rangel L, Hernández-Morales AP, Salles-Rojas A et al. Gas exchange impairment during COVID-19 recovery. *Respir Care*. 2021;66:1610–1617. https://doi.org/doi:10.4187/respcare.09114
34. Gonzalez J, Zuil M, Benitez ID et al. One year overview and follow-up in a post-COVID consultation of critically ill patients. *Front Me*d.2022;9:897990. https://doi.org/10.3389/fmed.2022.897990
35. González-Islas D, Sánchez-Moreno C, Orea-Tejeda A et al. Body composition and risk factors associated with sarcopenia in post COVID patients after moderate or severe COVID-19 infections. *BMC Pulm Med*.2022;22:223–228. https://doi.org/doi:10.1186/s12890-022-02014-x
36. Guler SA, Ebner L, Aubry-Beigelman C et al. Pulmonary function and radiological features 4 months after COVID-19: first results from the national prospective observational Swiss COVID 19 lung study. *Eur Respir J*. 2021;57:2003690. https://doi.org/10.1183/13993003.03690-2020
37. Gülhan PY, Arbak PM, Annakkaya AN, Balbay EG, Balbay ÖA. An assessment of post-COVID-19 infection pulmonary functions in healthcare professionals. *Am J Infect Control*. 2022;50:1125–1132. https://doi.org/doi:10.1016/j.ajic.2022.07
38. Huang C, Huang L, Li X et al. 6-month consequences of COVID-19 in patients discharged from hospital: a cohort study. *Lancet*.2021;397:220–232. https://doi.org/10.1016/S0140-6736(20)32656-8
39. Huang L, Li X, Gu X et al. Health outcomes in people 2 years after surviving hospitalisation with COVID-19: a longitudinal cohort study. *Lancet Respir Med.*2022;10:863–876. https://doi.org/10.1016/S2213-2600(22)00126-6
40. Huang L ,Yao Q, Gu X et al. 1-year outcomes in hospital survivors with COVID-19: a longitudinal cohort study. *Lancet*. 2021;398: 747–758. https://doi.org/10.1016/S0140-6736(21)01755-4
41. Jacobson KB, Rao M, Bonilla H et al. Patients with uncomplicated coronavirus disease 2019 (COVID-19) have long-term persistent symptoms and functional impairment similar to patients with severe COVID-19: a cautionary tale during a global pandemic. *Clin Infect Dis*. 2021;73:E826–E829. https://doi.org/10.1093/cid/ciab103
42. Janssen MTHF, Ramiro S, Mostard RLM, Magro-Checa C, Landewé RBM. Three-month and six-month outcomes of patients with COVID-19 associated hyperinflammation treated with short-term immunosuppressive therapy: follow-up of the CHIC study. *RMD Open*. 2021;7:e001906. https://doi.org/10.1136/rmdopen-2021-001906
43. Jimeno-Almazan A, Franco-Lopez F, Buendia-Romero A et al. Rehabilitation for post-COVID-19 condition through a super vised exercise intervention: a randomized controlled trial. *Scand J Med Sci Sports*. 2022;32:1791–1801. https://doi.org/10.1111/sms.14240
44. Johnsen S, Sattler SM, Miskowiak KW et al. Descriptive analysis of long COVID sequelae identified in a multi disciplinary clinic serving hospitalised and non-hospitalised patients. *ERJ Open Res*. 2021;7:00205–02021. https://doi.org/10.1183/23120541.00205-2021
45. Joris M, Pincemail J, Colson C et al. Exercise limitation after critical versus mild COVID-19 infection: a metabolic perspective. *J Clin Med*. 2022;11:4322. https://doi.org/10.3390/jcm11154322
46. Karoli R, Gupta N, Shakya S. Follow-up study of pulmonary function, exercise capacity and radiological changes after recovery from moderate to severe COVID pneumonia without mechanical ventilation. *J Assoc Physicians India*. 2022;69: 11–12

47. Kattainen S, Lindahl A, Vasankari T et al. Lung function and exercise capacity 6 months after hospital discharge for critical COVID-19. BMC Pulm Med. 2022;22:243. https://doi.org/10.1186/s12890-022-02023-w
48. Kersten J, Wolf A, Hoyo L et al. Symptom burden correlates to impairment of diffusion capacity and exercise intolerance in long COVID patients. *Sci Rep*.2022;12:8801. https://doi.org/10.1038/s41598-022-12839-5
49. Kooner HK, McIntosh MJ, Matheson AM et al.129 Xe MRI ventilation defects in ever-hospitalised and never-hospitalised people with post-acute COVID-19 syndrome. *BMJ Open Respir Res*. 2022;9:e001235. https://doi.org/10.1136/bmjresp-2022-001235
50. Ladlow P, O'Sullivan O, Bennett AN et al. The effect of medium term recovery status after COVID-19 illness on cardiopulmonary exercise capacity in a physically active adult population. *J Appl Physiol*. 2022;132:1525–1535.
51. Ladlow P, O'Sullivan O, Houston A et al. Dysautonomia following COVID-19 is not associated with subjective limitations or symptoms but is associated with objective functional limitations. *Heart Rhythm*. 2022;19:613–620. https://doi.org/doi:10.1016/j.hrthm.2021.12.005
52. Lam GY, Befus AD, Damant RW et al. Exertional intolerance and dyspnea with preserved lung function: an emerging long COVID phenotype? *Respir Res*. 2021;22:222. https://doi.org/10.1186/s12931-021-01814-9
53. Landi F, Martone AM, Ciciarello F et al. Effects of a new multicomponent nutritional supplement on muscle mass and physical performance in adult and old patients recovered from COVID-19: a pilot observational case-control study. *Nutrients*. 2022;14:2316. https://doi.org/10.3390/nu14112316
54. Larsson IM, Hultstrom M, Lipcsey M et al. Poor long-term recovery after critical COVID-19 during 12 months longitudinal follow-up. *Intensive Crit Care Nurs*. 2022;74:103311. https://doi.org/10.1016/j.iccn.2022.103311
55. Latronico N, Peli E, Calza S et al. Physical, cognitive and mental health outcomes in 1-year survivors of COVID-19-associated ARDS. *Thorax*. 2021;77:300–303. https://doi.org/10.1136/thora xjnl-2021-218064
56. Levy D, Giannini M, Oulehri W et al. Long term follow-up of sarcopenia and malnutrition after hospitalization for COVID-19 in conventional or intensive care units. *Nutrients*. 2022;14:912. https://doi.org/doi:10.3390/nu1404091
57. Liao T, Meng D, Xiong L et al. Long-term effects of COVID-19 on health care workers 1-year post-discharge in Wuhan. *Infect Dis Ther*. 2022;11:145–163. https://doi.org/10.1007/s40121-021-00553-0
58. Longobardi I, Prado DMLD, Goessler KF et al. Oxygen uptake kinetics and chronotropic responses to exercise are impaired in survivors of severe COVID-19. *Am J Phys*. 2022;323:H569 H576. https://doi.org/10.1152/ajpheart.00291.2022
59. Lorent N, Weygaerde YV, Claeys E et al. Prospective longitudinal evaluation of hospitalised COVID-19 survivors 3 and 12 months after discharge. *ERJ Open Res*. 2022;8:00004–02022. https://doi.org/10.1183/23120541.00004-2022
60. Marando M, Fusi-Schmidhauser T, Tamburello A et al. 1 year radiological, functional and quality-of-life outcomes in patients with SARS-CoV-2 pneumonia—a prospective observational study. *NPJ Primary Care Respir Med*. 2022;32:8. https://doi.org/10.1038/s41533-022-00273-z
61. Margalit I, Yelin D, Sagi M et al. Risk factors and multidimensional assessment of long COVID fatigue: a nested case control study. *Clin Infect Dis*. 2022;75:1688–1697. https://doi.org/10.1093/cid/ciac283

62. Martino GP, Benfaremo D, Bitti G et al. 6 and 12 month outcomes in patients following COVID-19-related hospitalization: a prospective monocentric study. *Int Emerg Med*. 2022;17: 1641–1649. https://doi.org/10.1007/s11739-022-02979-x
63. McNarry MA, Berg RMG, Shelley J et al. Inspiratory muscle training enhances recovery post-COVID-19: a randomised controlled trial. *Eur Respir J.* 2022;60:2103101. https://doi.org/10.1183/13993003.03101-2021
64. Mittal J, Ghosh A, Bhatt SP, Anoop S, Ansari IA, Misra A. High prevalence of post COVID-19 fatigue in patients with type 2 diabetes: a case-control study. *Diabetes Metab Syndr*. 2021;15:102302. https://doi.org/10.1016/j.dsx.2021.102302
65. Motiejunaite J, Balagny P, Arnoult F et al. Hyperventilation as one of the mechanisms of persistent dyspnoea in SARS CoV-2 survivors. *Eur Respir J*. 2021;58:2101578. https://doi.org/10.1183/13993003.01578-2021
66. Muller I, Mancinetti M, Renner A et al. Frailty assess ment for COVID-19 follow-up: a prospective cohort study. BMJOpenRespirRes.2022;9:e001227. https://doi.org/10.1136/bmjresp-2022-001227
67. Nanwani-Nanwani K, Lopez-Perez L, Gimenez-Esparza C, et al. Prevalence of post-intensive care syndrome in mechanically ventilated patients with COVID-19. *Sci Rep* 2022;12:7977. https://doi.org/10.1038/s41598-022-11929-8
68. Noel-Savina E, Viatge T, Faviez G et al. Severe SARS-CoV-2pneumonia: clinical, functional and imaging outcomes at 4 months. *Respir Med Res*. 2021;80:100822. https://doi.org/10.1016/j.re smer.2021.100822
69. O'Brien K, Townsend L, Dowds J et al. 1-year quality of life and health-outcomes in patients hospitalised with COVID-19: a longitudinal cohort study. *Respir Res*. 2022;23:1–11. https://doi.org/10.1186/s12931-022-02032-7
70. Plekhanova T, Rowl SAV et al. Device-assessed sleep and physical activity in individuals recovering from a hospital admission for COVID-19: a multicentre study. *Int J Behav Nutr Phys Activity*. 2022;19:94–13. https://doi.org/doi:10.1186/s12966-022-01333-w
71. Pogosova NV, Paleev FN, Ausheva AK et al. Sequelae of COVID-19 at long-term follow-up after hospitalization. *Ration Pharmacother Cardiol.* 2022;18:118–126. https://doi.org/10.20996/1819-6446-2022-04-03
72. Evans R, Rahman NM et al .Physical, cognitive, and mental health impacts of COVID-19 after hospitalisation (PHOSP-COVID): a UK multicentre, prospective cohort study. *Lancet Respir Med*. 2021;9:1275–1287. https://doi.org/10.1016/S2213-2600(21)00383-0
73. Ribeiro Baptista B, d'Humières T, Schlemmer F et al. Identification of factors impairing exercise capacity after severe COVID 19 pulmonary infection: a 3-month follow-up of prospective COVulnerability cohort. *Respir Res*.2022;23:68–69. https://doi.org/doi:10.1186/s12931-022-01977-z
74. Rinaldo RF, Mondoni M, Parazzini EM et al. Severity does not impact on exercise capacity in COVID-19 survivors. *Respir Med*. 2021;187:106577. https://doi.org/10.1016/j.rmed.2021.106577
75. Riou M, Marco TC, Oulehri W et al. Respiratory follow-up after hospitalization for COVID-19: who and when? *EurJClin Investig*. 2021;51:e13603. https://doi.org/10.1111/eci.13603
76. Rousseau AF, Minguet P, Colson C et al. Post-intensive care syndrome after a critical COVID-19: cohort study from a Belgian follow-up clinic. *Ann Intensive Care*. 2021;11:1–9. https://doi.org/10.1186/s13613-021-00910-9

77. Saluja M, Meena D, Bhargava G et al. Spectrum of post-Covid-19 syndrome—post-hospitalisation Covid-19 study. *J Ind Acad Clin Med*. 2022;23:17–24.
78. Schandl A, Hedman A, Lynga P et al. Long-term consequences in critically ill COVID-19 patients: a prospective cohort study. *Acta Anaesthesiol Scand*. 2021;65:1285–1292. https://doi.org/10.1111/aas.13939
79. Sevillano-Castano A, Peroy-Badal R, Torres-Castro R et al. Is there a learning effect on 1-min sit-to-stand test in post-COVID 19 patients? *ERJ Open Res*. 2022;8:00189–02022. https://doi.org/10.1183/23120541.00189-2022
80. Shi L, Yuan X, Yao W et al. Human mesenchymal stem cells treatment for severe COVID-19: 1-year follow-up results of a randomized, double-blind, placebo-controlled trial. *EBioMedicine*. 2022;75:103789. https://doi.org/doi:10.1016/j.e biom.2021.103789
81. Shogenova LV, Tuet TT, Kryukova NO et al. KalHydrogen inhalation in rehabilitation program of the medical staff recovered from COVID-19. *Cardiovasc Ther Prevent*. 2021; 20:2986–2932. https://doi.org/doi:10.15829/1728-8800-2021 2986
82. Sivori M, Gonzalez A, Saldarini F et al. Clinical, functional and tomographic respiratory sequels of patients hospitalized for COVID-19 at six month of diagnosis: SECUELAR-19 trial. *Medicin*a. 2022;82:673–683
83. Sneller MC, Jason Liang C, Marques AR et al. A longitudinal study of COVID-19 sequelae and immunity: baseline findings. *Ann InternMed*. 2022;175:969–979. https://doi.org/10.7326/M21-4905
84. Sperling S, Floe A, Leth S et al. Fatigue is a major symptom at COVID-19 hospitalization follow-up. *J Clin Med*. 2022;11:2411. https://doi.org/10.3390/jcm11092411
85. Staudt A, Jorres RA, Hinterberger T et al. Associations of post acute COVID syndrome with physiological and clinical measures 10 months after hospitalization in patients of the first wave. *Eur J Int Med*. 2022;95:50–60. https://doi.org/10.1016/j.ejim.2021.10.031
86. Steinbeis F, Knape P, Mittermaier M et al. Functional limitations 12 months after SARS-CoV-2 infection correlate with initial disease severity: an observational study of cardiopulmonary exercise capacity testing in COVID-19 convalescents. *Respir Med*. 2022;202:106968. https://doi.org/10.1016/j.rmed.2022.106968
87. Tauekelova A, Kalila Z, Akerke B et al. Association of lung fibrotic changes and cardiological dysfunction with hypertension in long COVID-19 cohort. *medRxiv*. 2022;14: 106968
88. Townsend L, Dyer A H, Naughton A et al. Longitudinal analysis of COVID-19patients shows age-associated T cell changes independent of ongoing ill-health. Front Immunol. 2021;12:12. https://doi.org/10.3389/fimmu.2021.676932
89. Uz C, Umay E, Gundogdu I et al. Factors affecting medium-term aerobic capacity in COVID-19 patients. *Acta Medica Mediterranea*. 2021;37:847–853
90. van den Borst B, Peters JB, Brink M et al. Comprehensive health assessment 3 months after recovery from acute coronavirus disease 2019 (COVID-19). *Clin Infect Dis*. 2021;73:e1089. https://doi.org/doi:10.1093/cid/ciaa1750
91. Van Gassel RJJ, Bels J, Remij L et al. Functional outcomes and their association with physical performance in mechanically ventilated coronavirus disease 2019 survivors at 3 months following hospital discharge: a cohort study. *Crit Care Med*. 49:1726–1738. https://doi.org/10.1097/CCM.0000000000005089

92. Vannini L, Quijada-Fumero A, Martin MPR et al. Cardiopulmonary exercise test with stress echocardiography in COVID 19 survivors at 6 months follow-up. *Eur J Int Med*. 2021;94: 101–104. https://doi.org/10.1016/j.ejim.2021.10.004
93. Vejen M, Hansen EF, Al-Jarah BNI et al. Hospital admission for COVID-19 pneumonitis-long-term impairment in quality of life and lung function. *Eur Clin Respir J*. 2022;9:2024735. https://doi.org/10.1080/20018525.2021.2024735
94. Wahlgren C, Divanoglou A, Larsson M et al. Rehabilitation needs following COVID-19: five-month post-discharge clinical follow-up of individuals with concerning self-reported symptoms. *EClinicalMedicine*. 2022;43:101219. https://doi.org/10.1016/j.eclinm.2021.101219
95. Wu Q, Hou X, Li H et al. A follow-up study of respiratory and physical function after discharge in patients with redetectable positive SARS-CoV-2 nucleic acid results following recovery from COVID-19. *Int J Infect Dis*. 2021;107:5–11. https://doi.org/10.1016/j.ijid.2021.04.020
96. Wu X, Liu X, Zhou Y et al. 3-month, 6-month, 9-month, and 12-month respiratory outcomes in patients following COVID 19-related hospitalisation: a prospective study. *Lancet Respir Med*. 2021;9:747–754. https://doi.org/10.1016/S2213-2600(21)00174-0
97. Xiong L, Li Q, Cao X et al. Dynamic changes of functional fitness, antibodies to SARS-CoV-2 and immunological indicators within 1 year after discharge in Chinese health care workers with severe COVID-19: a cohort study. *BMCMed*.2021;19:163–111. https://doi.org/10.1186/s12916-021-02042-0
98. Xiong L, Li Q, Cao X et al. Recovery of functional fitness, lung function, and immune function in healthcare workers with non severe and severe COVID-19 at 13 months after discharge from the hospital: a prospective cohort study. *Int J Infect Dis*. 2022;123:119–126. https://doi.org/10.1016/j.ijid.2022.06.052
99. Yang X, Li Z, Wang B et al. Prognosis and antibody profiles in survivors of critical illness from COVID-19: a prospective multicentre cohort study. *Br J Anaesth*. 2022;128:491–500. https://doi.org/10.1016/j.bja.2021.11.024
100. Yildirim S, Ediboglu O, Susam S, et al. Do Covid-19 patients needing ICU admission have worse 6 months follow up outcomes when compared with hospitalized non-ICU patients? A prospective cohort study. Paper presented at: Intensive Care Medicine Experimental Conference: European Society of Intensive Care Medicine Annual Congress, Vol. 9. ESICM, 2021
101. Zhang H, Li X, Huang L et al. Lung-function trajectories in COVID-19 survivors after discharge: a two-year longitudinal cohort study. *eClinical Medicine*. 2022;54:101668. https://doi.org/10.1016/j.eclinm.2022.101668
102. Zhao Y, Yang C, An X et al. Follow-up study on COVID-19 survivors one year after discharge from hospital. *Int J Infect Dis*. 2021;112:173–182. https://doi.org/10.1016/j.ijid.2021.09.017
103. Zhou F, Tao M, Shang L et al. Assessment of sequelae of COVID 19 nearly 1 year after diagnosis. F*ront Med*. 2021;8:8. https://doi.org/10.3389/fmed.2021.717194
104. Anastasio F,Barbuto S,Scarnecchia E et al.Medium-term impact of COVID-19 on pulmonary function, functional capacity and quality of life. *Eur Respir J.* 2021;58:2004015. https://doi.org/10.1183/13993003.04015-2020 .
105. Morin L, Savale L, Pham T et al. Four-month clinical status of a cohort of patients after hospitalization for COVID-19. *JAMA*. 2021;325:1525–1534. https://doi.org/10.1001/jama.2021.3331

106. Shah AS, Wong AW, Hague CJ et al. A prospective study of 12-week respiratory outcomes in COVID-19-related hospitalisations. *Thorax*. 2021;76:402–404. https://doi.org/10.1136/thora xjnl-2020-216308
